# Isoselective Polymerization of *rac*‐Lactide by Aluminum Complexes of N‐Heterocyclic Carbene‐Phosphinidene Adducts

**DOI:** 10.1002/chem.202100482

**Published:** 2021-03-03

**Authors:** Jayeeta Bhattacharjee, Marius Peters, Dirk Bockfeld, Matthias Tamm

**Affiliations:** ^1^ Institut für Anorganische und Analytische Chemie Technische Universität Braunschweig Hagenring 30 38106 Braunschweig Germany

**Keywords:** aluminum, N-heterocyclic carbenes, phosphinidenes, poly(lactic acid), ring-opening polymerization

## Abstract

The N‐heterocyclic carbene‐phosphinidene adducts (NHC)PH were reacted with AlMe_3_ in toluene to afford the monoaluminum complexes [{(IDipp)PH}AlMe_3_] and [{(IMes)PH}AlMe_3_] (IDipp=1,3‐bis(2,6‐diisopropylphenyl)imidazolin‐2‐ylidene, IMes=1,3‐bis(2,4,6‐trimethylphenyl)imidazolin‐2‐ylidene). In contrast, the dialuminum complex [{(^Me^IMes)PH}(AlMe_3_)_2_] was obtained for ^Me^IMes=1,3‐bis(2,4,6‐trimethylphenyl)‐4,5‐dimethylimidazolin‐2‐ylidene. These complexes served as initiators for the efficient ring‐opening polymerization of *rac*‐lactide in toluene at 60 °C. High degrees of isoselectivity were found for the poly(*rac*‐lactide) obtained in the presence of the monoaluminum complexes (*P*_m_ up to 0.92, *T*_m_ up to 191 °C), whereas almost atactic polymers were produced by the dialuminum complex. Detailed mechanistic studies reveal that the polymerization proceeds via a coordination‐insertion mechanism with the carbene‐phosphinidene ligands acting as stereodirecting groups.

## Graphical abstract

**N‐Heterocyclic** carbene‐phosphinidene trimethylaluminum (AlMe_3_) complexes can promote the isoselective ring‐opening polymerization of *rac*‐lactide, which proceeds via insertion into the aluminum‐carbon bonds with the monodentate, prochiral phosphorus ligand acting as a stereo‐directing group.

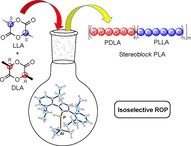

Polylactide (PLA), a biodegradable polymer derived from renewable resources such as corn or sugar beets, has found widespread use as a material for packaging, drug delivery and biomedical applications.^[1]^ Several methods are available for the synthesis of PLA, with metal‐catalyzed ring‐opening polymerization (ROP) of the cyclic dimer lactide (LA) being the most common route.[^2^, ^3^] Commercially available PLA is generally homochiral poly(l‐lactide) (PLLA), which is synthesized by ROP of l‐lactide (LLA). The resulting polymer has a high degree of crystallinity,^[4]^ with a melting temperature (*T*_m_) of 162–180 °C, which can be significantly increased by 40–50 °C through formation of a stereocomplex with poly(d‐lactide) (PDLA).^[5]^ Thus, stereocomplexation enhances the thermal resistance as well as the crystallinity of the PLA material,^[6]^ which presently requires parallel ROP of l‐lactide (LLA) and d‐lactide (DLA), followed by blending or cocrystallization of the homochiral polylactide chains.^[7]^ A simpler approach to high‐melting PLA materials is based on the racemic mixture of LLA and DLA, *rac*‐lactide, and its stereocontrolled ROP that can afford PLA stereoblock copolymers (Scheme 1), in which an increase in crystallinity is achieved by intermolecular interaction of the PLLA and PDLA segments.^[8]^ However, ROP of *rac*‐lactide cannot only lead to isotactic stereocomplex and stereoblock polymers, but also to heterotactic and atactic polymers.^[3]^ Therefore, the development of catalysts for the isoselective polymerization of *rac*‐lactide has received enormous attention in the past,^[9]^ involving a large number of transition,^[10]^ main group,^[11]^ and rare earth^[12]^ metal complexes as well as organo‐catalysts.^[13]^

**Scheme 1:**
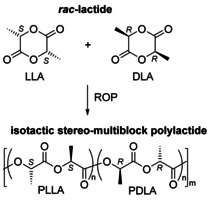
Ring‐opening polymerization (ROP) of a racemic mixture of (*S*,*S*)‐lactide (l‐lactide, LLA) and (*R*,*R*)‐lactide (d‐lactide, DLA) to produce isotactic polylactide with PLLA and PDLA stereo‐multiblocks.

In this context, aluminum complexes have played a particularly prominent role, and based on early work by Spassky and co‐workers,^[14]^ numerous chiral or achiral aluminum complexes, in which the metal atom is supported by O,N,N,O‐tetradentate salen‐ or salan‐type ligands, have been employed as catalyst for stereocontrolled ROP of *rac*‐lactide.[^15^, ^16^, ^17^, ^18^, ^19^] Highly stereoselective ROP has been achieved with *T*_m_ and *P*_m_ values up to 210 °C and 0.98, respectively,^[18]^ where *P*_m_ refers to the probability of *meso* enchainment. Despite these significant advances, little effort has been made to identify other suitable ligand systems for aluminum complexation that can induce satisfactory stereoselectivity in the aluminum‐catalyzed ROP of *rac*‐lactide.

Our group has a long standing interest in N‐heterocyclic carbene (NHC) adducts of main group elements and their use as ligands in transition metal chemistry,^[20]^ and for instance, imidazolin‐2‐iminato and imidazolin‐2‐imine ligands have found numerous applications as ancillary ligands in homogeneous catalysis.^[21]^ More recently, the related heavier congeners of these nitrogen donor ligands, NHC‐phosphinidene adducts of the type (NHC)PR have found significant interest, with numerous applications in main group element and transition metal chemistry.[^20^, ^22^] These systems can be conceived as inversely polarized phosphaalkenes,^[23]^ and the degree of polarization of the phosphorus‐carbon double bond, as monitored by ^31^P NMR spectroscopy, can serve as an indicator of the π‐accepting properties of the corresponding carbene.^[24]^ These features afford strongly nucleophilic P‐donor ligands that might find interesting applications in homogeneous catalysis,^[25]^ in a similar fashion as N‐heterocyclic olefin (NHO) systems of the type (NHC)CH_2_.^[26]^ The related terminal NHC‐phosphinidene adducts (NHC)PH have found surprisingly little attention as potential ancillary ligands, despite the availability of several species with NHC=IDipp,[^27^, ^28^, ^29^] IMes,[^30^, ^31^] SIDipp,^[32]^ SIMes,^[33]^ IMe,^[31]^ ^Me^IMe,^[31]^ I*i*Pr,^[31]^ IAr*^[34]^ (Ar*=2,6‐bis(diphenylmethyl)‐4‐methylphenyl; for NHC acronyms, see ref. [20]). Terminal NHC‐arsinidenes are also known for NHC=IDipp, IMes, IAr*.^[35]^

In our hands, convenient access to (IDipp)PH (**3 a**) was found through the reaction of *N*,*N*’‐1,3‐bis(2,6‐diisopropylphenyl)‐2,2‐difluoroimidazoline (**1 a**, “PhenoFluor”) with P(SiMe_3_)_3_, followed by desilylation of (IDipp)PSiMe_3_ (**2 a**) in methanol (Scheme 2).^[29]^ **2 a** reacted with various metal halides to afford complexes of the type [{(IDipp)P}ML_n_],[^29^, ^36^, ^37^] which display characteristics of related phospinidene transition metal complexes.^[38]^ The coordination chemistry of **3 a** towards group 8 and 9 metals was also studied, providing access to chiral half‐sandwich complexes of the type [η^6^‐*p*‐cymene){(IDipp)PH}MCl_2_] (M=Ru, Os) and [η^5^‐C_5_Me_5_){(IDipp)PH}MCl_2_] (M=Rh, Ir).^[36]^ In addition, the carbonyl complexes [{(IDipp)PH}M(CO)_5_] were prepared to determine the electron‐donating properties of **3 a**, among other things, by IR spectroscopy.^[39]^ [{(IMes)PH}W(CO)_5_] and [{(IAr*)PH}Fe(CO)_4_] represent additional examples,[^30^, ^34^] while the aluminum and gallium complexes [{(SIMes)PH}M*t*Bu_2_Cl] (M=Al, Ga) represent, to the best of our knowledge, the only terminal NHC‐phosphinidene complexes of main group metals.^[33]^

**Scheme 2:**
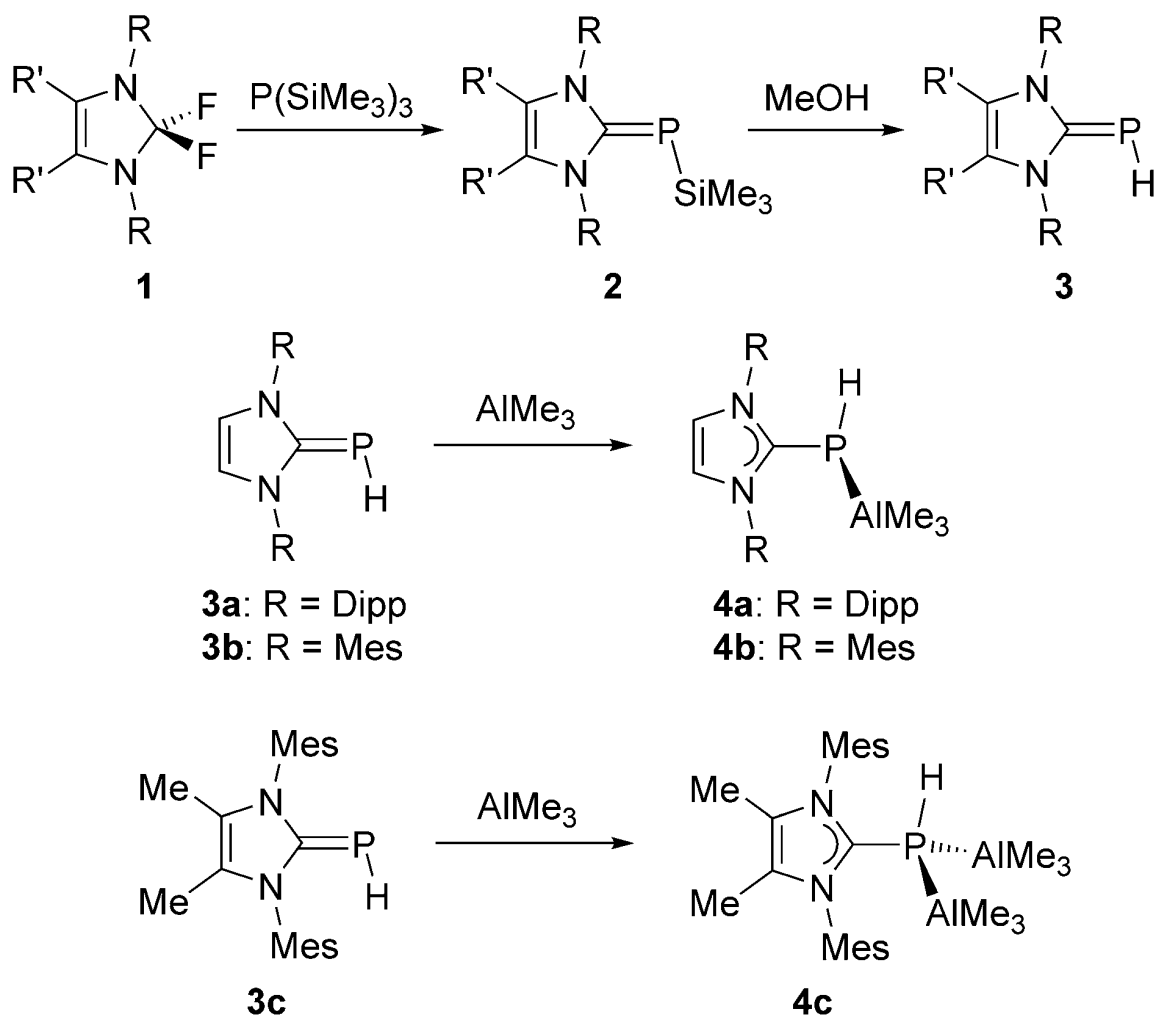
Synthesis of terminal NHC‐phosphinidene adducts and their trimethylaluminum complexes.

In this context, we were interested in the preparation of trimethylaluminum (AlMe_3_) complexes of terminal NHC‐phosphinidene adducts and their use as initiators for the ROP of *rac*‐lactide. The (NHC)PH ligands **3 b** (NHC=IMes) and **3 c**, NHC=^Me^IMes) were synthesized in a similar fashion as previously described for **3 a** (NHC=IDipp).^[29]^ Hence, the difluorides **1 b** and **1 c** were treated with P(SiMe_3_)_3_ in toluene at elevated temperature to furnish the trimethylsilylphosphinidene adducts **2 b** and **2 c** as yellow crystalline solids in high yield (ca. 80 %, Scheme 2). Desilylation of **2 b** and **2 c** can be accomplished in toluene solution in the presence of excess methanol, providing **3 b** and **3 c** in almost quantitative yields as brownish solids. The spectroscopic data of **2 b**/**2 c** and **3 b**/**3 c** agree with those previously reported for **2 a** and **3 a**,^[29]^ and the ^31^P NMR spectra exhibit the expected characteristic high‐field signals (Table 1). The molecular structures of **2 b**, **2 c**, and **3 c** were determined by X‐ray diffraction analysis, revealing structural features similar to those published previously for related systems (Table 1).[^28^, ^29^, ^30^, ^31^]

**Table 1:** Pertinent spectroscopic and structural data of compounds **2**–**4**.

Comp.	*δ* ^31^P [ppm]	^1^ *J*_PH_ [Hz]	C–P [Å]	P–Al [Å]
**2 a**	−129.5 (s)	–	1.7800(13)^[29]^	–
**2 b**	−135.8 (s)	–	1.7701(14)/ 1.7658(14)^[a]^	–
**2 c**	−137.6 (s)	–	1.7717(13)	–
**3 a**	−133.9 (d)	165.1	1.752(1)^[28]^	–
**3 b**	−146.5 (d)	165.4	1.747(2)^[31]^	–
**3 c**	−146.8 (d)	164.0	1.7561(18)/1.7554(19)^[a]^	–
**4 a**	−137.9 (d)	210.6	1.7944(13)	2.4973(5)
**4 b**	−147.4 (d)	208.8	1.7961(6)	2.5173(3)
**4 c**	−142.9 (d)	224.2	1.822(3)	2.5348(12)/2.6104(13)

[a] For two crystallographically independent molecules.

Toluene solutions of the terminal NHC‐phosphinidene adducts **3 a**–**3 c** were treated with equimolar amounts of a trimethylaluminum solution (2.0 m) in toluene at room temperature. In case of **3 a** and **3 b**, the 1:1 adducts [{(NHC)PH}AlMe_3_] (**4 a**, NHC=IDipp; **4 b**, NHC=IMes) readily formed as yellowish crystalline solids in high yield, whereas the 1:1 adduct could not be isolated from **3 c**, since a strong tendency towards the formation of the 1:2 adduct [{(^Me^IMes)PH}(AlMe_3_)_2_] (**4 c**) was observed. Under optimized conditions, however, the dialuminum compound **4 c** can be prepared in high yield by treatment of **3 c** with two equivalents of AlMe_3_. This reactivity is reminiscent of the formation of the bis(borane) adduct [{(IMes)PPh}(BH_3_)_2_] upon reaction of (IMes)PPh with [(THF)BH_3_].^[40]^ All three adducts **4 a**–**4 c** show ^31^P NMR signals that are only slightly shifted compared to **3 a**–**3 c**, with the expected increase of the ^1^ *J*_PH_ coupling constants upon formation of the phosphorus–metal bonds (Table 1).^[41]^ The ^1^H NMR spectra exhibit characteristic low‐field signals at −0.47 ppm (**4 a**), −0.42 ppm (**4 b**), and −0.34 ppm (**4 c**) corresponding to the AlMe_3_ methyl groups together with the expected doublets in the range 2.1–2.8 ppm for the PH hydrogen atoms with ^1^ *J*_PH_>200 Hz.

The molecular structures of **4 a**–**4 c** could be confirmed by X‐ray crystallography (see Figure 1 for an ORTEP presentation of **4 a** and the Supporting Information for all other crystal structures). The Al‐P bonds of 2.4973(5) Å (**4 a**) and 2.5173(3) Å (**4 b**) are just slightly longer than reported for [{(SIMes)PH}Al*t*Bu_2_Cl] (2.483(1) Å),^[33]^ but significantly shorter than found for phosphine‐AlMe_3_ adducts, e.g., 2.535(1) Å in [(Ph_3_P)AlMe_3_] and 2.584(2) Å in [{(2‐MeC_6_H_4_)_3_P}AlMe_3_].^[42]^ The Al atoms reside in distorted tetrahedral environments with the P‐Al‐C angles ranging from 89.02(5)° to 116.77(5)° in **4 a** and from 98.55(3)° to 112.46(3)° in **4 b**, revealing an asymmetric binding mode of the (NHC)PH ligands with Al‐P‐C1 angles of 124.98(4)° in **4 a** and 110.81(2)° in **4 b**. Coordination of the AlMe_3_ moiety affords elongated P−C1 bond lengths in comparison with the (NHC)PH ligands **3 a** and **3 c**, while this elongation is even more pronounced for the bis(trimethylaluminum) complex **4 c** (Table 1). In the latter, the coordination sphere around the phosphorus atom is best described as trigonal‐pyramidal with large C1‐P‐Al1/Al2 and Al1‐P‐Al2 angles of 115.64(11)°, 121.54(11) and 121.54(11)° and the PH hydrogen atom in the apical position. These structural features indicate that the P–Al interaction in complexes **4** involves the carbon‐phosphorus double bond, which is strongly polarized towards the P atom. Accordingly, coordination to one AlMe_3_ unit through either one of the enantiotopic faces of the planar (NHC)PH ligands affords the chiral, *C*_1_‐symmetric aluminum complexes **4 a** and **4 b**, which crystallize as racemic mixtures, whereas *C*_s_‐symmetric **4 c** is formed by coordination of two AlMe_3_ units from both sides. It should be noted, however, that the NMR spectra indicate fast rotation around the carbon‐phosphorus bonds in **4 a**–**4 c** and also fast interconversion of the enantiomers of **4 a** and **4 b** on the NMR time‐scale at room temperature, in agreement with previous variable‐temperature NMR studies of (IDipp)PH transition metal complexes.^[36]^

**Figure 1:**
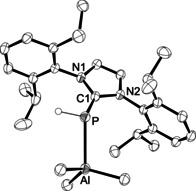
ORTEP diagram of **4 a** with thermal displacement parameters drawn at 50 % probability level; pertinent structural data can be found in Table 1.

Complexes **4 a**–**4 c** were successfully employed as initiators for the ROP of *rac*‐lactide in toluene at 60 °C under the conditions mentioned in Table 2. High conversions (>80 %) were reached for monomer:catalyst ratios of 100–500 within 12 h (**4 a, 4 c**) or 10 h (**4 b**), respectively (Entries 1–15). In case of **4 b**, PLA could also be isolated in high yield (82 %) after 24 h in the presence of 1000 equivalents of the monomer (Entry 16), whereas ROP in THF or CH_2_Cl_2_ solution furnished only low conversions (Entries 17 and 18). All catalysts enabled well‐controlled polymerization, with an excellent agreement of calculated and experimental molar masses *M*_n,calc_ and *M*_n,exp_, a linear increase of *M*_n,exp_ with increasing monomer:catalyst ratio (Figures S41–43), and narrow polydispersities (PDI<1.50). Based on the time required to achieve full conversion of *rac*‐LA into PLA, the order of activity follows **4 b>4 c>4 a**. To quantify these observations, kinetic polymerization studies were performed by ^1^H NMR spectroscopy at 60 °C: 28.8 mg (0.2 mmol) of *rac*‐lactide with 1 mg (0.01 mmol) of tetramethylsilane as an internal standard were dissolved in C_6_D_6_ (1 mL), and the concentration [cat]_0_ of the respective catalyst **4 a**–**4 c** (2–10 mmol L^−1^) was varied. In all cases, a first‐order dependence of the conversion rate with respect to the *rac*‐lactide concentration was observed, as evidenced by the linear fit of data to plots of ln([LA]_0_/[LA]_*t*_) versus time (*t*). The observed rate constants (*k*_obs_) follow the expected order **4 b>4 c>4 a**, and plots of *k*_obs_ versus catalyst concentration indicated that the ROP is also first order in [cat]_0_, affording second‐order rate constants of propagation (*k*_p_) of 20.1±1.2 m ^−1^ h^−1^ (**4 a**), 24.4±1.7 m ^−1^ h^−1^ (**4 b**) and 21.3±0.8 m ^−1^ h^−1^ (**4 c**); see Figures S48‐S55 and Tables S1–S7 for details. These values agree with those reported for the polymerization of *rac*‐LA with salen–aluminum complexes.^[18]^ ROP reactions were also monitored in the temperature range of 65–85 °C with [*rac*‐LA]=0.2 mol L^−1^ and [cat]_0_=4 mmol L^−1^, and activation parameters were obtained from the resulting Eyring plots,^[43]^ that is, **4 a**: Δ*H* ^≠^=57(3) kJ mol^−1^, Δ*S* ^≠^=−123(7) J mol^−1^ K^−1^; **4 b**: Δ*H* ^≠^=47(2) kJ mol^−1^ and Δ*S* ^≠^=−142(6) J mol^−1^ K^−1^; **4 c**: Δ*H* ^≠^=52(3) kJ mol^−1^, Δ*S* ^≠^=−139(1) J mol^−1^ K^−1^ (see Figures S56–S64 and Tables S8–S11 for details). These values closely match with the activation parameters determined for other lactide polymerization catalysts that operate via an associative coordination‐insertion mechanism.[^44^, ^45^]

**Table 2:** Polymerization of *rac*‐lactide in the presence of the AlMe_3_ complexes **4 a**‐**4 c**.^[a]^

Entry	Catalyst	[*rac‐*LA]/[cat.]	Solvent	Time [h]	Conv.^[b]^	*M*_n,calc_^[c]^ [kDa]	*M*_n,exp_^[d]^ [kDa]	PDI^[d]^	*P*_m_^[e]^	*T*_m_^[f]^ [°C]
1	**4 a**	100	toluene	12	94	13.5	13.6	1.35	0.92	191
2	**4 a**	200	toluene	12	92	26.5	27.1	1.31	0.90	189
3	**4 a**	300	toluene	12	91	39.3	40.1	1.27	0.85	187
4	**4 a**	400	toluene	12	89	51.3	52.8	1.41	0.83	182
5	**4 a**	500	toluene	12	82	59.1	59.6	1.33	0.81	178
6	**4 b**	100	toluene	10	97	14.0	13.8	1.29	0.75	172
7	**4 b**	200	toluene	10	95	27.4	27.9	1.31	0.75	170
8	**4 b**	300	toluene	10	96	41.5	42.5	1.46	0.73	168
9	**4 b**	400	toluene	10	94	54.2	54.9	1.28	0.71	162
10	**4 b**	500	toluene	10	89	64.1	65.0	1.65	0.67	158
11	**4 c**	100	toluene	12	96	13.8	13.9	1.28	0.58	–
12	**4 c**	200	toluene	12	92	26.5	26.9	1.31	0.59	–
13	**4 c**	300	toluene	12	91	39.3	40.1	1.30	0.56	–
14	**4 c**	400	toluene	12	90	51.8	51.9	1.42	0.58	–
15	**4 c**	500	toluene	12	88	63.4	63.3	1.35	0.57	–
16	**4 b**	1000	toluene	24	82^[g]^	118.1	119.0	1.67	0.66	–
17	**4 b**	100	THF	24	20	2.9	3.7	–	–	–
18	**4 b**	200	CH_2_Cl_2_	24	25	7.2	5.1	–	–	–

[a] All reactions were carried out at 60 °C by dissolving 100–1000 mg (0.694–6.94 mmol) of *rac*‐lactide and the respective catalyst **4** (6.94 μmol) in the respective solvent (1 mL). [b] Conversions were determined by ^1^H NMR spectroscopy. [c] *M*_n,calc_=molecular weight of chain‐end+144.12 g mol^−1^×[*rac‐*LA]/[cat.]×conversion. [d] The molecular weights were determined with a GPC‐PSS SECcurity system (flow rate=1.0 mL min^−1^) for THF solutions of the polymer (2 mg mL^−1^). Universal calibration was carried out with polystyrene standards and laser light scattering as well as concentration detectors. [e] *P*_m_ is the probability of forming a new *meso*‐diad. [f] Melting temperatures (*T*_m_) were measured by DSC, and the *T*_m_ values were recorded in the second run. [g] Isolated yield.

To examine the stereoregularity of the resulting PLA materials, the probability of *meso* linkages as defined by the parameter *P*_m_ was determined, which can be calculated from the homonuclear decoupled ^1^H NMR spectra by integration of the relative tetrad intensities.^[46]^ All spectra recorded for the PLA samples obtained with **4 a** and **4 b** exhibit a predominant signal in the methine region that can be assigned to the *mmm* tetrad, revealing stereoselective polymerization and formation of PLA with a considerable degree of isotacticity. Catalyst **4 a** shows a high level of stereocontrol (*P*_m_=0.92, Table 1, Entry 1), which decreases upon increasing the concentration of the monomer (*P*_m_=0.90–0.81, Entries 2–5). Figure 2 shows the deconvoluted methine region of the ^1^H NMR spectrum of the PLA from Entry 1, which exhibits signals of small intensity for the *rmm*, *mmr*, and *mrm* tetrads. Together with the negligible presence of the *rmr* signal, these ^1^H NMR characteristics indicate the formation of stereo‐multiblock PLA (Figure 2).[^16^, ^17^] The high isotacticity can also be confirmed by ^13^C{^1^H} NMR spectroscopy (Figures S69, S76).

**Figure 2:**
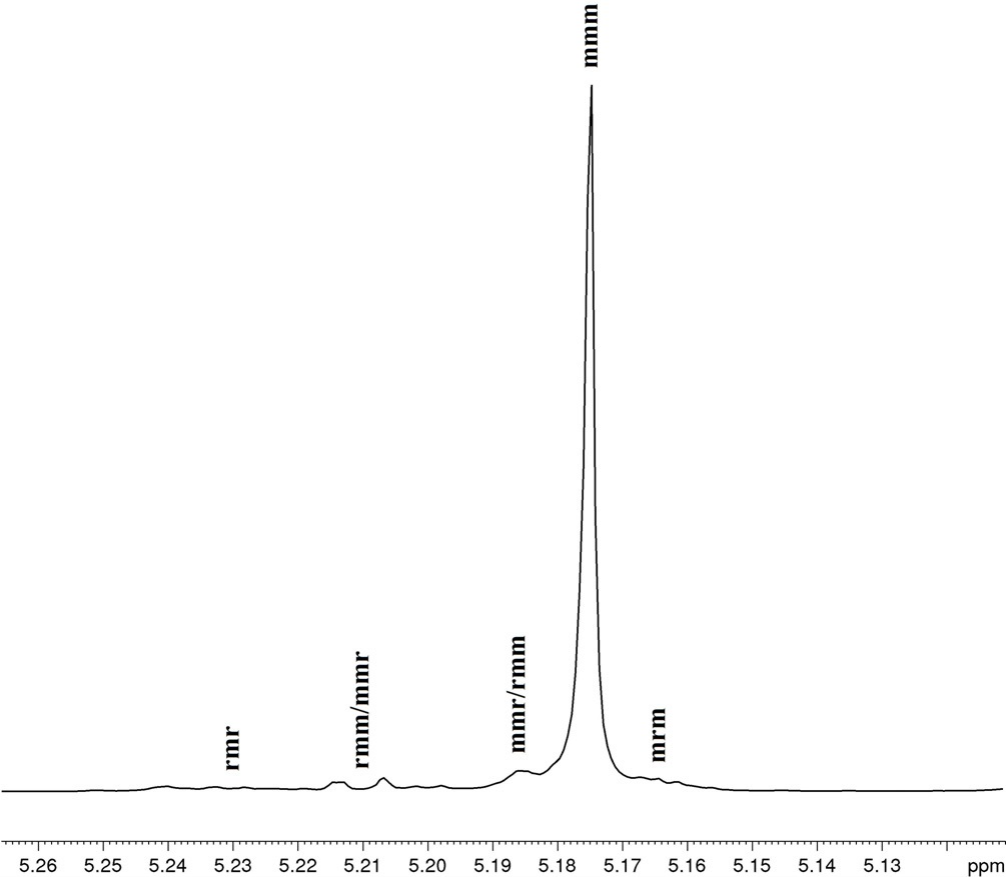
Homonuclear decoupled ^1^H NMR spectrum (500 MHz, CDCl_3_) of the methine region of PLA from Entry 1 in Table 2 (*P*_m_=0.92).

The high isotacticity of the polymers formed in the presence of **4 a** is also revealed by their thermophysical properties (glass transition temperature *T*_g_, melting temperature *T*_m_, melting enthalpy Δ*H_m_*), which were established by means of digital scanning calorimetry (DSC, see Figure S91–S97 and Table S15 in the Supporting Information for full details). High *T*_m_ values in the range 191–178 °C were found (Table 2, Entries 1–5), which correlate linearly with the *P*_m_ values (0.92–0.81) as expected for stereo‐multiblock PLA (Figure S97).^[47]^ Catalyst **4 b** afforded polymers with significantly lower isotacticity compared to **4 a**, and the *P*_m_ values decrease to 0.75–0.67 together with the corresponding *T*_m_ values 172–141 °C (Entries 6–10). These findings suggest that the larger (IDipp)PH ligand in **4 a** induces higher isotacticity, whereas the smaller (IMes)PH ligand in **4 b** gives rise to higher activity at the expense of stereoselectivity. In contrast, the dialuminum complex **4 c** provided PLA with only little stereoregularity, with the *P*_m_ values 0.59–0.52 indicating almost atactic polymers (Entries 11–15). It should be noted that ROP of *rac*‐LA in the presence of AlMe_3_ (in toluene solution) produced completely atactic PLA (*P*_m_≅0.52, Figure S84).

End‐group analysis of the PLA materials by ^1^H NMR spectroscopy revealed that the polymers were terminated exclusively by acetyl (MeCO) and hydroxyl groups (HOCHMe), in agreement with lactide insertion into the Al–Me bonds as the initiation step (Figure S44).[^48^, ^49^] Likewise, polymerization of *rac*‐LA in the presence of isopropanol (5 equivalents) afforded PLA materials with isopropoxycarbonyl [*i*PrOCO] and hydroxyl (HOCHMe) end groups, indicating initiation by an aluminum‐isopropoxide complex (Figures S47).[^19^, ^49^] These findings confirm that the polymerization proceeds via a coordination‐insertion mechanism. Furthermore, heating toluene solutions of **4 a** and **4 b** up to 80 °C for 6 h did not lead to cleavage of the P−H bond (Figures S37–S40), e.g., by methane (CH_4_) elimination as observed for related imidazolin‐2‐imine‐aluminum complexes.^[50]^ These observations indicate that the (NHC)PH ligands are not degraded and are therefore able to sustain a potential stereocontrol during the polymerization process. Accordingly, the high stereoselectivity achieved with catalysts **4 a** and **4 b** can have various reasons: In addition to conventional chain‐end control (CEC), in which the insertion of either LLA or DLA is controlled by the chirality of the previously enchained monomer, the ligand might cooperatively induce enantiomorphic site control (ESC), effecting the stereoselectivity of the catalyst system.[^45^, ^51^] Consequently, the higher *P*_m_ values found for **4 a** compared to **4 b** can be ascribed to the decreasing bulkiness from the (IDipp)PH to the (IMes)PH ligand. Thereby, it should be emphasized that the fluxional coordination of the (NHC)PH ligands through both enantiotopic faces as evidenced by NMR spectroscopy (vide supra) should allow a flexible site control and an adjustment to the chirality imposed mainly by the chain end. The observation of lower *P*_m_ values for higher monomer:catalyst ratios could then tentatively be attributed to a different interplay of the opposing effects, CEC and ESC, which can influence the stereocontrol by generating additional stereo‐errors.

To the best of our knowledge, the complexes **4 a** and **4 b** represent the first highly isoselective aluminum catalysts, which contain comparatively simple monodentate ligands as a stereodirecting group. While N‐heterocyclic carbenes alone^[52]^ and also their metal complexes^[53]^ may act as efficient ROP initiators, the aluminum alkyl complexes (NHC)AlR_3_ (NHC=IDipp, IMes; R=Me, Et) produce atactic PLA, which is formed by lactide insertion into the Al−C_carbene_ bond and chain growth via an imidazolium‐aluminate zwitterion.^[54]^ This reactivity is reminiscent of frustrated NHC‐borane Lewis pairs,^[55]^ which dissociate into the free Lewis acids and bases in solution, leading to cooperative substrate activation. In contrast, ROP catalyzed by **4 a**–**4 c** proceeds by methyl transfer and insertion into the Al–CH_3_ bonds, with the (NHC)PH ligand clearly affecting the overall stereocontrol. These results demonstrate the potential of N‐heterocyclic carbene‐phosphinidene adducts to serve as a novel class of useful prochiral ancillary ligands.

## Conflict of interest

The authors declare no conflict of interest.

## Supporting information

As a service to our authors and readers, this journal provides supporting information supplied by the authors. Such materials are peer reviewed and may be re‐organized for online delivery, but are not copy‐edited or typeset. Technical support issues arising from supporting information (other than missing files) should be addressed to the authors.

Supplementary
