# Supplementary material for: Isoselective Polymerization of rac‐Lactide by Aluminum Complexes of N‐Heterocyclic Carbene‐Phosphinidene Adducts
Source: Chemistry. 2021 Mar 3;27(19):5913–8. doi: 10.1002/chem.202100482 (PMC8048956; doi:10.1002/chem.202100482)
Supplement: Supplementary file 1 — Supplementary [file CHEM-27-5913-s001.pdf]

Chemistry–A European Journal

Supporting Information

Isoselective Polymerization of *rac*-Lactide by Aluminum Complexes of N-Heterocyclic Carbene-Phosphinidene Adducts

Jayeeta Bhattacharjee,* Marius Peters, Dirk Bockfeld, and Matthias Tamm*[a]

Table of Contents

(S1) Complete experimental procedures of all new compounds 2
(S2) NMR and other spectra 6
(S3) Investigation of the thermal stability of complexes **4a** and **4b** by NMR spectroscopy 25
(S4) Experimental procedure for polymerization 28
(S5) Kinetic studies 34
(S6) Thermodynamic Parameters 45
(S7) PLAs: Stereochemistry and Microstructure 53
(S8) Characterization of polymers 74
(S9) Single crystal X-ray structural data of compounds 82
(S10) References 95

(S1) Complete experimental procedures of all new compounds

General methods and materials

All manipulations were carried out using standard Schlenk line or Glove box (MBraun 200B) techniques. Commercial grade solvents (*n*-hexane, toluene, DCM and THF) used in this study were dried over MBraun Gmbh solvent purification system, and their water content was checked using the Karl-Fischer titration technique (<7ppm). Methanol was refluxed over Mg turnings and distilled. NMR solvents such as C_6D_6, toluene-d_8 were distilled over NaK alloy and $CDCl_3$ were distilled over CaH_2 subsequently degassed and stored over molecular sieves (3 or 4 Å) in the glove box.

Materials and analytical methods

The starting materials **1a**–**3a**,[1] [IMesCl]Cl (1,3-bis(2,4,6-trimethylphenyl)-2-chloroimidazolium chloride),[2] **1b**,[3] (^{Me}IMesH)Cl,[4] were prepared according to previously published procedures. ^{1}H, ^{13}C and ^{31}P NMR spectra were measured on the spectrometers Bruker AV 300 (300 MHz), Bruker DRX 400 (400 MHz), Bruker AV III-400 (400 MHz), Bruker AV III-HD500 (500 MHz), and Bruker AV II-600 (600 MHz) spectrometers. If required, the assignment of signals was supported by 2D experiments (^{1}H–^{1}H COSY, ^{1}H–^{13}C HSQC, ^{1}H–^{13}C HMBC). The chemical shifts are given in parts per million (δ; ppm) relative to residual solvent peaks (δ; 7.15 (C_6D_6), 7.26 ($CDCl_3$), 2.09 (toluene–d_8). Coupling constants (J) are reported in Hertz (Hz), and splitting patterns are indicated as s (*singlet*), d (*doublet*), t (*triplet*), m (*multiplet*), sept (*septet*) and br (*broad*). All the spectra were measured at room temperature unless otherwise stated. Elemental analyses of all the complexes were carried out with a Vario Micro Cube System (Elementar Analysensysteme GmbH).

Crystallography

A single crystal of **3c** was mounted on a MiTeGen mount with per-fluorinated inert oil. All other crystals were mounted on top of a human hair in per-fluorinated inert oil. Intensity measurements were performed at 100 K on a Rigaku XtaLAB Synergy S diffractometer equipped with a PhotonJet microfocus source and a HyPix-6000HE detector using mirror fucossed Cu$K\alpha$ (**2b**, **4a**, **4c**) and Mo$K\alpha$ (**4b**) radiation or Oxford Diffraction Xcalibur Nova diffractometers using mirror-focussed Cu$K\alpha$ radiation (**1c**, **2c**, **3c**). Data reduction was performed with CrysalisPro.[5] Absorption correction was based on multi-scans and for **2b**, **4b** and **4c** additionally face indexation and integration on a Gaussian grid was applied. The structures were solved by intrinsic phasing with SHELXT 2014/5 (**1c**, **2c**, **3c**) and 2018/2 (**2b**, **4a**, **4b**, **4c**)[6] and refined on F2 using the program SHELXL 2017/1 (**1c**, **2c**, **3c**) and 2018/3 (**2b**, **4a**, **4b**, **4c**) [7] in OLEX2.[8] H atoms unless otherwise noted were placed in idealized positions and refined using a riding model. Complete data have been deposited at the Cambridge Crystallographic Data Centre under the numbers CCDC 2054478 (**1c**), 2054479 (**2b**), 2054480 (**2c**), 2054481 (**3c**), 2054482 (**4a**), 2054483 (**4b**) and 2054484 (**4c**). These data can be obtained free of charge from www.ccdc.cam.ac.uk/data_request/cif.

Synthesis of (IMes)F_2 (1b)

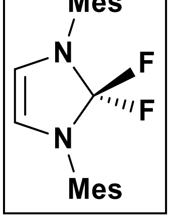

[IMesCl]Cl was finely ground using a mortar and dried under vacuum at 70 °C for 5 h. To a Schlenk tube containing [IMesCl]Cl (3.05 g, 8.13 mmol) and CsF (12.35 g, 81.30 mmol) was added toluene (200 mL). The Schlenk tube was sonicated for 30 min and then stirred vigorously at 90 °C for 4 days. Formation of a brown solution was observed during the reaction. The reaction mixture was brought to room temperature, filtered through a pad of Celite and the residue washed with toluene (3 x 20 mL). The filtrate was concentrated and dried under vacuum, washed with cold *n*-hexane followed by cold CH_3CN (–10 °C) and dried under vacuum to afford **1b** as a pale brown solid.

Yield: 1.93 g (68.9%). ^{1}H NMR (C_6D_6, 300.1 MHz): δ = 6.74 (br s, 4H, *m*-Ar-*H*), 5.43 (s, 2H, *H*-C=C), 2.41 (s, 12, *o*-C*H*$_3$), 2.07 (s, 6H, *p*-C*H*$_3$) ppm. ^{13}C {^{1}H} NMR (C_6D_6, 75.5 MHz): δ = 140.6 (N*C*$_{Mes}$), 138.9 (*o*-*C*$_{Mes}$), 133.2 (*p*-*C*$_{Mes}$), 130.2 (*m*-*C*$_{Mes}$), 112.1 (N*C*H), 21.6 (*p*-CH$_3$), 19.2 (*o*-CH$_3$) ppm. ^{19}F {^{1}H} NMR (C_6D_6, 470.8 MHz): δ = –34.8 ppm. Elemental analysis (%): calcd for $C_{23}H_{28}F_2N_2$ (403.39): C 74.56, H 7.62, N 7.56; found: C 74.91, H 7.79, N 7.44.

Synthesis of (IMes)PSiMe$_3$ (2b)

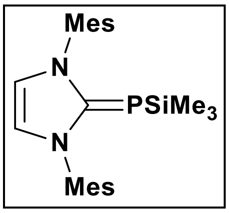

To a stirred solution of (IMes)F_2 (0.504 g, 1.484 mmol) in toluene (10 mL), P(SiMe$_3$)$_3$ (0.377 g, 1.505 mmol) was added drop wise. The resulting pale yellow solution was then heated to 90 °C over 6h, then cooled down to room temperature, and volatiles were removed *in vacuo*. The residue was extracted with *n*-hexane (3 x 5 mL) until the extracts were almost colourless. Evaporation of the solvent under vacuum yielded an almost analytically pure pale brown solid. **2b** can be isolated as a brown crystalline solid of better purity by re-crystallization from a toluene solution at –30 °C.

Yield: 0.498 g (83%). ^{1}H NMR (C_6D_6, 300 .3 MHz): δ = 6.76 (br, 4H, *m*-Ar-*H*), 5.80 (s, 2H, *H*-C=C), 2.20 (s, 12H, *o*-C*H*$_3$), 2.09 (s, 6H, *p*-C*H*$_3$), 0.09 (d, 9H, $^{3}J_{(P, H)}$ = 5.5 Hz, P-Si*Me*$_3$) ppm. ^{13}C {^{1}H} NMR (C_6D_6, 125.8 MHz): δ = 170.5 (d, $^{1}J_{(P, C)}$ = 110.3 Hz, P*C*$_{Carbene}$), 138.9 (N*C*$_{Mes}$), 136.4 (d, $^{4}J_{PC}$ = 1.4 Hz, *o*-*C*$_{Mes}$,), 135.2 (*p*-*C*$_{Mes}$), 129.8 (*m*-*C*$_{Mes}$), 118.9 (d, $^{3}J_{(P, C)}$ = 2.9 Hz, N*C*H), 21.1 (*p*-*C*H$_3$), 18.6 (d, $^{5}J_{(P, C)}$ = 2.7 Hz, *o*-*C*H$_3$), 4.7 (d, $^{2}J_{(P, C)}$ = 18.5 Hz, PSi(*C*H$_3$)$_3$) ppm. ^{29}Si NMR (C_6D_6, 79.5 MHz): δ = –5.51 (d, $^{1}J_{(P, Si)}$ = 70.2 Hz) ppm. ^{31}P NMR (C_6D_6, 202.5 MHz): δ = –135.8 (s) ppm. Elemental analysis (%): calcd for $C_{24}H_{33}N_2PSi$ (408.59): C 70.55, H 8.14, N 6.86; found: C 70.48, H 7.95, N 7.05.

Synthesis of (IMes)PH (3b)

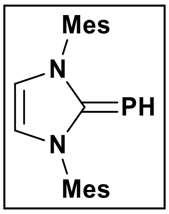

To a stirred solution of (IMes)PSiMe$_3$ (0.273 g, 0.668 mmol) in toluene (10 mL), excess dry CH_3OH (1.3 mL) was added at room temperature. The resulting brown solution was stirred at 60 °C for 4h followed by stirring at room temperature overnight, during which the color changed from dark brown to light brown. All volatiles were then removed under vacuum washed with cold *n*-hexane (2 x 5 mL) and dried to afford compound **3b** as pale brown powder.

Yield: 0.205 g (91%). ^{1}H NMR (C_6D_6, 300.3 MHz): δ = 6.76 (br, 4H, *m*-Ar-*H*), 5.92 (s, 2H, *H*-C=C), 2.21 (s, 12H, *o*-C*H*$_3$), 2.08 (s, 6H, *p*-C*H*$_3$), 2.07 (d, 1H, $^{1}J_{(P, H)}$ = 165.1 Hz, P*H*) ppm. ^{13}C {^{1}H} NMR (C_6D_6, 125.8 MHz): δ = 176.2 (d, $^{1}J_{(P, C)}$ = 85.9 Hz, P*C*$_{Carbene}$), 138.8 (N*C*$_{Mes}$), 136.4 (*o*-*C*$_{Mes}$), 135.3 (*p*-*C*$_{Mes}$), 129.7 (*m*-*C*$_{Mes}$), 117.6 (s, NCH), 21.1 (*p*-*C*H$_3$), 18.2 (d, $^{5}J_{(P, C)}$ = 2.1 Hz, *o*-*C*H$_3$) ppm. ^{31}P NMR (C_6D_6, 121.5 MHz): δ = –146.5 (d, $^{1}J_{(P, H)}$ = 165.4 Hz, P*H*) ppm Elemental analysis (%): calcd for $C_{21}H_{25}N_2P$ (336.42): C 74.98, H 7.49, N 8.33; found: C 75.18, H 7.17, N 8.15.

Synthesis of [^{Me}IMes Cl]Cl

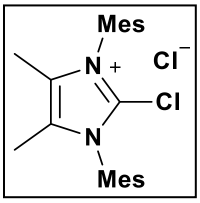

To a Schlenk tube containing (^{Me}IMes) (0.997 g, 2.99 mmol) was added THF(100 mL).The reaction mixture was cooled to –40 °C and 1,1,1,2,2,2-hexachloroethane(0.778 g, 3.30 mmol) was added. The reaction mixture was warmed to 23 °C and stirred at this temperature for 24 h. The reaction mixture was cooled to –10 °C and filtered. The filter cake was washed with THF (3 x 20 mL) and toluene (2 x 20 mL) and dried under reduced pressure to afford [^{Me}IMesCl]Cl as colorless solid.

Yield: 0.992 g (82%).^{1}H NMR (CD_2Cl_2, 500.3 MHz): δ = 7.18 (s, 4H, *m*-Ar-*H*), 2.41 (s, 6H, *p*-C*H*$_3$), 2.20 (s, 6H, NCC*H*$_3$), 2.07 (s, 12H, *o*-C*H*$_3$) ppm. ^{13}C {^{1}H} NMR (CD_2Cl_2, 125.8 MHz): δ = 143.2 (N*C*$_{Mes}$), 134.9 (*o*-*C*$_{Mes}$), 130.8 (*m*-*C*$_{Mes}$), 130.3 (N*C*CH$_3$), 127.5 (*p*-*C*$_{Mes}$), 21.4 (*p*-*C*H$_3$), 17.5 (*o*-*C*H$_3$), 9.9 (NC*C*H$_3$) ppm. Elemental analysis (%): calcd for $C_{23}H_{28}Cl_2N_2$ (403.39): C 68.48, H 7.00, N 6.94; found: C 68.58, H 6.78, N 6.74.

Synthesis of (^{Me}IMes)F_2 (1c)

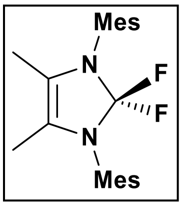

[^{Me}IMes Cl]Cl was finely ground using a mortar and dried under vacuum at 70 °C for 5 h. To a Schlenk tube containing [^{Me}IMes Cl]Cl (3.39 g, 8.40 mmol) and CsF (12.77 g, 84.00 mmol) was added toluene (200 mL). The Schlenk tube was sonicated for 30 min and then stirred vigorously at 100 °C for 4 days. Formation of a dark brown solution was observed during the reaction. The reaction mixture was brought to room temperature, filtered through a pad of Celite and the residue washed with toluene (3 x 20 mL). The filtrate was concentrated and dried under vacuum, washed with cold *n*-hexane followed by cold CH_3CN (–10 °C) and dried under vacuum to afford **1b** as a pale brown solid.

Yield: 2.81 g (90%). ^{1}H NMR (C_6D_6, 500.3 MHz): δ = 6.78 (br s, 4H, *m*-Ar-*H*), 2.41 (s, 12, *o*-CH_3), 2.11 (s, 6H, *p*-CH_3), 1.48 (s, 6H, NCCH_3) ppm. ^{13}C {^{1}H} NMR (C_6D_6, 125.8 MHz): δ = 140.6 (NC_{Mes}), 138.1 (*o*-C_{Mes}), 131.1 (*p*-C_{Mes}), 129.5 (*m*-C_{Mes}), 112.8 (NCCH$_3$), 21.0 (*p*-CH_3), 18.7 (*o*-CH_3), 9.0 (NCCH$_3$) ppm. ^{19}F {^{1}H} NMR (C_6D_6, 470.8 MHz): δ = –36.0 ppm. Elemental analysis (%): calcd for $C_{23}H_{28}F_2N_2$ (403.39): C 74.56, H 7.62, N 7.56; found: C 74.91, H 7.79, N 7.44.

Synthesis of (^{Me}IMes)PSiMe$_3$(2c)

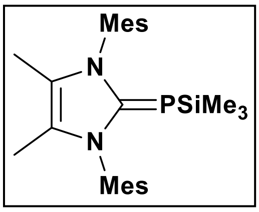

To a stirred solution of (^{Me}IMes)F_2 (2.80 g, 7.56 mmol) in toluene (20 mL), P(SiMe$_3$)$_3$ (1.90 g, 7.56 mmol) was added drop wise. The resulting pale brown solution was then heated to 70 °C over 6h, then cooled down to room temperature, and volatiles were removed *in vacuo*. The residue was extracted with *n*-hexane (3 x 10 mL) until the extracts were almost colorless. Evaporation of the solvent under vacuum yielded an almost analytically pure compound. **2c** can be isolated as a white crystalline solid of better purity from an toluene solution at –30 °C.

Yield 2.62 g (79%). ^{1}H NMR (C_6D_6, 500.3 MHz): δ = 6.80 (m, 4H, *m*-Ar-*H*), 2.23 (s, 12H, *o*-CH_3), 2.12 (s, 6H, *p*-CH_3), 1.37 (s, 6H, NCCH_3), 0.13 (d, 9H, $^3J_{(P, H)}$ = 6 Hz, P-SiMe_3) ppm. ^{13}C {^{1}H} NMR (C_6D_6, 125.8 MHz): δ = 168.8 (d, $^1J_{(P, C)}$ = 110 Hz, P$C_{Carbene}$), 138.8 (*p*-C_{Mes}), 136.9 (d, $^3J_{(P, C)}$ = 1.7 Hz, NC_{Mes}), 133.7 (*o*-C_{Mes}), 129.9 (*m*-C_{Mes}), 121.6 (d, $^3J_{(P, C)}$ = 3 Hz, NCCH$_3$), 21.1 (*p*-CH$_3$), 18.4 (*o*-CH$_3$), 9.3 (NCCH$_3$), 4.7 (d, $^2J_{(P, C)}$ = 19 Hz, PSi(CH$_3$)$_3$), ppm. ^{29}Si NMR (C_6D_6, 99.4 MHz): δ = –6.47 ppm (derived from a ^{1}H,^{29}Si-HMBC NMR experiment). ^{31}P NMR (C_6D_6, 202.5 MHz): δ = –137.6 (s, $^1J_{(P, Si)}$ = 75 Hz) ppm. Elemental analysis (%): calcd for $C_{26}H_{37}N_2PSi$ (436.65): C 71.52, H 8.54, N 6.42; found: C 71.85, H 8.79, N 6.66.

Synthesis of (^{Me}IMes)PH (3c)

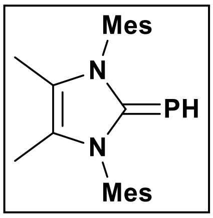

To a stirred solution of (^{Me}IMes)PSiMe$_3$ (1.0 g, 2.29 mmol) in toluene (15 mL), excess dry CH_3OH (1.67 mL, 41.2 mmol, 18 equiv.) was added at room temperature. The resulting solution was stirred overnight, during which the colour changed from brown to almost colorless. All volatiles were then removed under vacuum washed with cold *n*-hexane (2 x 5 mL) and dried to afford compound **3c** as colorless solid.

Yield: 0.637 g (76%). ^{1}H NMR (C_6D_6, 500.3 MHz): δ = 6.80 (s, 4H, Ar-*H*), 2.26 (s, 12H, *o*-CH_3), 2.11 (s, 6H, *p*-CH_3), 2.10 (d, 1H, $^1J_{(P, H)}$ = 164 Hz, P*H*), 1.48 (s, 6H, NCCH_3) ppm. ^{13}C {^{1}H} NMR (C_6D_6, 125.8 MHz): δ = 174.8 (d, $^1J_{(P, C)}$ = 84 Hz, P$C_{Carbene}$), 138.7 (*p*-C_{Mes}), 136.9 (NC_{Mes}), 133.2 (*o*-C_{Mes}), 129.9 (*m*--C_{Mes}), 120.6 (d, $^3J_{(P, C)}$ = 3 Hz, NCCH$_3$), 21.1 (*p*-CH$_3$), 17.9 (d, $^5J_{(P, C)}$ = 2 Hz, *o*-CH$_3$), 9.0 (NCCH$_3$), ppm. ^{31}P NMR (C_6D_6, 202.5 MHz): δ = –146.8 (d, $^1J_{(P, H)}$ = 164 Hz, P*H*) ppm Elemental analysis (%): calcd for $C_{23}H_{29}N_2P$ (364.47): C 75.80, H 8.02, N 7.69; found: C 75.58, H 7.74, N 7.57.

Synthesis of [{(IDipp)PH}AlMe$_3$] (4a)

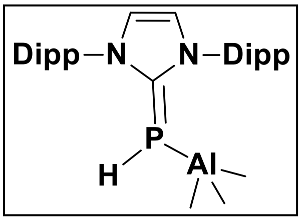

To a stirred yellow suspension of (IDipp)PH (0.20 g, 0.475 mmol) in toluene (10 mL) AlMe$_3$ (0.24 mL of a 2 M solution in toluene, 0.475 mmol) was added slowly at room temperature. The reaction mixture was then stirred overnight. The solvent was removed in vacuo, and the white residue was washed several times with cold *n*-hexane (3 x 5 mL). A white solid was isolated by drying under high vacuum to yield **4a**.

Yield: 0.196 g (84%). ^{1}H NMR (500.3 MHz, C_6D_6) δ = 7.20–7.18 (m, 2H, *p*-Ar-*H*), 7.08 (d, 4H, $^3J_{(H, H)}$ = 7.8 Hz, *m*-Ar-*H*), 6.24 (s, 2H, *H*-C=C), 2.76 (sept, 4H, $^3J_{(H, H)}$ = 6.8 Hz, C*H*(CH$_3$)$_2$), 2.42 (d, 1H, $^1J_{(P, H)}$ =210.4 Hz, P-*H*), 1.40 (d, 12H, $^3J_{(H, H)}$ = 6.8 Hz, CH(C*H$_3$*)$_2$), 0.97 (d, 12H, $^3J_{(H, H)}$ = 6.9 Hz, CH(C*H$_3$*)$_2$), –0.47 (s, 9H, *AlMe$_3$*) ppm. ^{13}C {^{1}H} NMR (C_6D_6, 125.8 MHz): δ = 171.7 (d, $^1J_{(P, C)}$ = 73.5 Hz, PC$_{Carbene}$), 146.1 (N-*C*$_{Dipp}$), 133.2 (*m*-*C*$_{Dipp}$), 131.4 (*o*-*C*$_{Dipp}$), 125.2 (*p*-*C*$_{Dipp}$), 122.5 (d, $^3J_{(P, C)}$ = 2.7 Hz, N*C*H), 29.0 (*C*H(CH$_3$)$_2$), 25.3 (Al*Me$_3$*), 23.2 (CH(*CH$_3$*)$_2$), 23.1 (CH(*CH$_3$*)$_2$) ppm. ^{31}P NMR (C_6D_6, 121.5 MHz): *δ* = –137.9 (d, $J_{(P, H)}$ = 210.6 Hz) ppm. Elemental analysis (%): calcd for $C_{30}H_{46}AlN_2P$ (492.67): C 73.14, H 9.41, N 5.62; found: C 72.98, H 9.47, N 5.22.

X-ray quality crystals were isolated by recrystallizing **4a** from a minimum toluene solution at –30 °C.

Synthesis of [{(IMes)PH}AlMe$_3$] (4b)

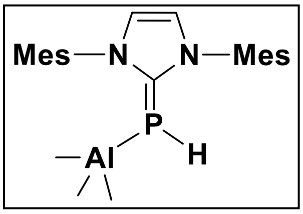

To a stirred brown suspension of (IMes)PH (0.10 g, 0.297 mmol) in toluene (10 mL) AlMe$_3$ (0.15 mL of a 2 M solution in toluene, 0.297 mmol) was added slowly at room temperature. The reaction mixture was then stirred overnight. The solvent was removed *in vacuo*, and the light brown residue was washed several times with cold *n*-hexane (3 x 5 mL). A white solid was isolated by drying under high vacuum to yield **4b**.

Yield: 0.105 g (87%). ^{1}H NMR (C_6D_6, 500.3 MHz): *δ* = 6.72–6.71 (m, 4H, *m*-Ar-*H*), 5.79 (s, 2H, *H*-C=C), 2.41 (d, 1H, $^1J_{(P, H)}$ = 208.6 Hz, P*H*), 2.05 (s, 12H, *o*-C*H*$_3$), 2.04 (s, 6H, *p*-C*H*$_3$), -0.42 (s, 9H, *AlMe$_3$*) ppm. ^{13}C {^{1}H} NMR (C_6D_6, 125.8 MHz): *δ* = 168.3 (d, $^1J_{(P, C)}$ = 81 Hz, PC$_{Carbene}$), 140.2 (N-*C*$_{Mes}$), 139.5 (*o*-*C*$_{Mes}$), 135.3 (*p*-*C*$_{Mes}$), 130.1 (*m*-*C*$_{Mes}$), 122.6 (N*C*H), 21.1 (d, $^5J_{(P, C)}$ = 2.1 Hz, *p*-*C*H$_3$), 18.1 (d, $^5J_{(P, C)}$ = 2.4 Hz, *o*-*C*H$_3$), 17.6 (Al*Me$_3$*) ppm. ^{31}P NMR (C_6D_6, 121.5 MHz): *δ* = –147.4 (d, $J_{(P, H)}$ = 208.8 Hz) ppm Elemental analysis (%): calcd for $C_{24}H_{34}AlN_2P$ (408.51): C 70.57, H 8.39, N 6.86; found: C 70.71, H 8.25, N 6.76.

X-ray quality crystals were isolated by recrystallizing **4b** from a minimum toluene solution at room temperature.

Synthesis of [{(^{Me}IMes)PH}(AlMe$_3$)$_2$] (4c)

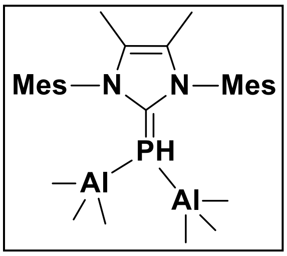

To a stirred brown suspension of (^{Me}IMes)PH (0.20 g, 0.549 mmol) in toluene (10 mL) AlMe$_3$ (0.55 mL of a 2 M solution in toluene, 1.1 mmol) (1.1 mmol) was added slowly at room temperature. The reaction mixture was then stirred overnight. The solvent was removed *in vacuo*, and the brown residue was washed several times with cold *n*-hexane (3 x 5 mL). A light brown solid was isolated by drying under high vacuum to yield **4c**.

Yield: 0.452 g (81%). ^{1}H NMR (C_6D_6, 500.3 MHz): *δ* = 6.75 (m, 4H, *m*-Ar-*H*), 2.72 (d, 1H, $^1J_{(P, H)}$ = 230.2 Hz, P*H*), 2.05 (s, 12H, *o*-C*H*$_3$), 2.04 (s, 6H, *p*-C*H*$_3$), 1.28 (s, 6H, NCC*H*$_3$), -0.36 (s, 18H, *AlMe$_3$*) ppm. ^{13}C {^{1}H} NMR (C_6D_6, 125.8 MHz): *δ* = 160.8 (d, $^1J_{(P, C)}$ = 59 Hz, PC$_{Carbene}$), 140.6 (*p*-*C*$_{Mes}$), 135.5 (N*C*$_{Mes}$), 131.0 (*o*-*C*$_{Mes}$), 130.3 (*m*-*C*$_{Mes}$), 126.2 (d, $^3J_{(P, C)}$ = 2 Hz, N*C*CH$_3$), 21.1 (*p*-*C*H$_3$), 18.2 (Al*Me$_3$*), 17.8 (*o*-*C*H$_3$), 17.3 (Al*Me$_3$*), 8.7 (NC*C*H$_3$), ppm. ^{31}P NMR (C_6D_6, 202.5 MHz): *δ* = –142.0 (d, $J_{(P, H)}$ = 230.1 Hz) ppm. Elemental analysis (%): calcd for $C_{29}H_{47}Al_2N_2P$ (508.64): C 68.48, H 9.31, N 5.51; found: C 67.83, H 9.11, N 5.52.

X-ray quality crystals were isolated by recrystallizing **4c** from a minimum toluene solution at room temperature.

Ring-opening polymerization of *rac*-lactide with addition of alcohol

Formation of metal alkoxide

In a teflon sealed schlenk tube, a toluene-d_8 solution of complex **4b** (0.05 g, 0.122 mmol) was treated with five equiv. of isopropanol(0.037 g, 0.611 mmol) and then warmed to 70 °C temperature. The reaction mixture was then monitored over time. Signals assignable to the carbene-phosphinidene moities were detected; besides, one multiple signals at 4.42-4.34 ppm and one doublet signals at 1.28 ppm appeared. The singlet accounting for Al–CH_3 in **4b** slowly disappears with reduced integral. The integration ratio of product appeared to be 1:3:6 (carbene-phosphinidene moieties:CH:CH_3). Clearly, the alcoholysis reaction afforded the alkyl elimination product [{(IMes)PH}Al{OCH(CH_3)$_2$}$_3$](Figure S45).

Polymerization reaction

A toluene solution of **4b** was treated with five equiv. of isopropanol and used directly to initiate the ring-opening polymerization of *rac*-lactide at 60 ◦C. Rapid polymerization was observed and monomer conversion up to 99% could be reached within 7 h only. The ^{1}H NMR spectrum of the obtained polylactide sample showed that the polymer chains were end-capped with isopropyl ester and a hydroxyl group, respectively (Figure S47).

(S2) NMR and other spectra

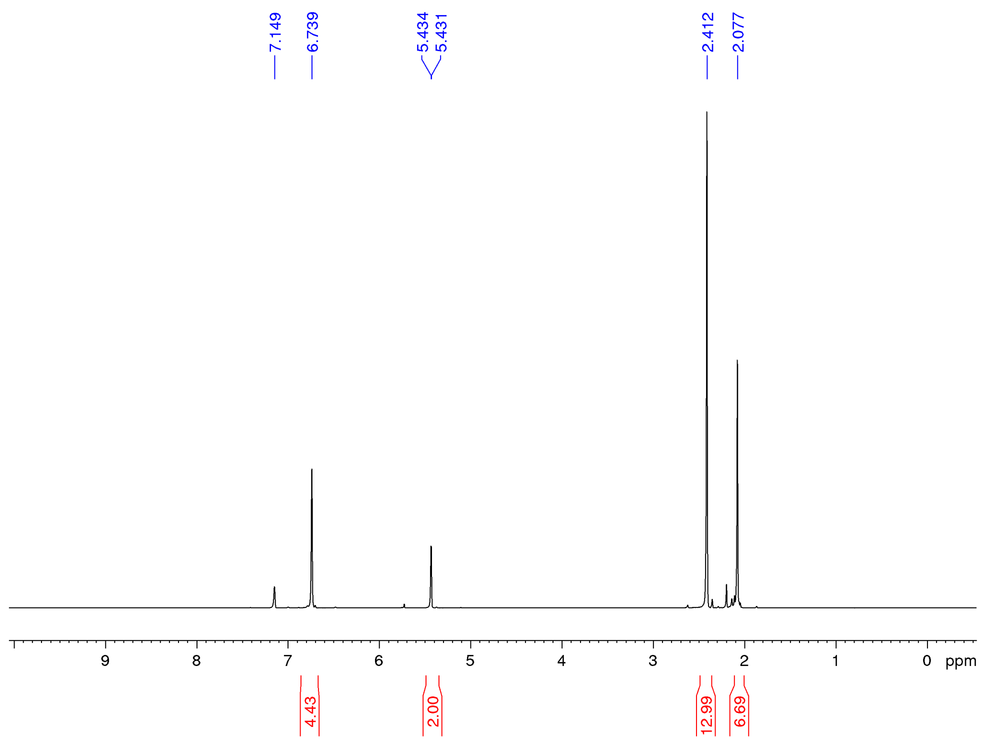

Figure S1. ^{1}H NMR spectrum of (IMes)F_2 (**1b**) in C_6D_6 at room temperature.

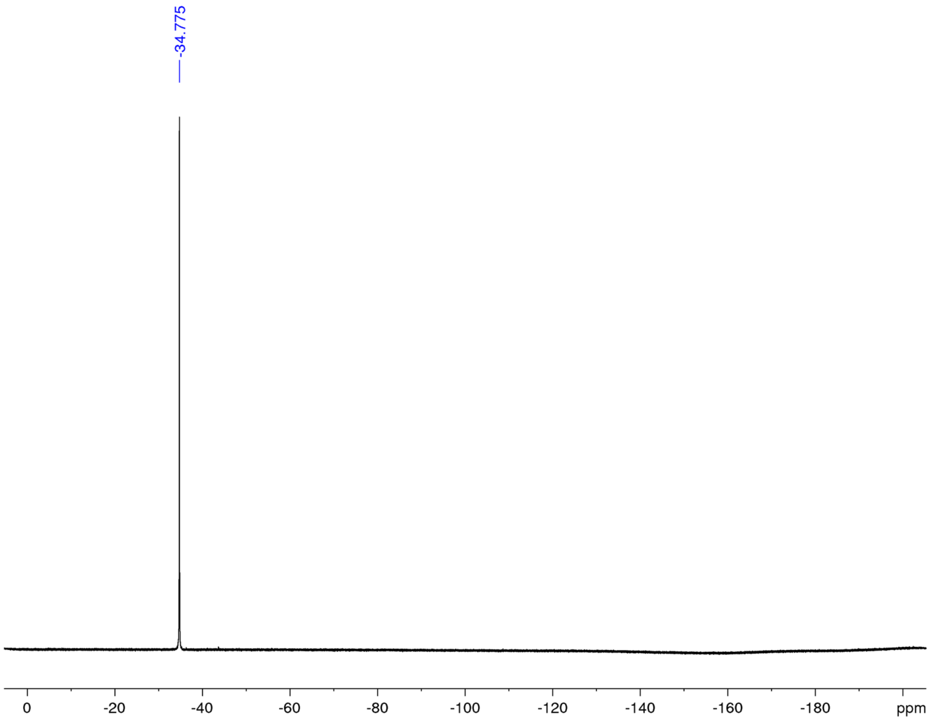

Figure S2. ^{19}F NMR spectrum of (IMes)F_2 (**1b**) in C_6D_6 at room temperature.

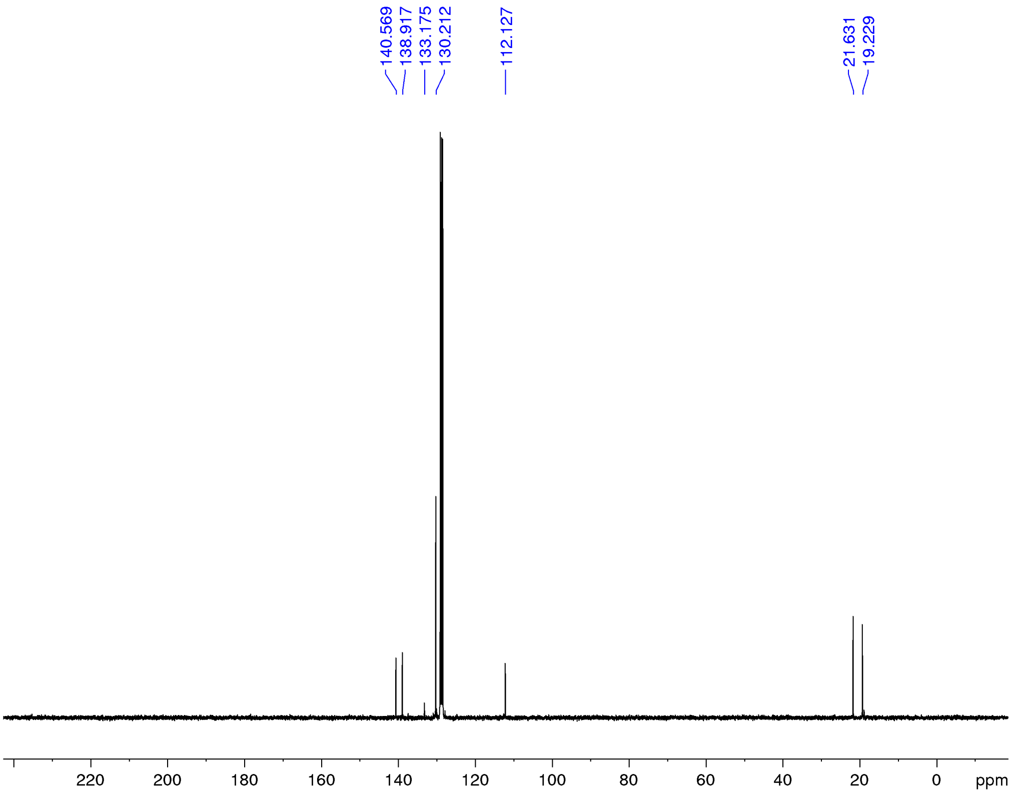

Figure S3. ^{1}H NMR spectrum of (IMes)F_2 (**1b**) in C_6D_6 at room temperature.

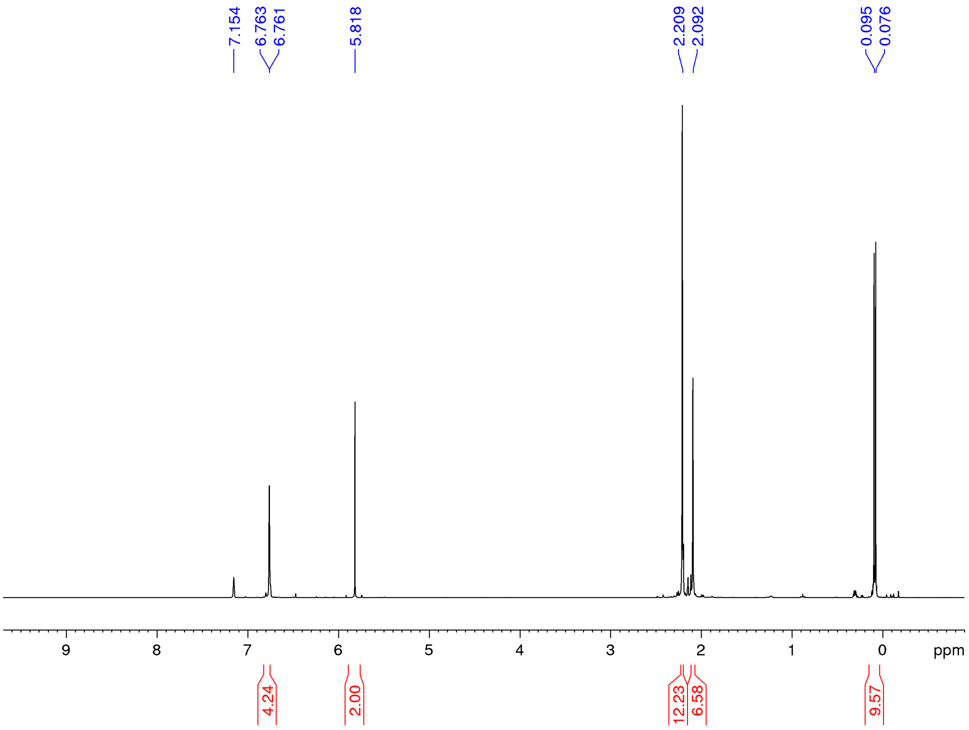

Figure S4. ^{1}H NMR spectrum of (IMes)PSiMe$_3$ (**2b**) in C_6D_6 at room temperature.

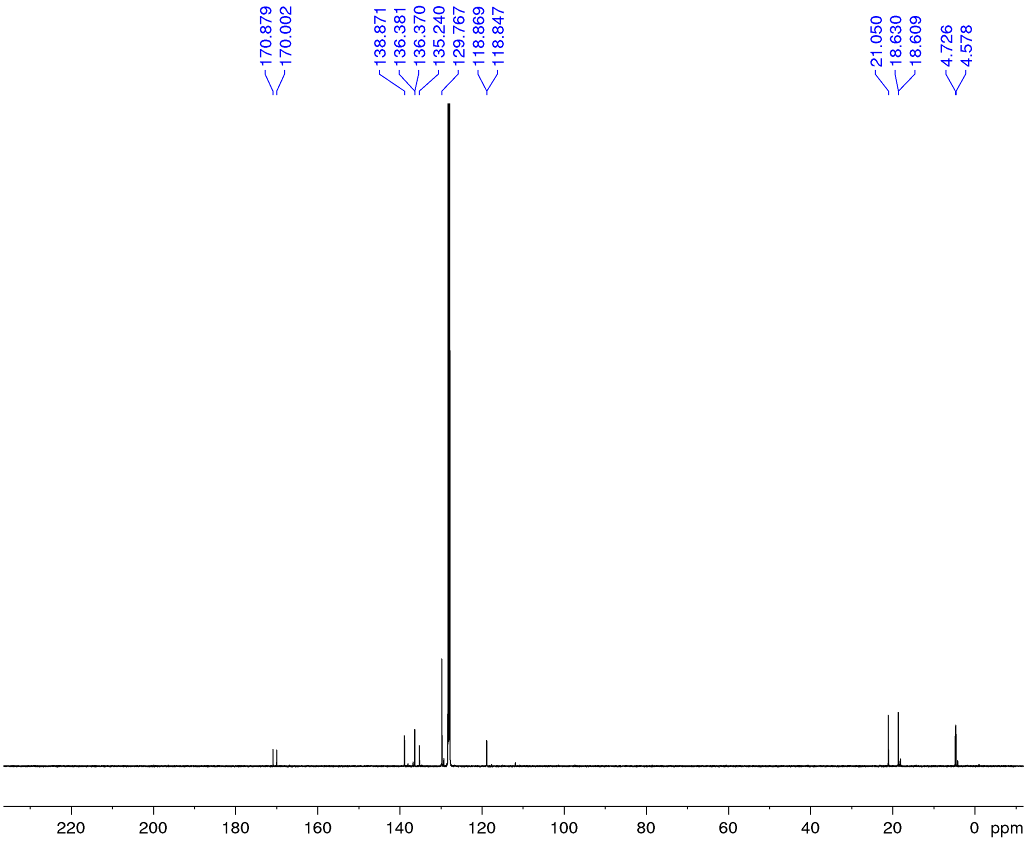

Figure S5. ^{13}C NMR spectrum of (IMes)PSiMe$_3$ (**2b**) in C_6D_6 at room temperature.

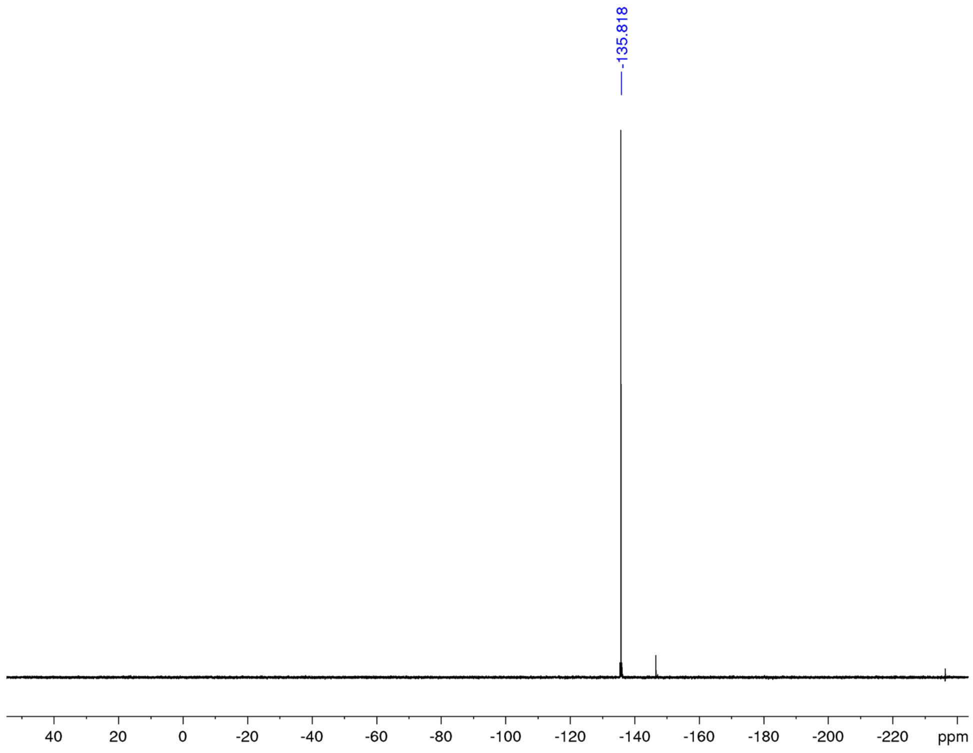

Figure S6. $^{31}P\{^1H\}$ NMR spectrum of (IMes)PSiMe$_3$ (**2b**) in C_6D_6 at room temperature.

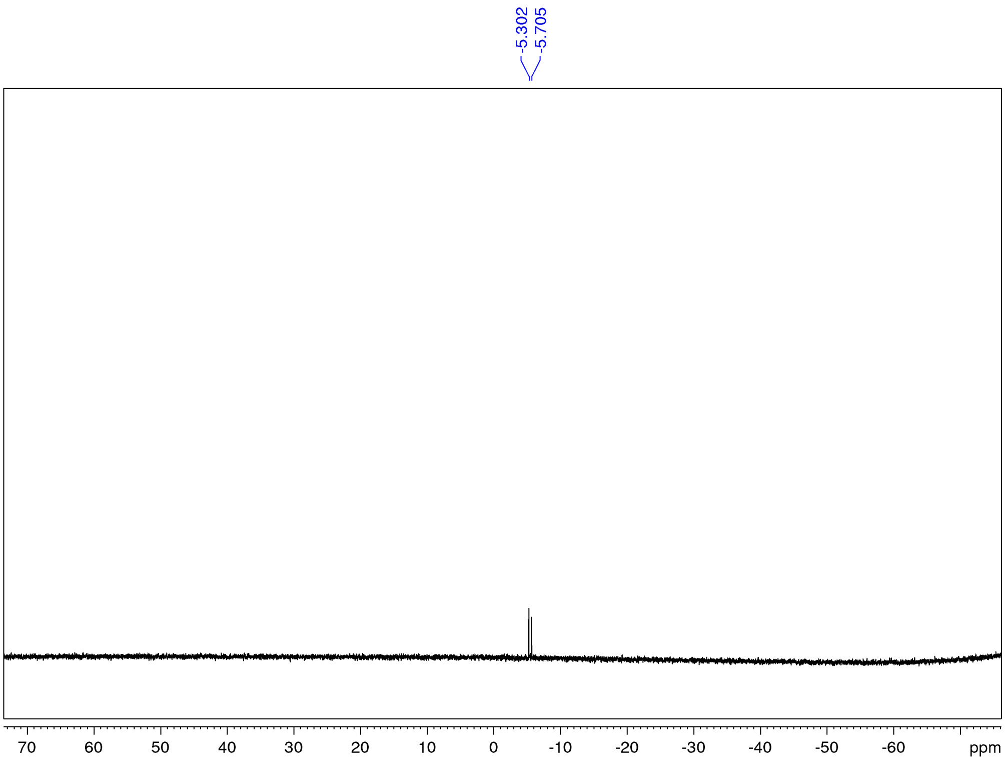

Figure S7. ^{29}Si NMR spectrum of (IMes)PSiMe$_3$ (**2b**) in C_6D_6 at room temperature.

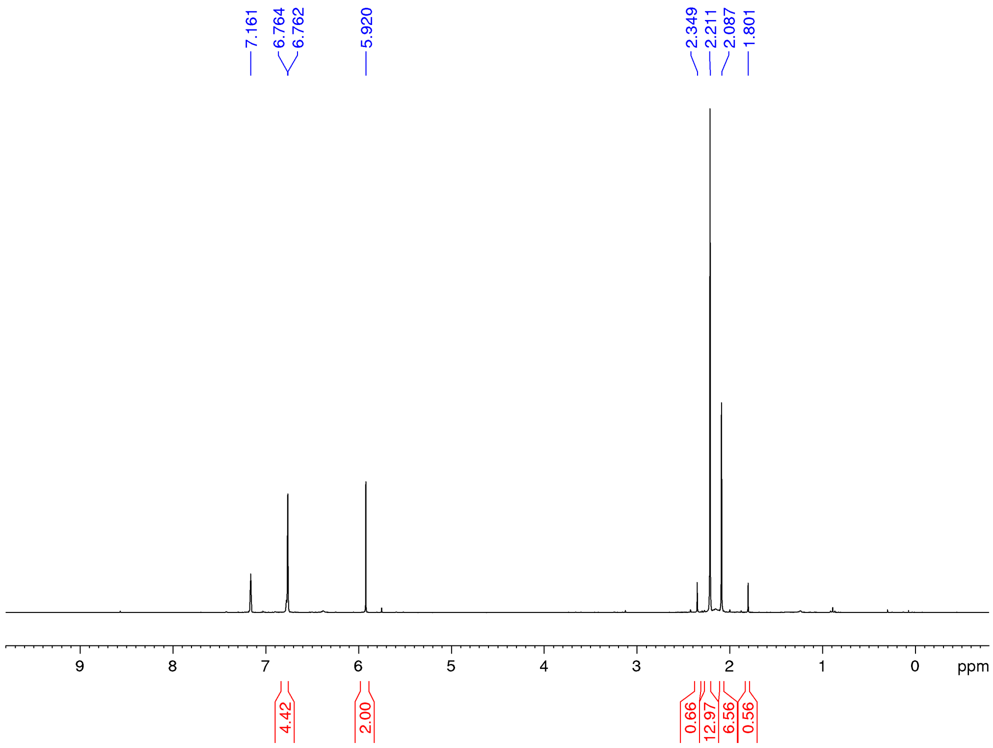

Figure S8. ^{1}H NMR spectrum of (IMes)PH (**3b**) in C_6D_6 at room temperature.

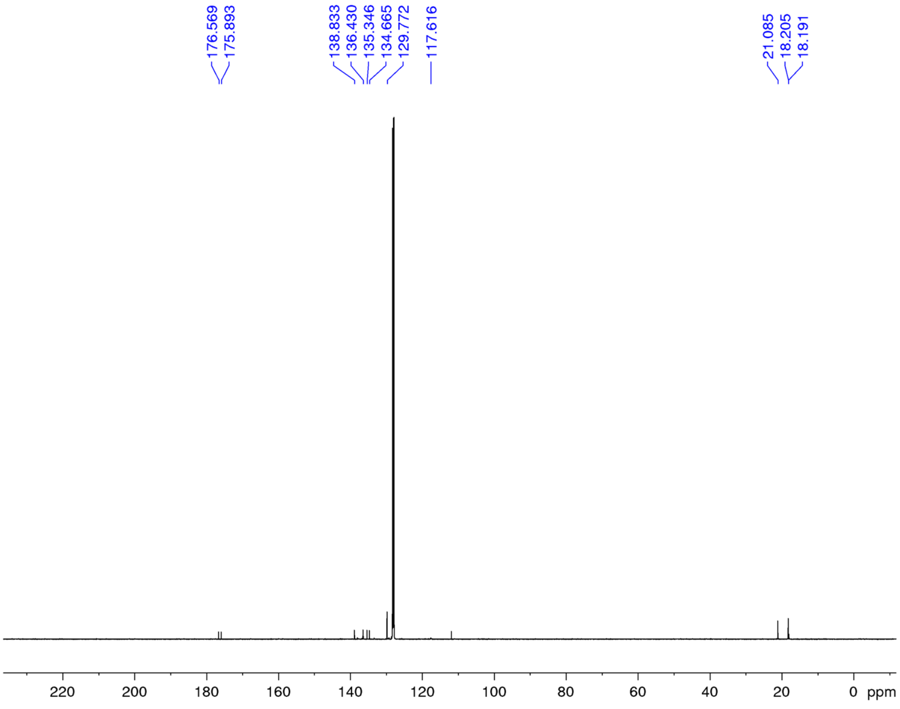

Figure S9. ^{13}C NMR spectrum of (IMes)PH (**3b**) in C_6D_6 at room temperature.

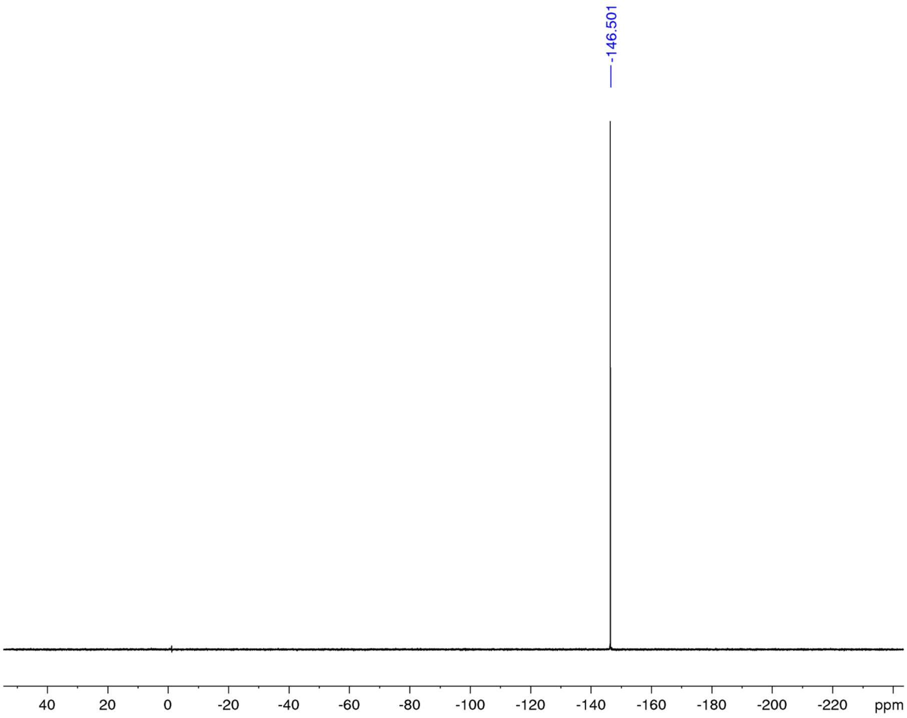

Figure S10. $^{31}P\{^{1}H\}$ NMR spectrum of (IMes)PH (**3b**) in C_6D_6 at room temperature.

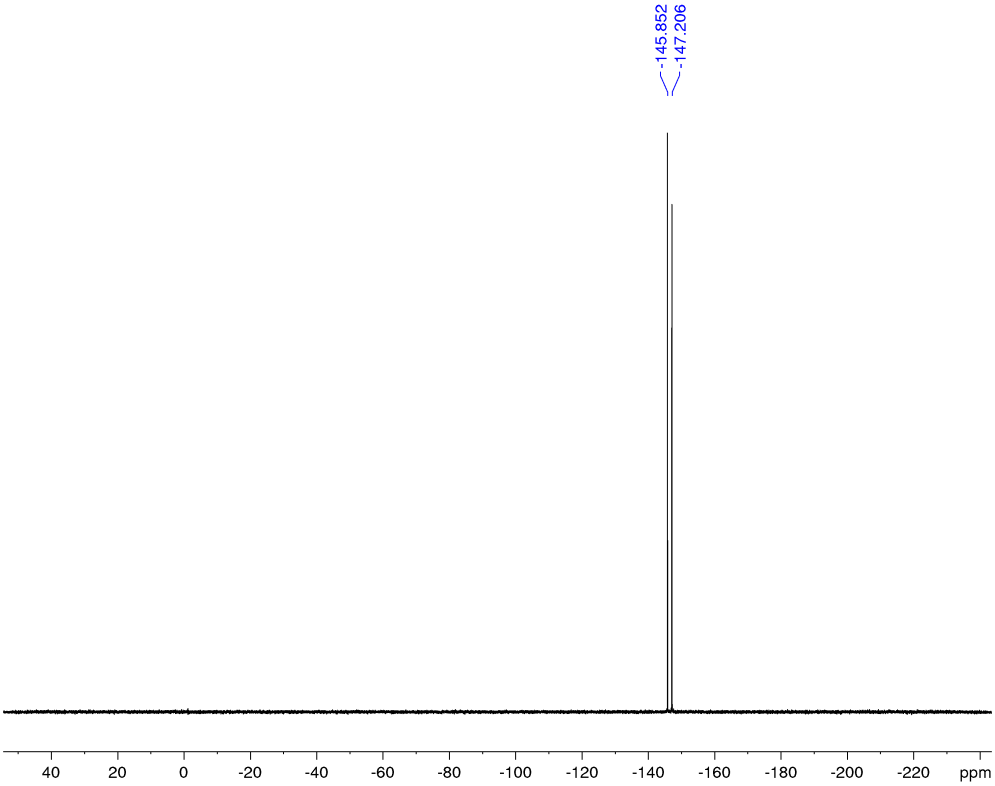

Figure S11. ^{31}P NMR spectrum of (IMes)PH (**3b**) in C_6D_6 at room temperature.

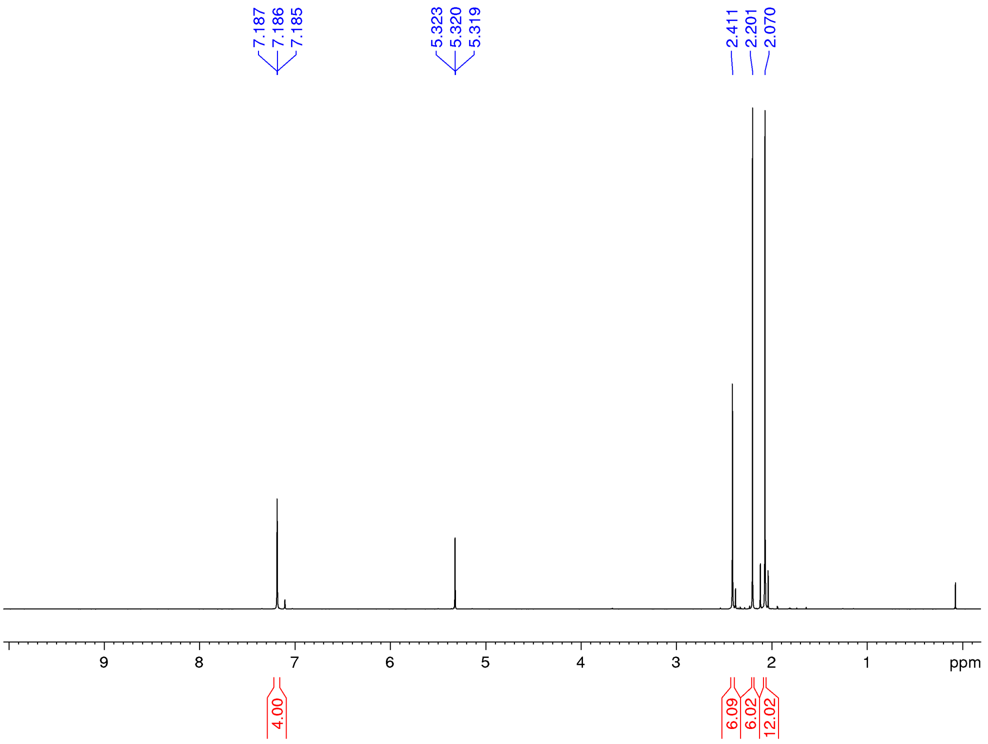

Figure S12. ^{1}H NMR spectrum of $[^{Me}IMesCl]Cl$ in CD_2Cl_2 at room temperature.

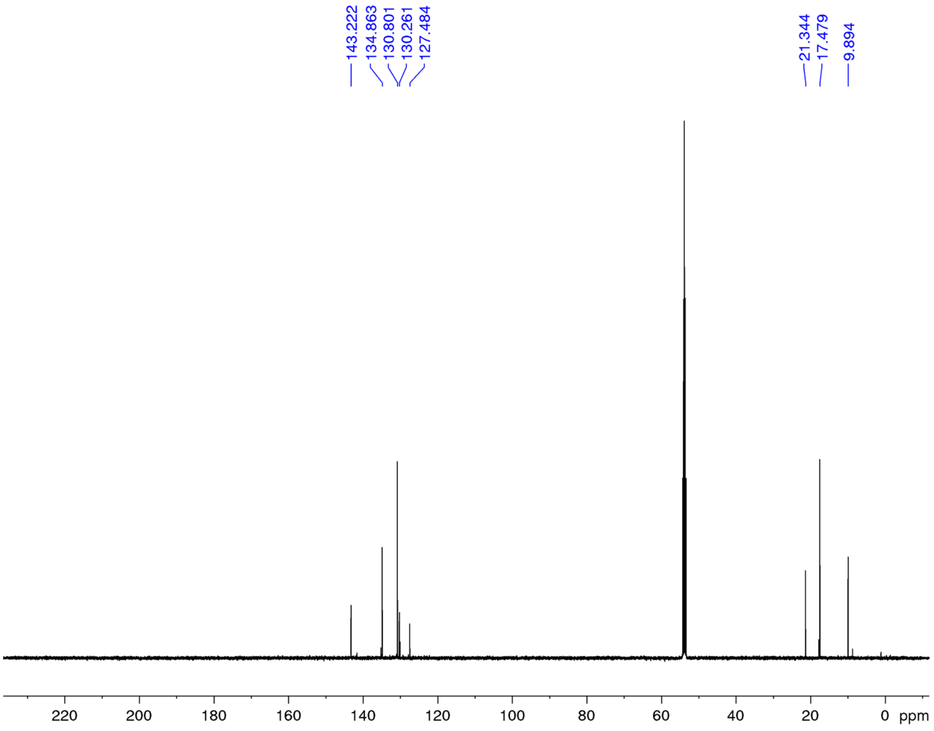

Figure S13. ^{13}C NMR spectrum of $[^{Me}IMesCl]Cl$ in CD_2Cl_2 at room temperature.

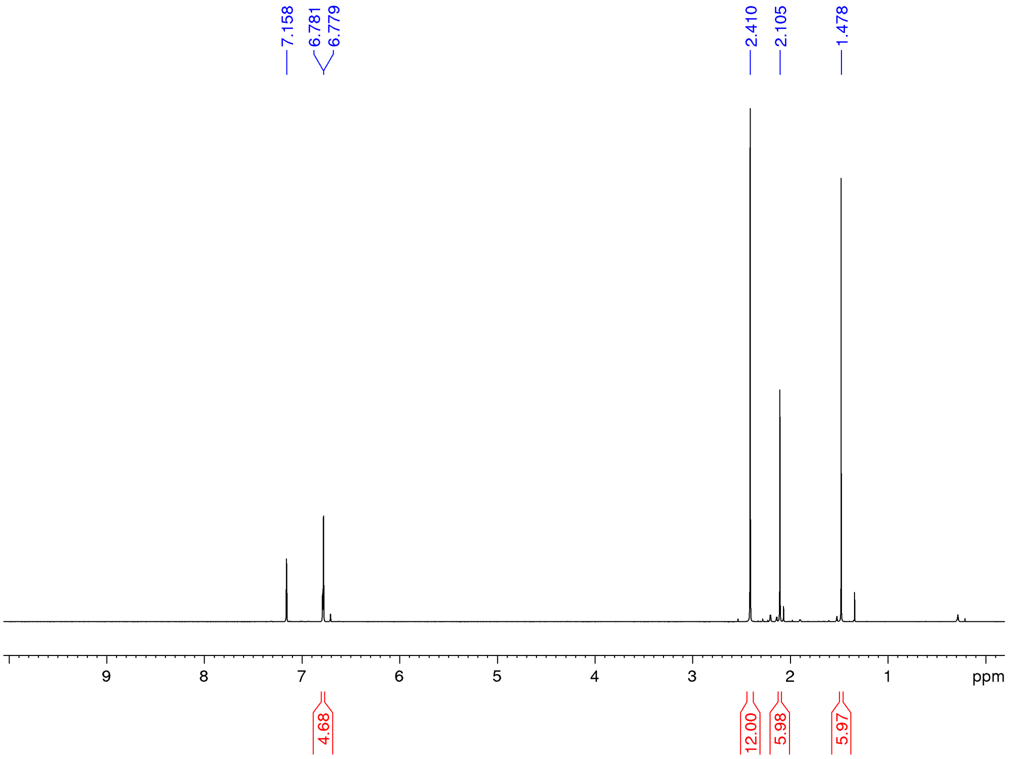

Figure S14. ^{1}H NMR spectrum of (^{Me}IMes)F_2 (**1c**) in C_6D_6 at room temperature.

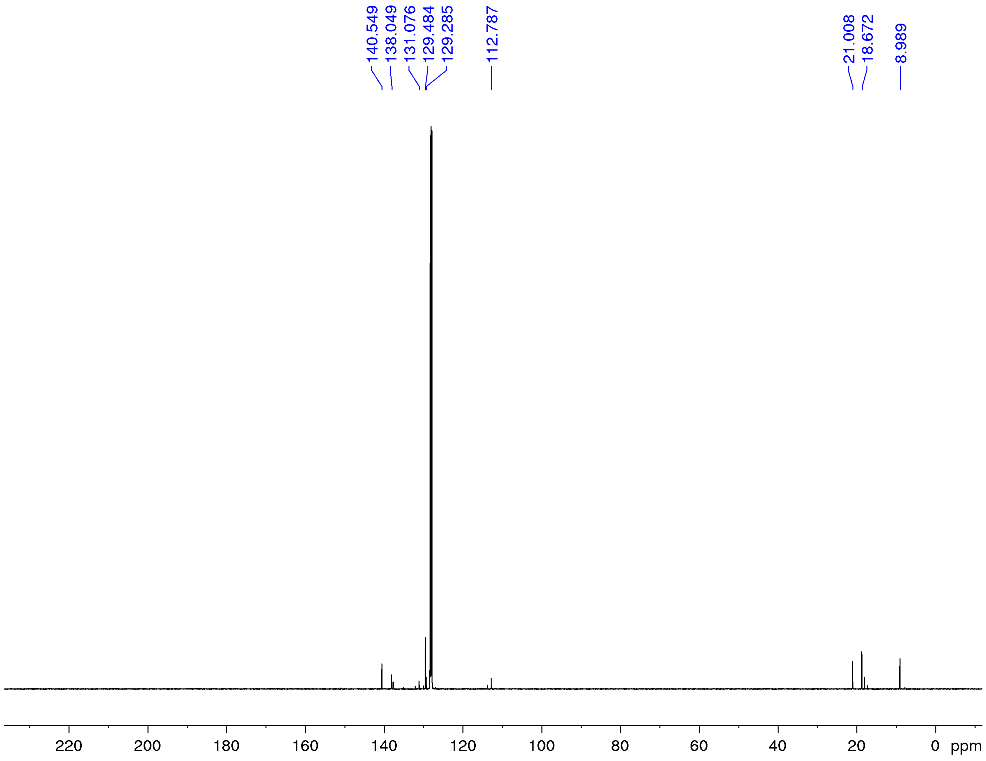

Figure S15. ^{13}C NMR spectrum of (^{Me}IMes)F_2 (**1c**) in C_6D_6 at room temperature.

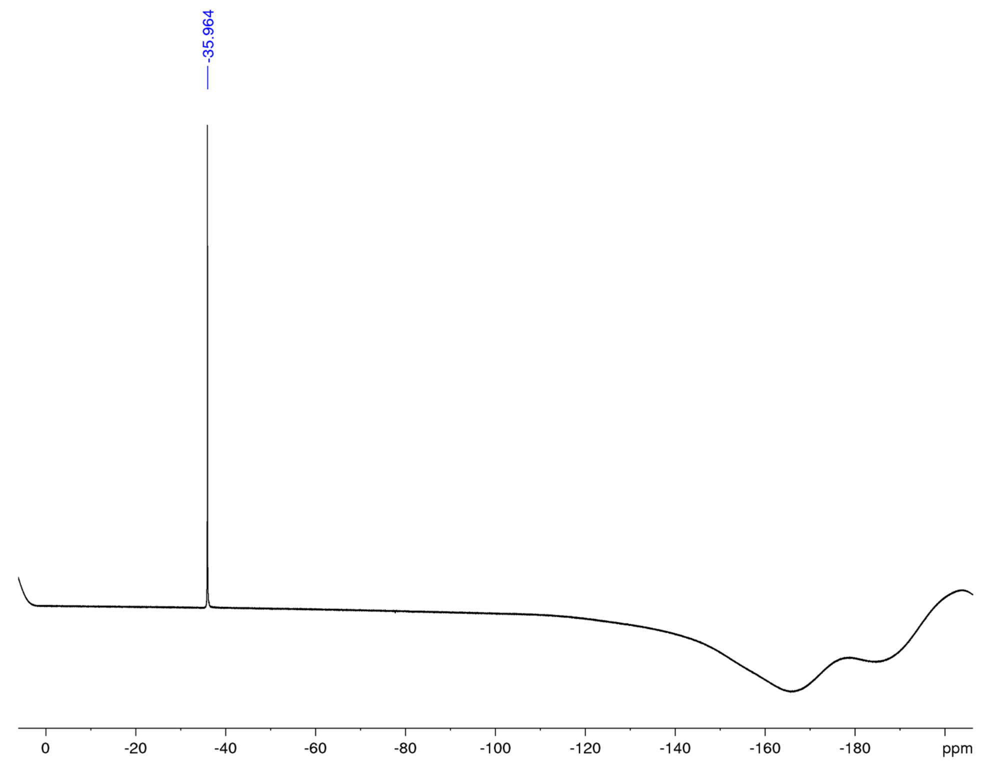

Figure S16. ^{19}F NMR spectrum of (^{Me}IMes)F_2 (**1c**) in C_6D_6 at room temperature.

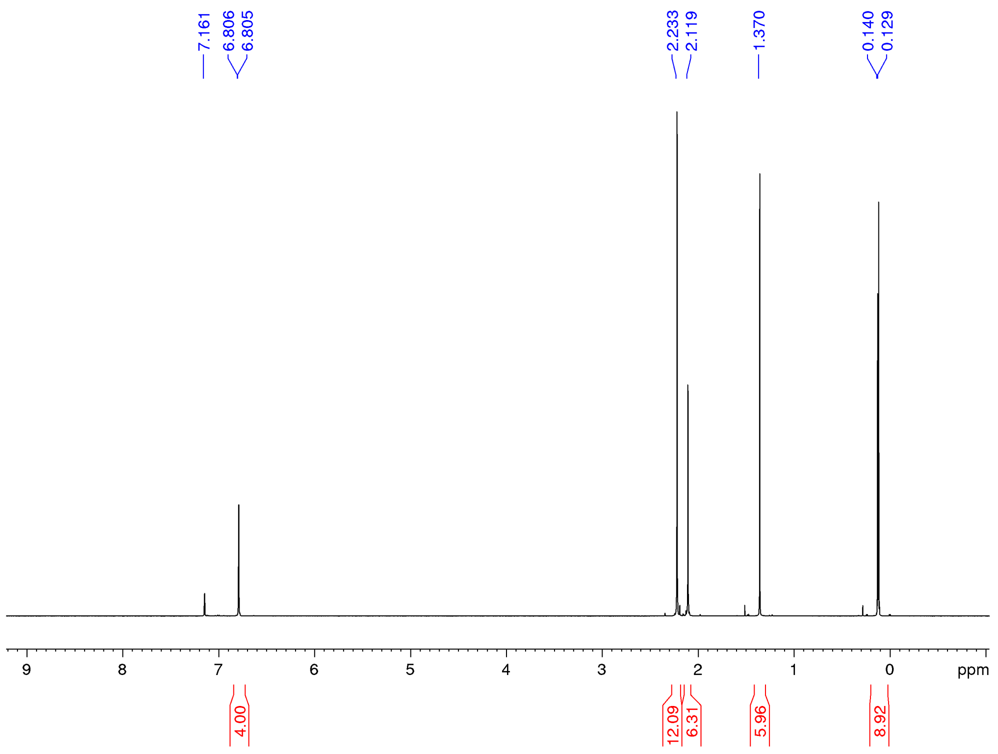

Figure S17. ^{1}H NMR spectrum of (^{Me}IMes)PSiMe$_3$ (**2c**) in C_6D_6 at room temperature.

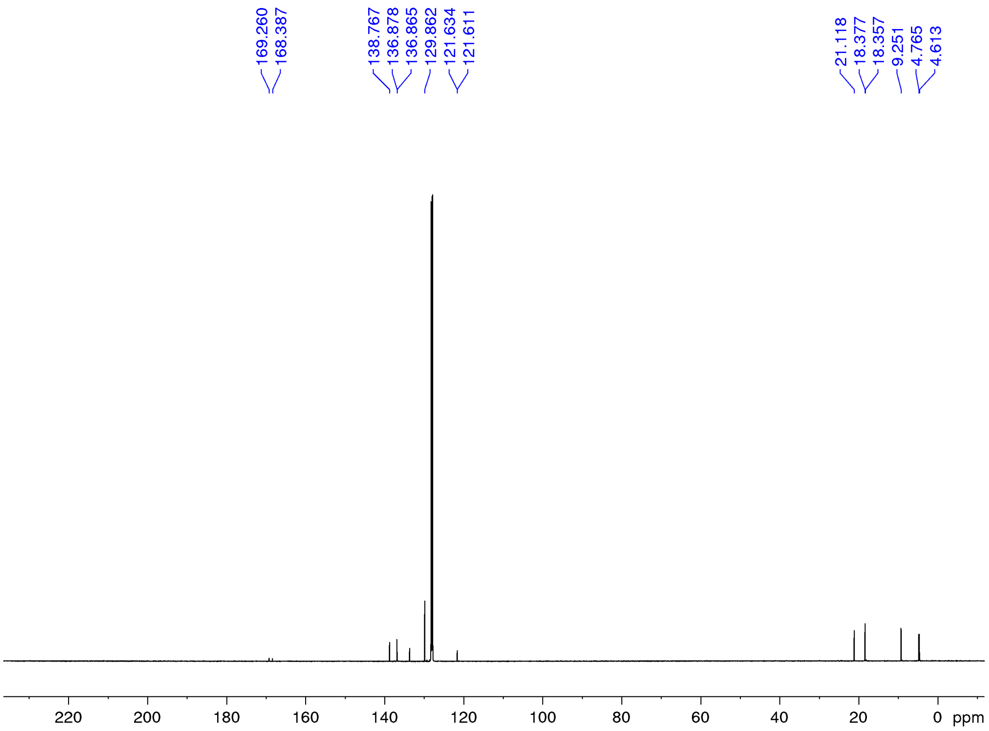

Figure S18. ^{13}C NMR spectrum of (^{Me}IMes)$PSiMe_3$ (**2c**) in C_6D_6 at room temperature.

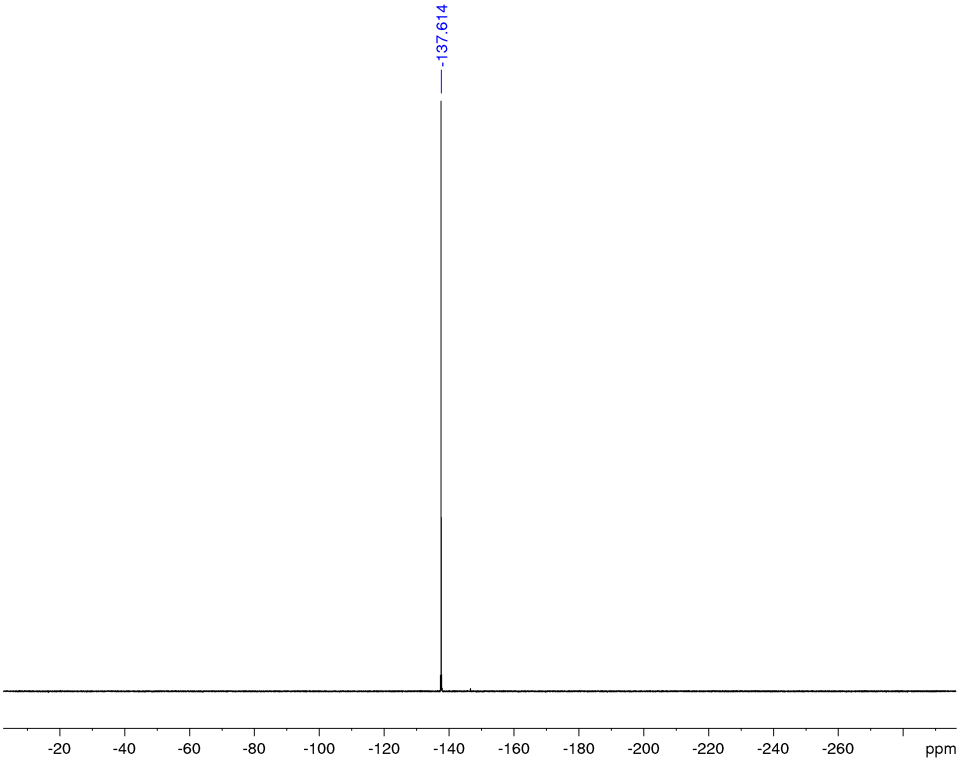

Figure S19. ^{31}P {^{1}H} NMR spectrum of (^{Me}IMes)$PSiMe_3$ (**2c**) in C_6D_6 at room temperature.

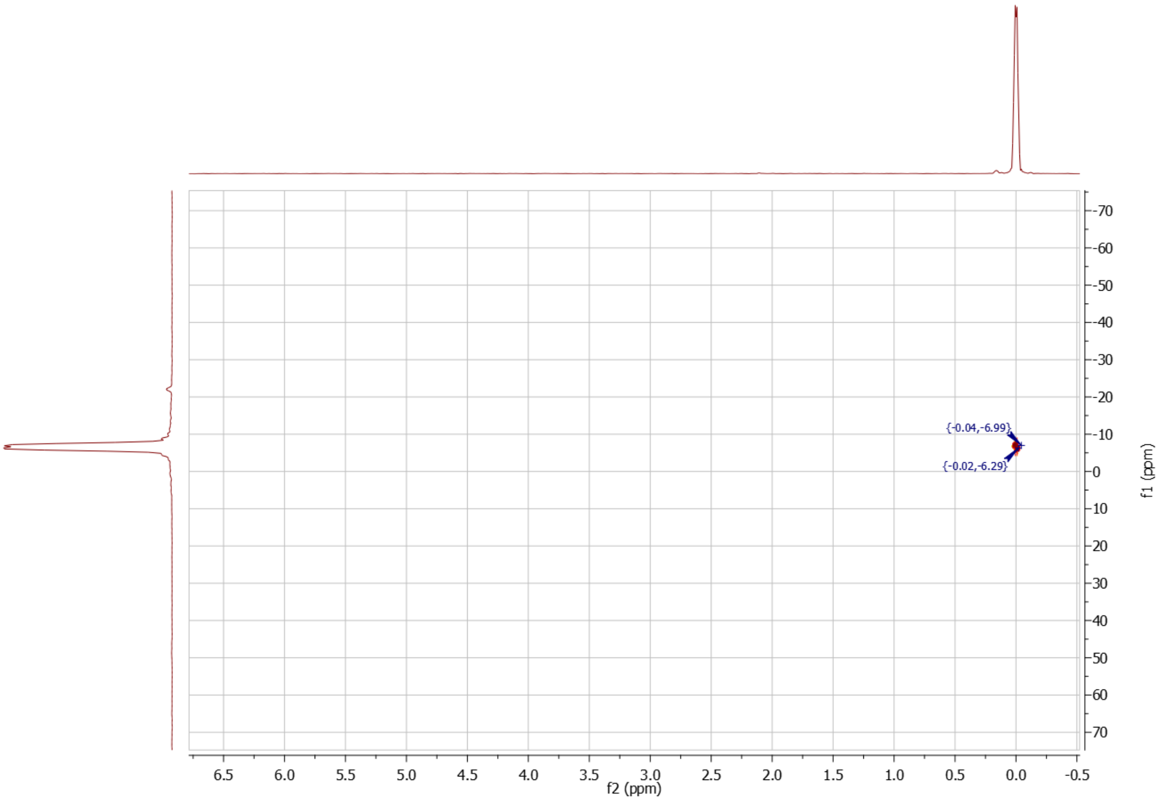

Figure S20. HMBC NMR spectrum of (^{Me}IMes)PSiMe$_3$ (**2c**) in C_6D_6 at room temperature.

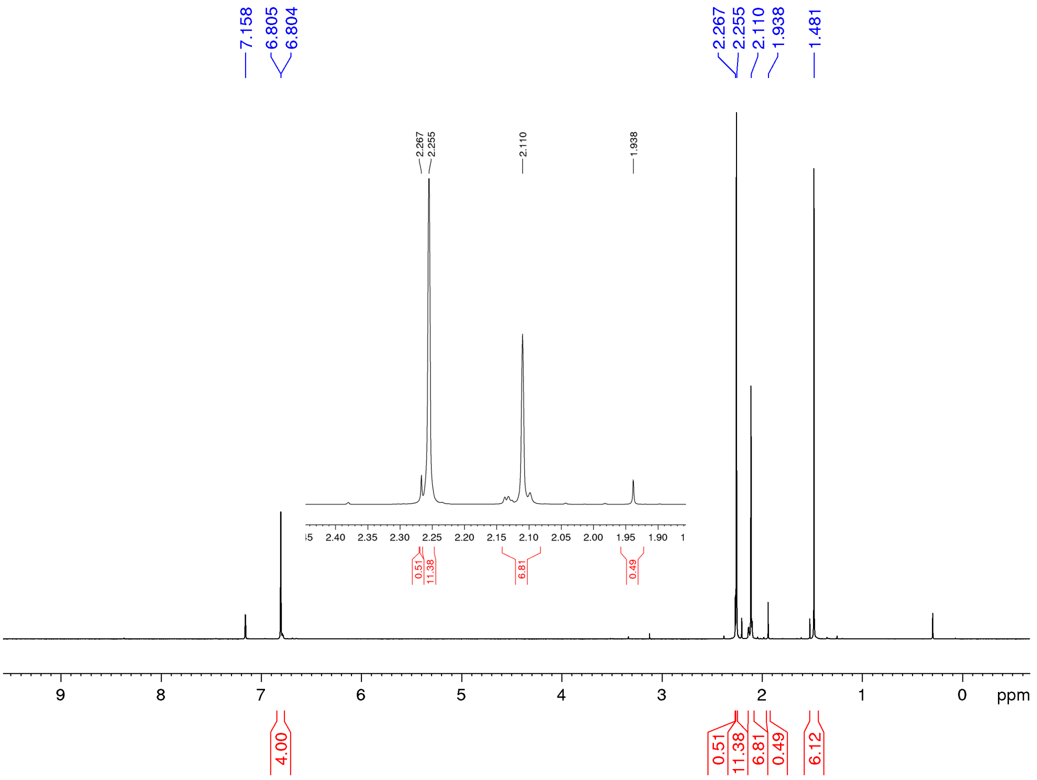

Figure S21. ^{1}H NMR spectrum of (^{Me}IMes)PH (**3c**) in C_6D_6 at room temperature.

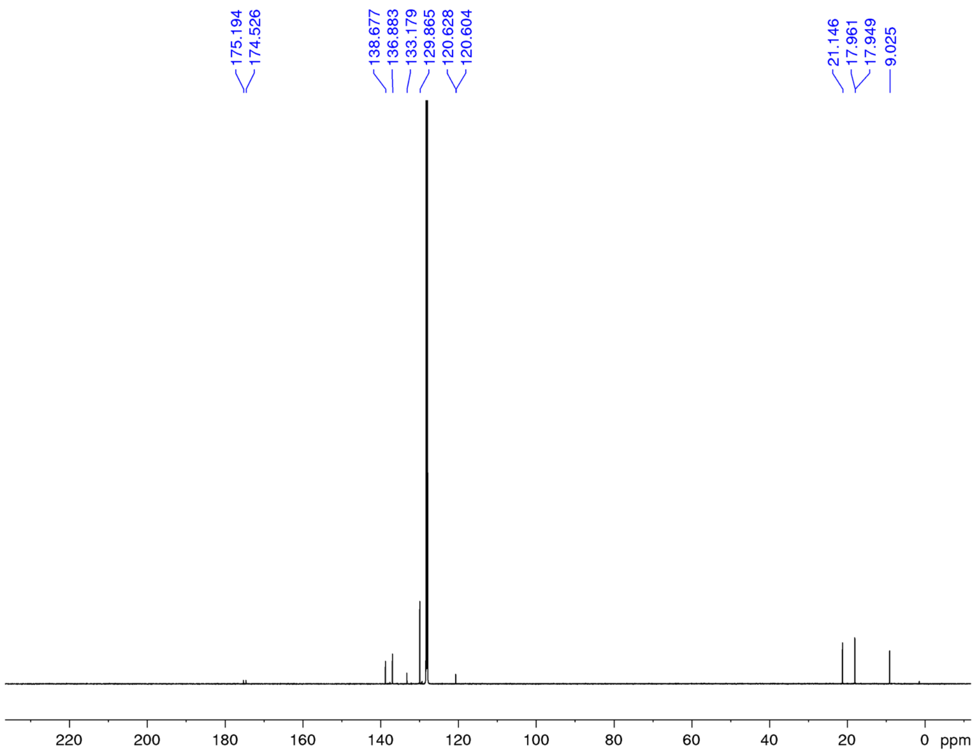

Figure S22. ^{13}C NMR spectrum of (^{Me}IMes)PH (**3c**) in C_6D_6 at room temperature.

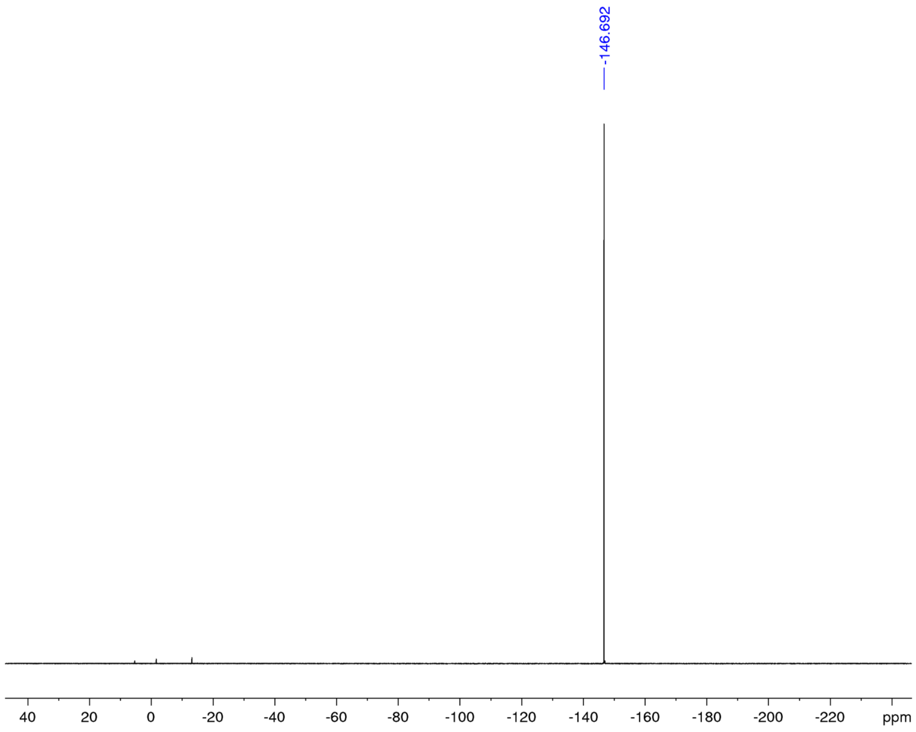

Figure S23. ^{31}P {^{1}H} NMR spectrum of (^{Me}IMes)PH (**3c**) in C_6D_6 at room temperature.

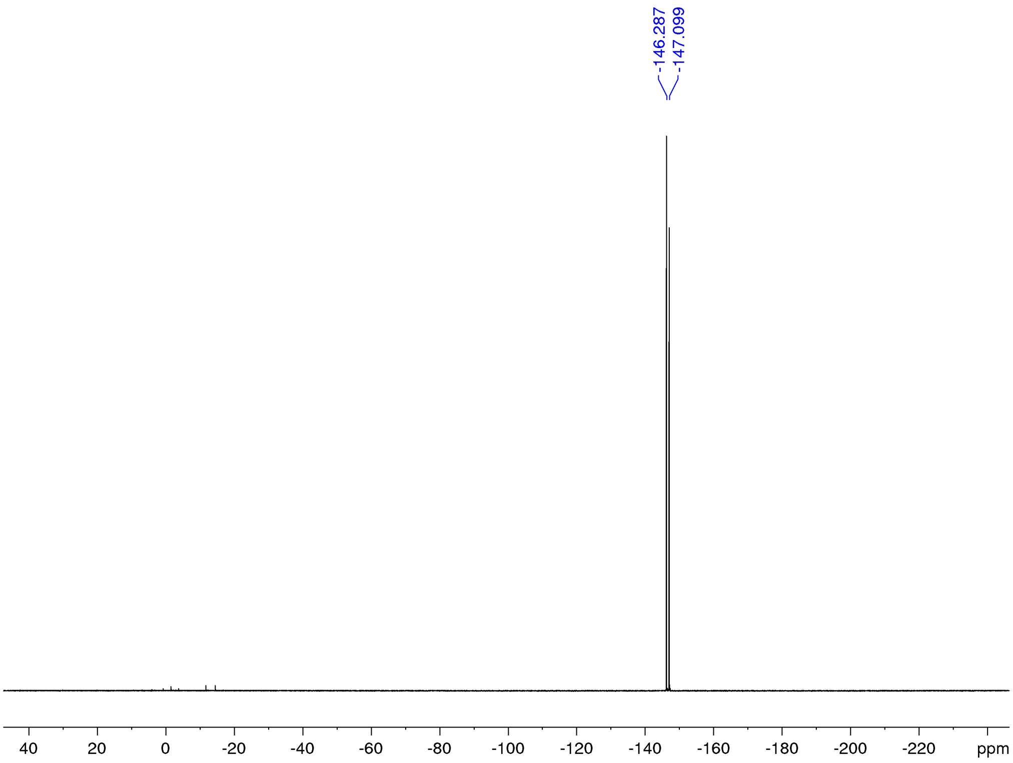

Figure S24. ^{31}P NMR spectrum of (^{Me}IMes)PH (**3c**) in C_6D_6 at room temperature.

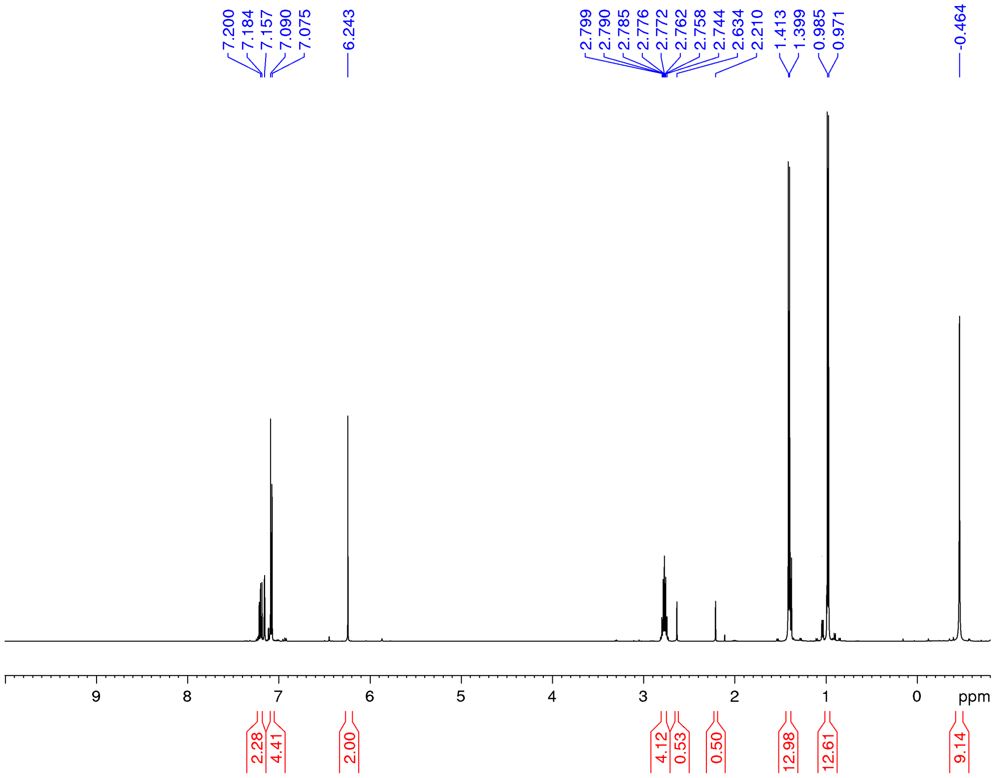

Figure S25. ^{1}H NMR spectrum of [{(IDipp)PH}AlMe$_3$] (**4a**) in C_6D_6 at room temperature.

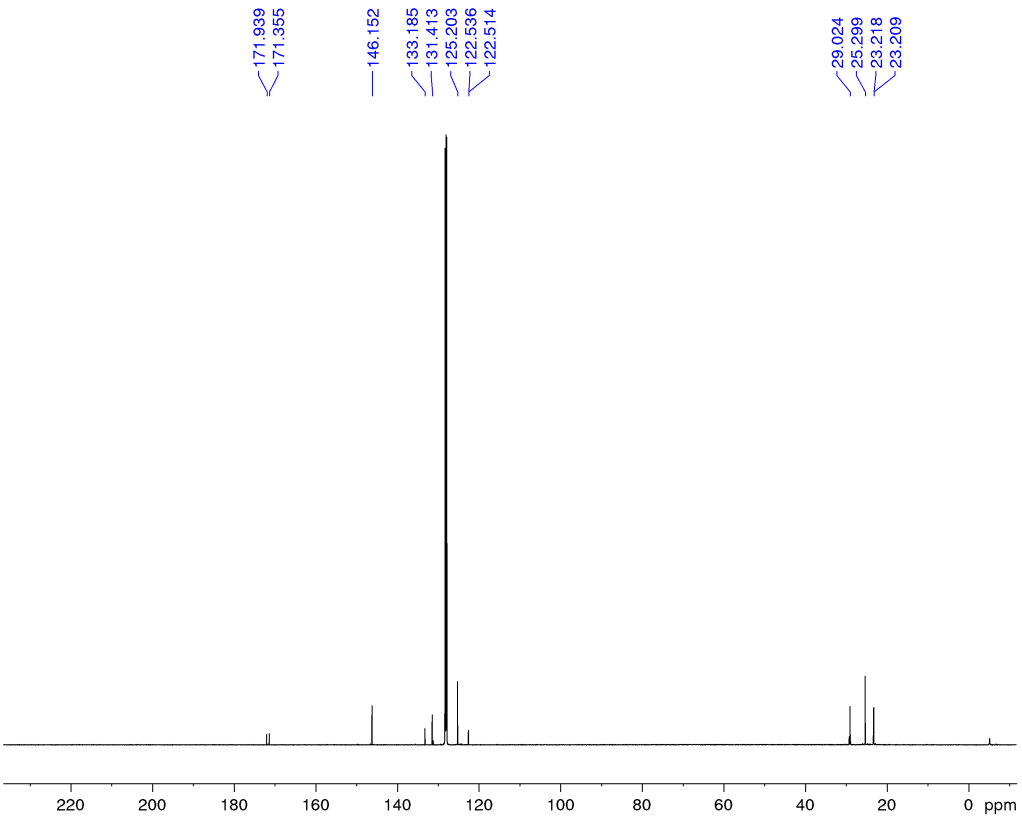

Figure S26. ^{13}C NMR spectrum of [{(IDipp)PH}AlMe$_3$](**4a**) in C_6D_6 at room temperature.

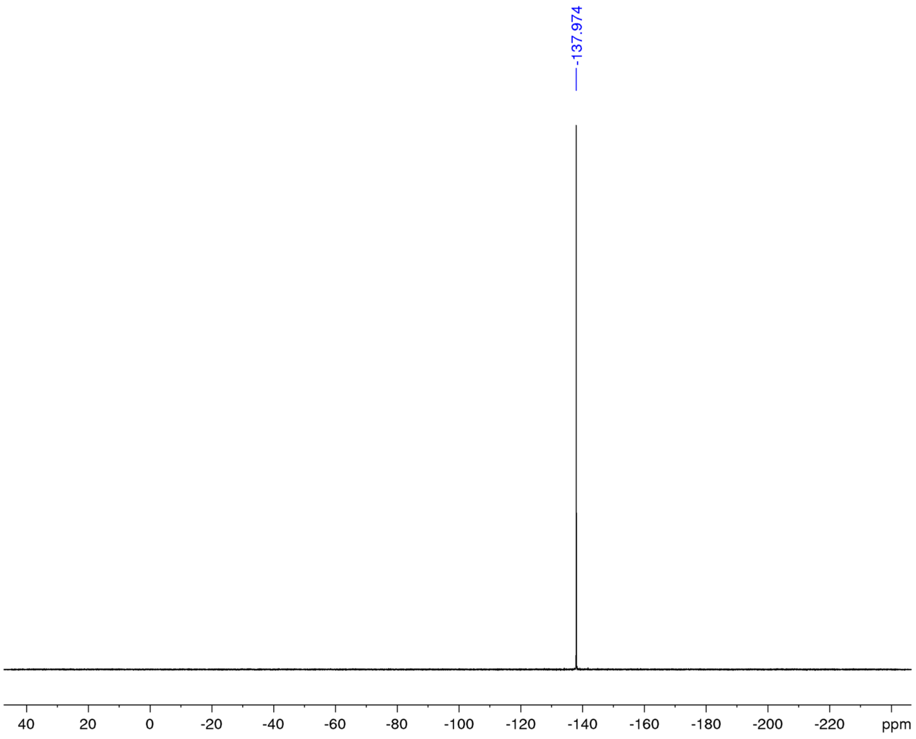

Figure S27. ^{31}P {^{1}H} NMRspectrum of [{(IDipp)PH}AlMe$_3$] (**4a**) in C_6D_6 at room temperature.

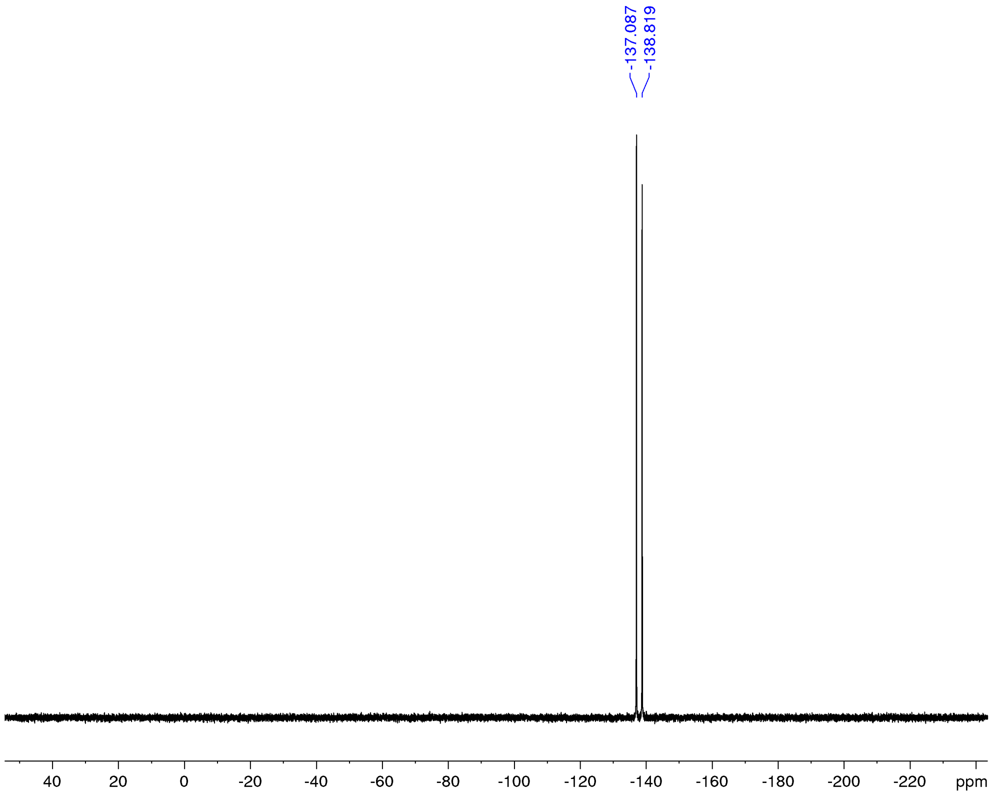

Figure S28. ^{31}P NMR spectrum of [{(IDipp)PH}AlMe$_3$] (**4a**) in C_6D_6 at room temperature.

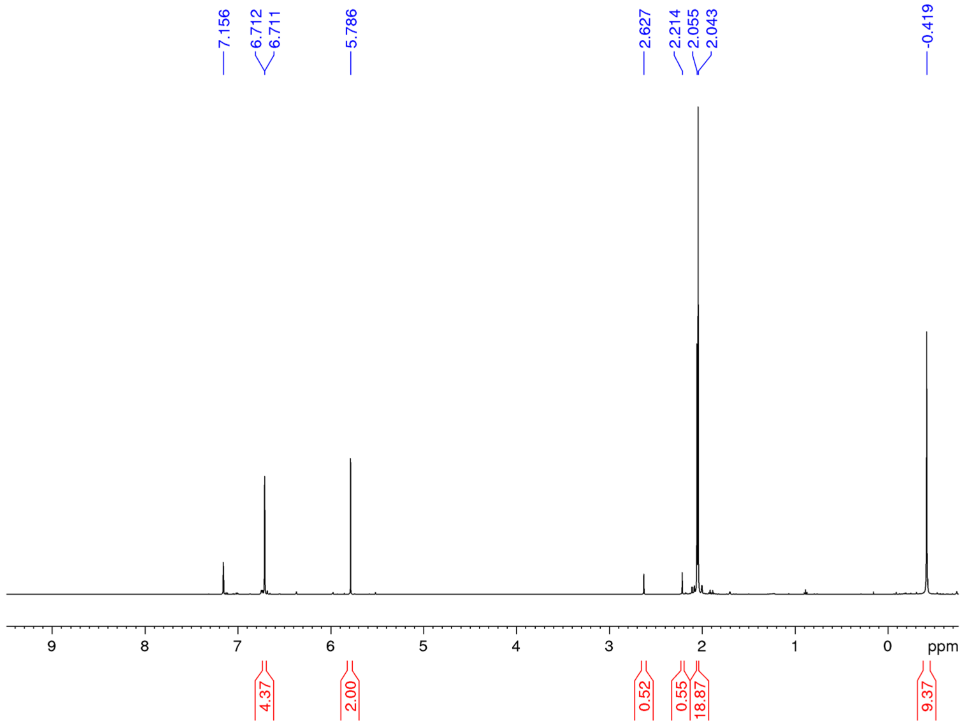

Figure S29. ^{1}H NMR spectrum of [{(IMes)PH}AlMe$_3$] (**4b**) in C_6D_6 at room temperature.

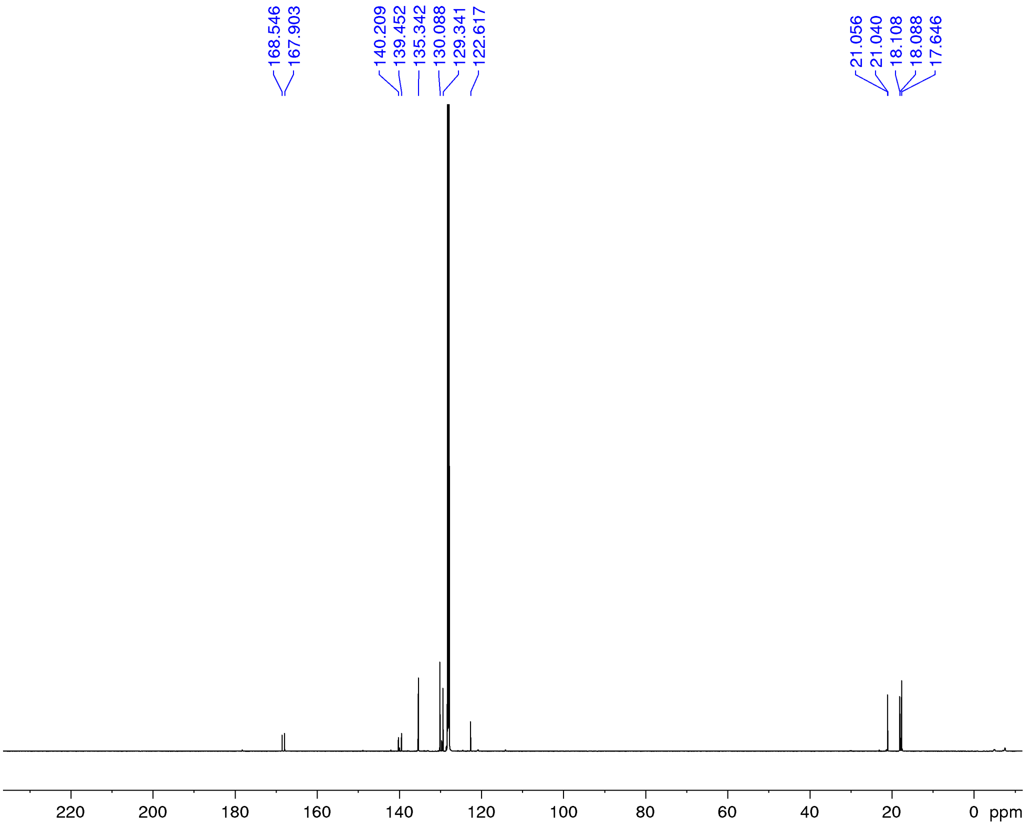

Figure S30. ^{13}C NMR spectrum of [{(IMes)PH}AlMe$_3$] (**4b**) in C_6D_6 at room temperature.

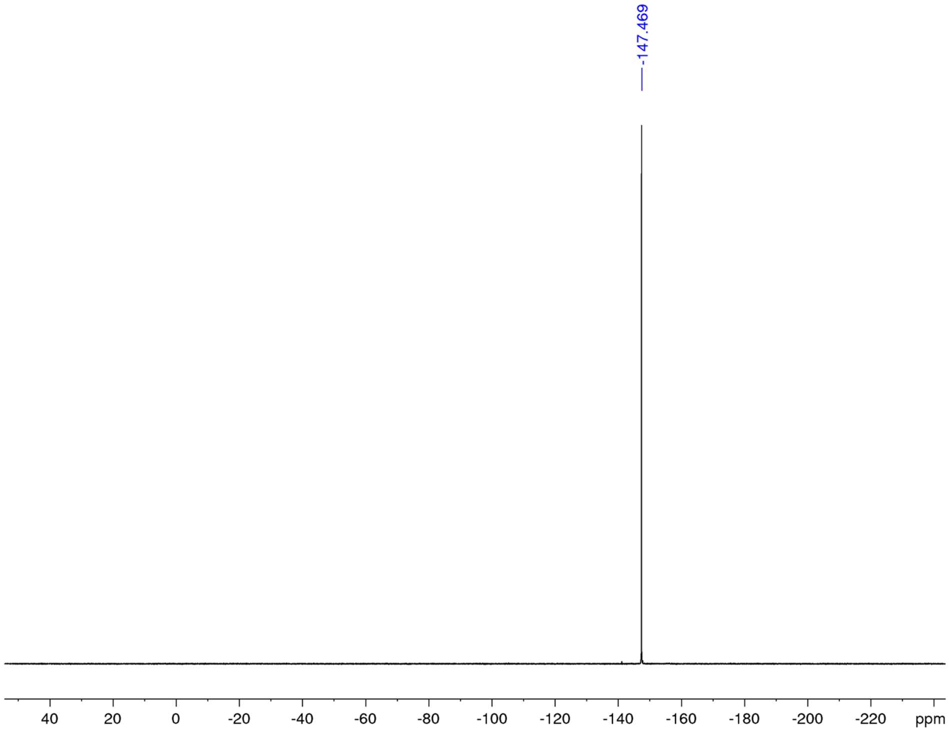

Figure S31. ^{31}P {^1H} NMR NMR spectrum of [{(IMes)PH}AlMe$_3$] (**4b**) in C_6D_6 at room temperature.

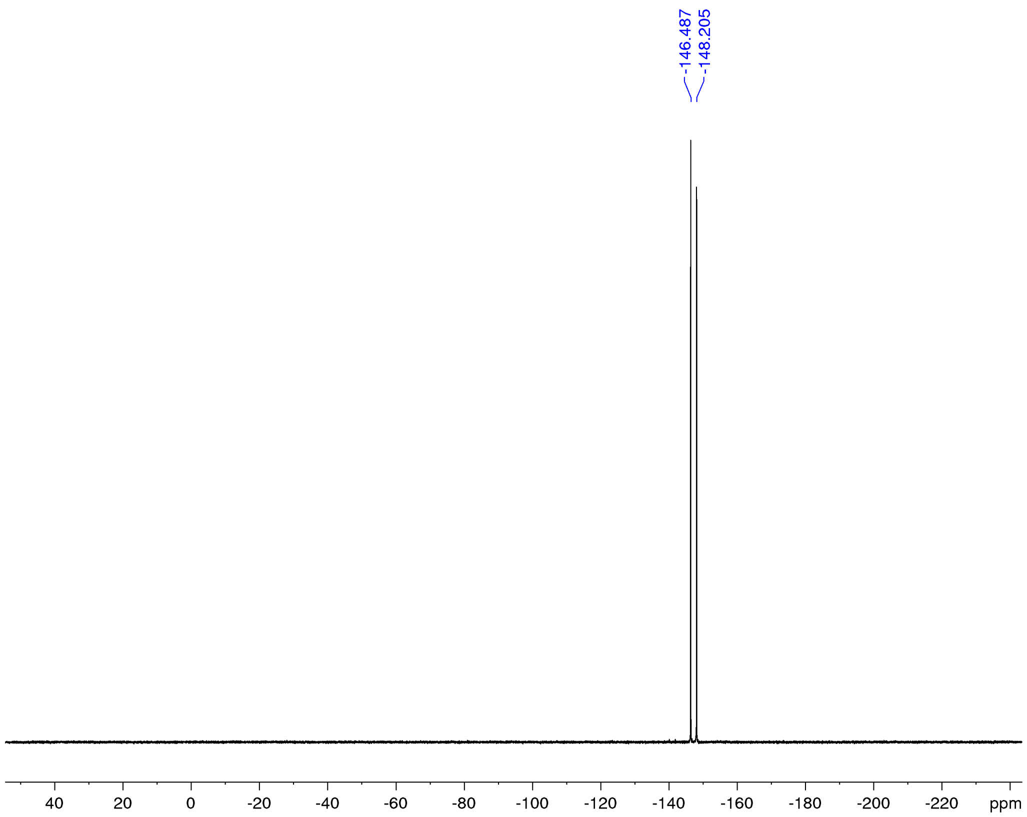

Figure S32. ^{31}P NMR spectrum of [{(IMes)PH}AlMe$_3$] (**4b**) in C_6D_6 at room temperature.

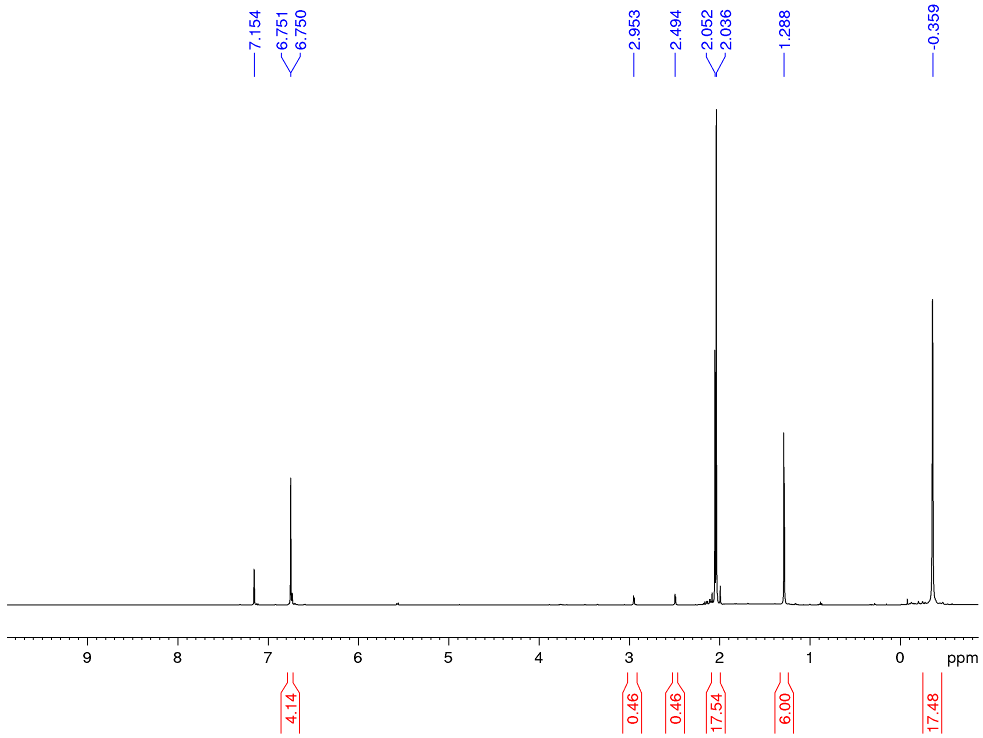

Figure S33. ^{1}H NMR spectrum of [{(^{Me}IMes)PH}(AlMe$_3$)$_2$] (**4c**) in C_6D_6 at room temperature.

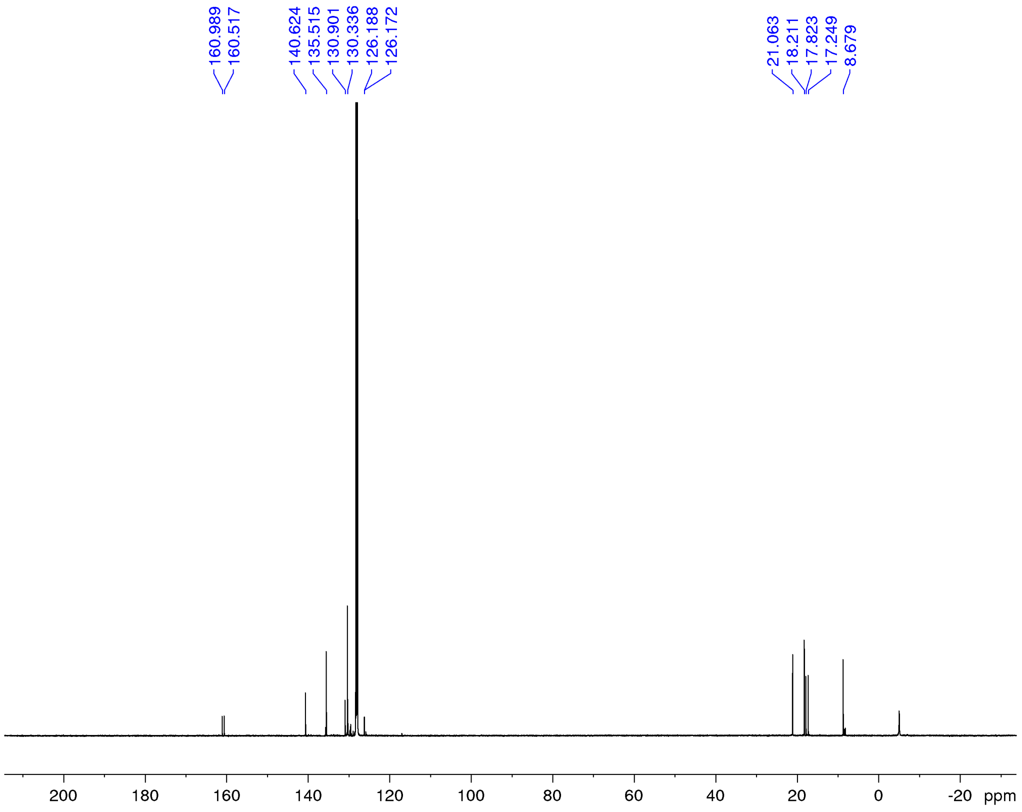

Figure S34. ^{13}C NMR spectrum of [{(^{Me}IMes)PH}($AlMe_3$)$_2$] (**4c**) in C_6D_6 at room temperature.

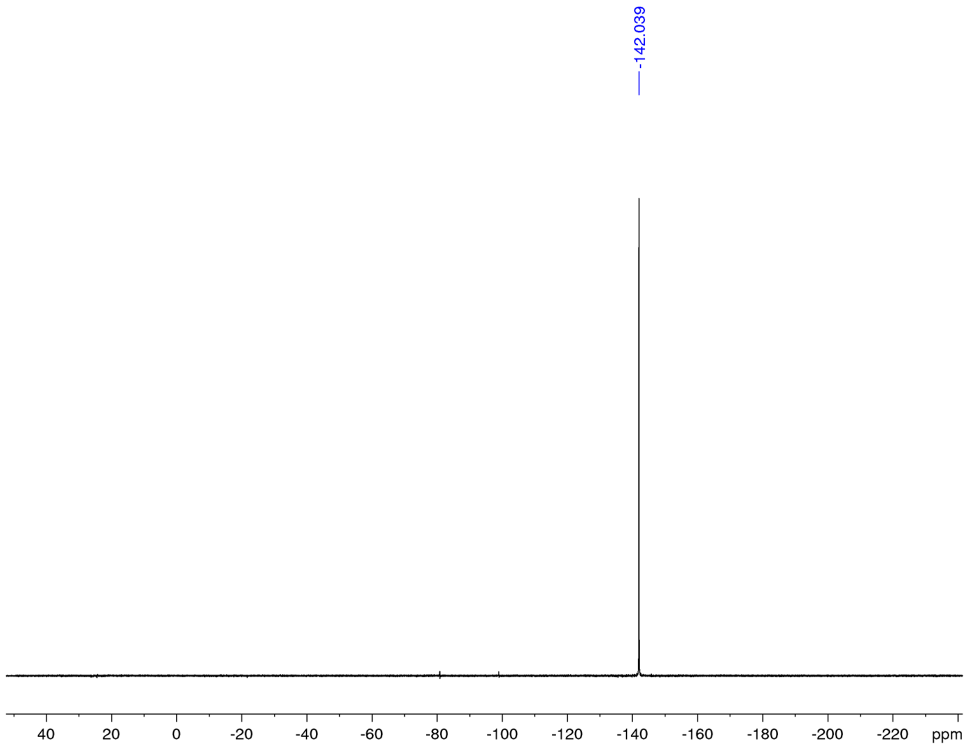

Figure S35. ^{31}P {^{1}H} NMR spectrum of [{(^{Me}IMes)PH}($AlMe_3$)$_2$] (**4c**) in C_6D_6 at room temperature.

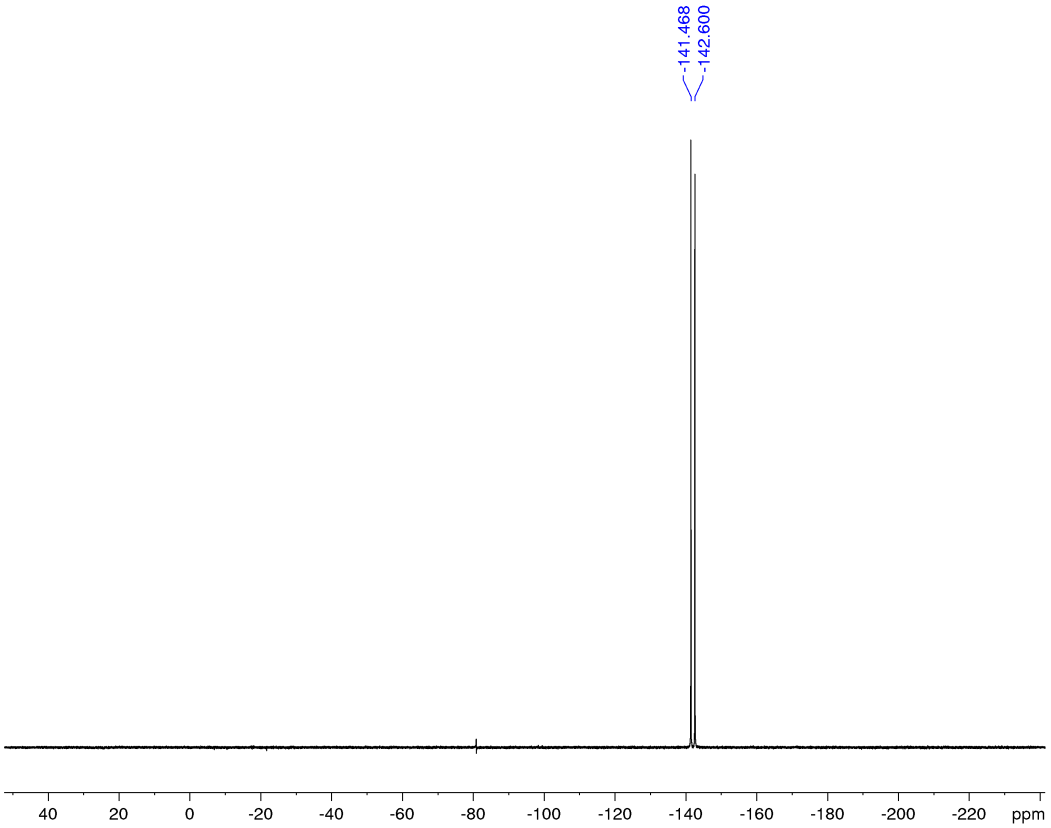

Figure S36. ^{31}P NMR spectrum of [{(^{Me}IMes)PH}($AlMe_3$)$_2$] (**4c**) in C_6D_6 at room temperature.

(S3) Investigation of the thermal stability of complexes 4a and 4b by NMR spectroscopy

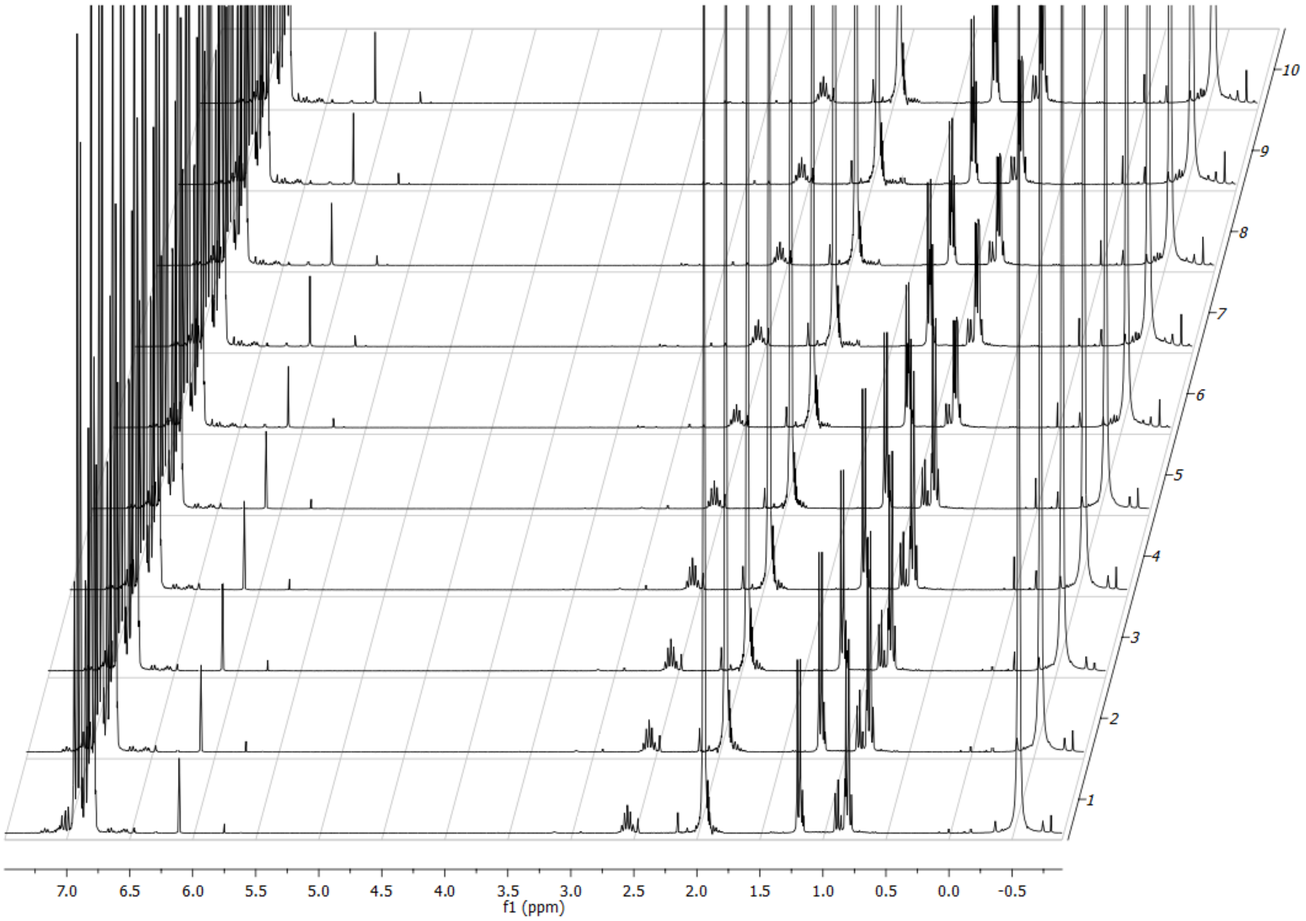

Figure S37. ^{1}H NMR spectra of **4a** in toluene-d_8 recorded after heating the sample for 20 min at each indicated temperature.

Entry of ^{1}H NMR spectra	Temperature (°C)	Entry of ^{1}H NMR spectra	Temperature (°C)
1	35	6	60
2	40	7	65
3	45	8	70
4	50	9	75
5	55	10	80[a]

[a] After heating the sample for an additional 6 h at 80 °C.

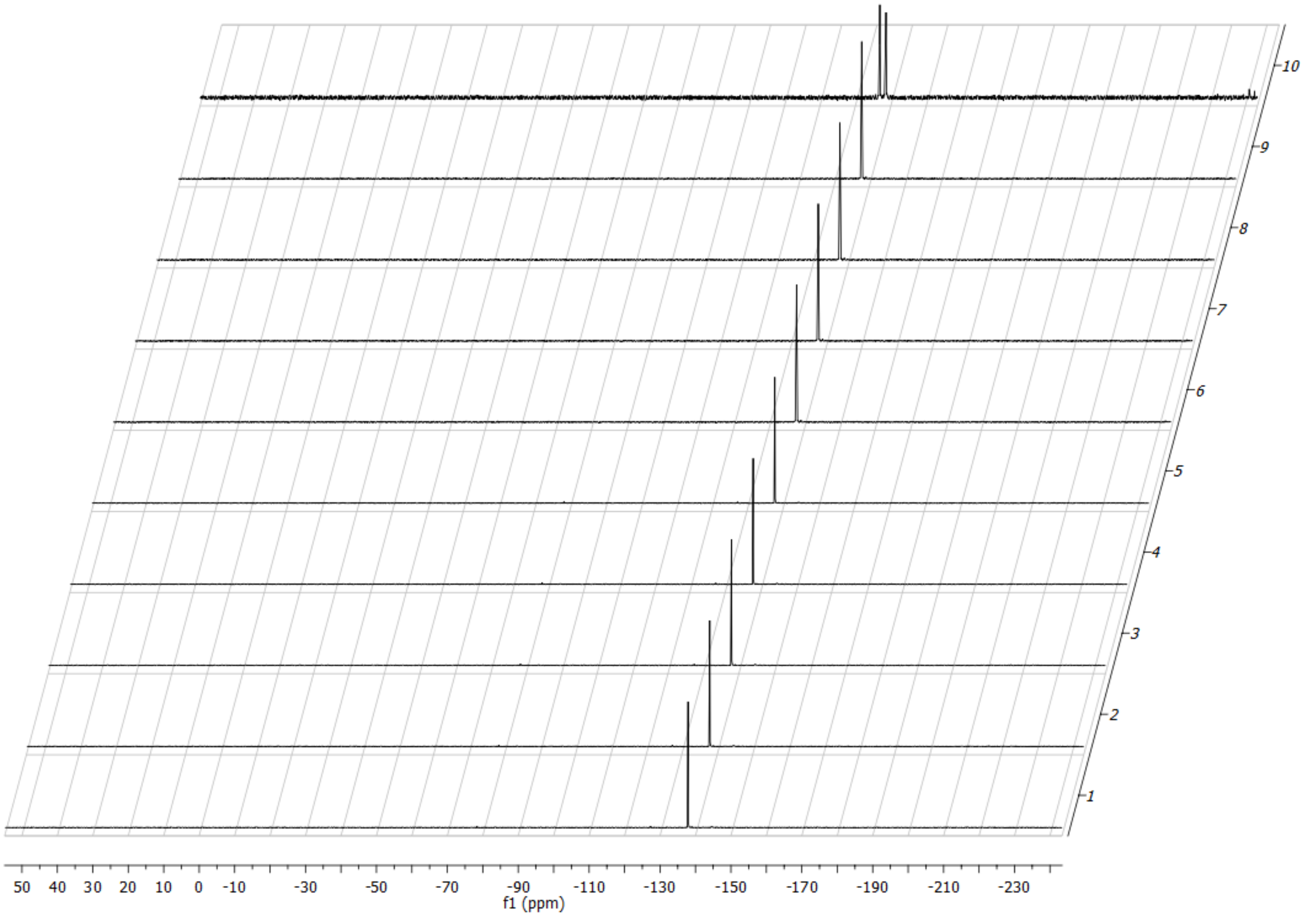

Figure S38. ^{31}P {^{1}H} NMR spectra of **4a** in toluene-d_8 recorded after heating the sample for 20 min at each indicated temperature.

Entry of ^{31}P {^{1}H} NMR spectra	Temperature (°C)	Entry of ^{31}P {^{1}H} NMR spectra	Temperature (°C)
1	35	6	65
2	45	7	70
3	50	8	75
4	55	9	80[a]
5	60	10	80[a, b]

[a] After heating the sample for an additional 6 h at 80 °C. [b] ^{31}P NMR spectrum.

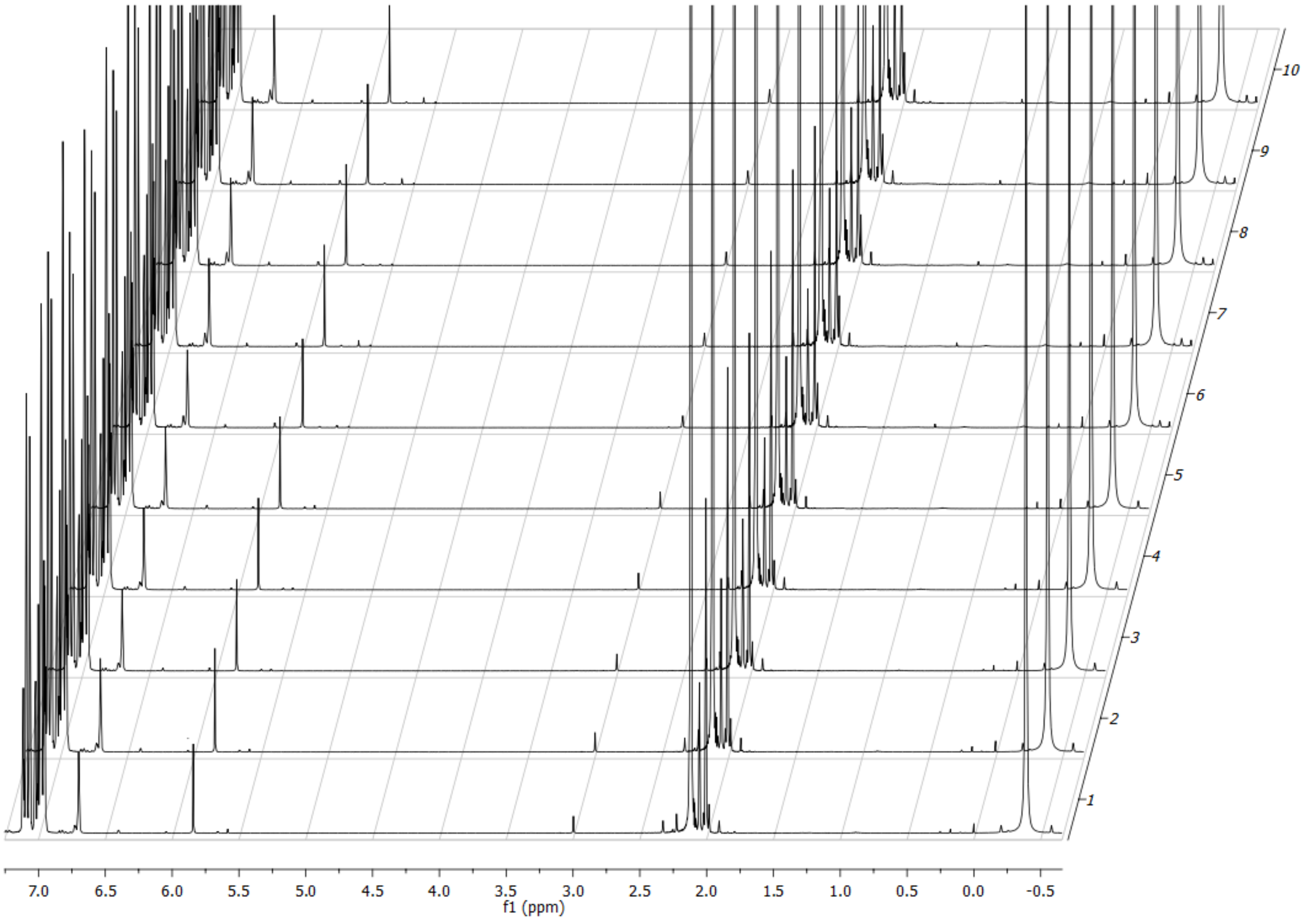

Figure S39. ^{1}H NMR spectra of **4b** in toluene-d_8 recorded after heating the sample for 20 min at each indicated temperature.

Entry of ^{1}H NMR spectra	Temperature (°C)	Entry of ^{1}H NMR spectra	Temperature (°C)
1	35	6	60
2	40	7	65
3	45	8	70
4	50	9	75
5	55	10	80[a]

[a] After heating the sample for an additional 6 h at 80 °C.

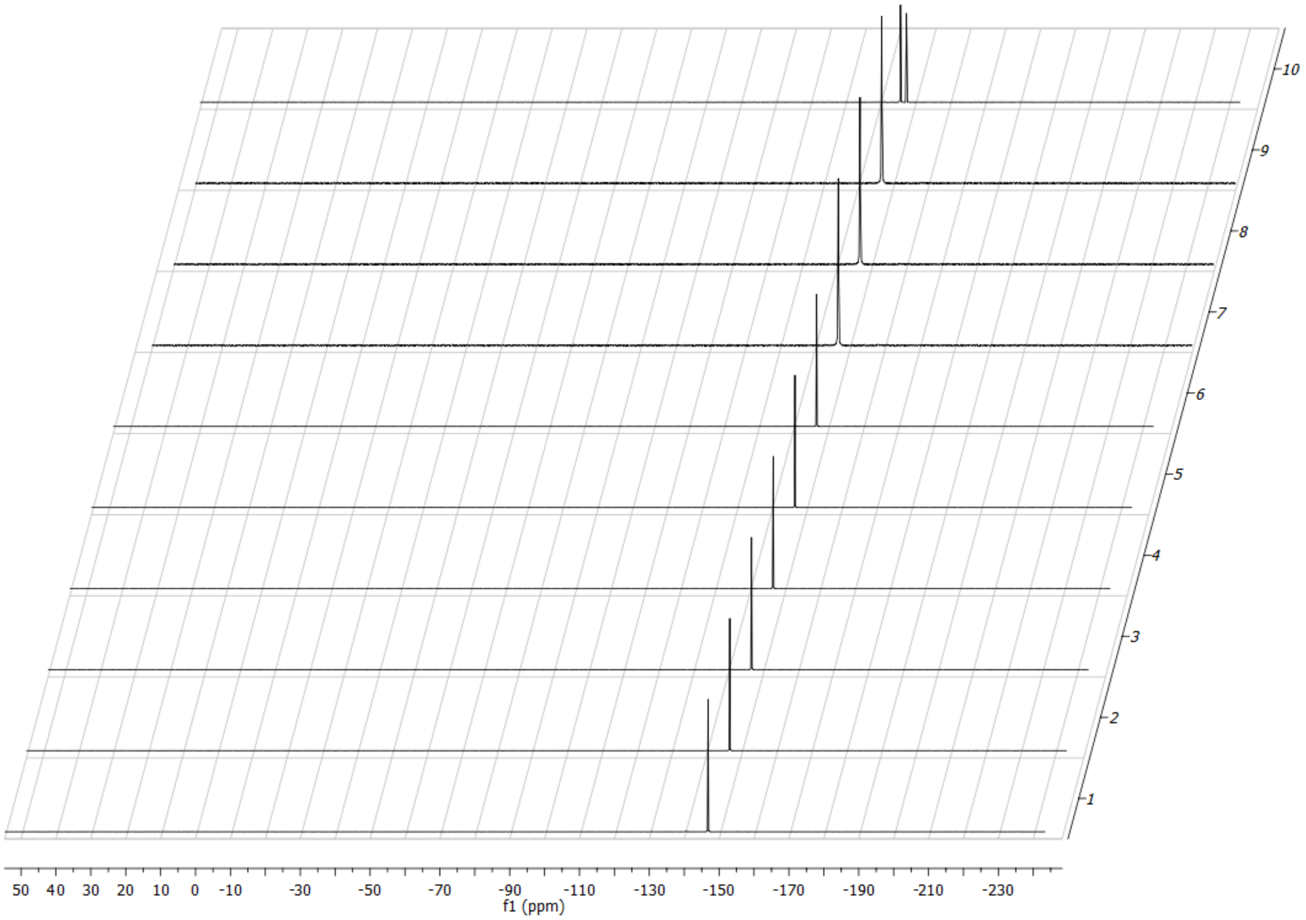

Figure S40. $^{31}P\{^1H\}$ NMR spectra of **4b** in toluene-d_8 recorded after heating the sample for 20 min at each indicated temperature.

Entry of $^{31}P\{^1H\}$ NMR spectra	Temperature (°C)	Entry of $^{31}P\{^1H\}$ NMR spectra	Temperature (°C)
1	35	6	65
2	45	7	70
3	50	8	75
4	55	9	80[a]
5	60	10	80[a, b]

[a] After heating the sample for an additional 6 h at 80 °C. [b] ^{31}P NMR spectrum.

(S4) Experimental procedure for polymerization

Typical polymerization of *rac*-lactide.

rac-LA 100–1000 mg (0.694–6.94 mmol) was added to a solution of **4a**–**4c** (6.94 μmol) in toluene (1 mL). Subsequently, the solution was stirred at 60 °C for 10–16 h depending on nature of initiator. The reaction was then quenched by the addition of a drop of HCl (2 N) and methanol. Then the solution was concentrated under vacuum, and the polymer was recrystallized from dichloromethane and *n*-hexane. The final polymer was then dried under vacuum to constant weight.

Gel Permeation Chromatography

Measurements were performed on a PSS SECcurity GPC system equipped with an Agilent 1260 isopump, 1260 RI detector, inline vacuum degasser and THF (HPLC grade) as mobile phase. The mobile phase is mixed with toluene as internal standard having 2 mg/mL for the samples. Operating conditions were solvent THF; solute PLA and polystyrenes. It was furthermore measured at 40 °C on a PSS SDV column (1000 Angstrom porosity, 10µm particle size, up to 1.000 kDa molecular weight possible). This system was calibrated against polystyrene.

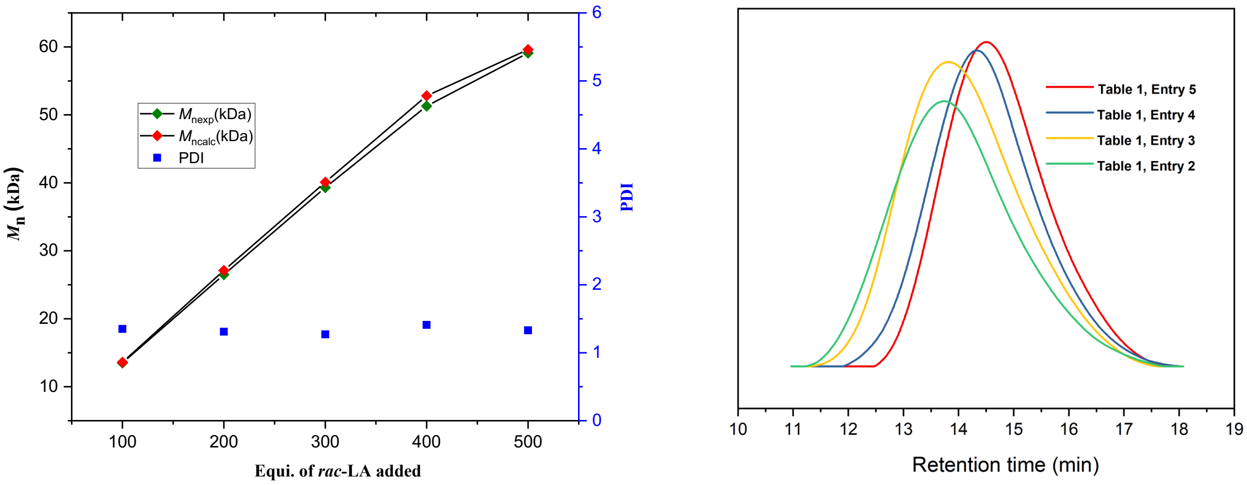

Figure S41. Left: Plots of observed and calculated PLA M_n and molecular weight distribution as functions of added monomer (*rac*-LA) with respect to catalyst **4a** (M_n = number averaged molecular weight, PDI = polydispersity index). All reactions were carried out at 60 °C in toluene, and conversion to polymer samples was >80%. Right: Chromatograms of PLA formed for different *rac*-LA : **4a** ratios (X : 1, X = 200, 300, 400, 500).

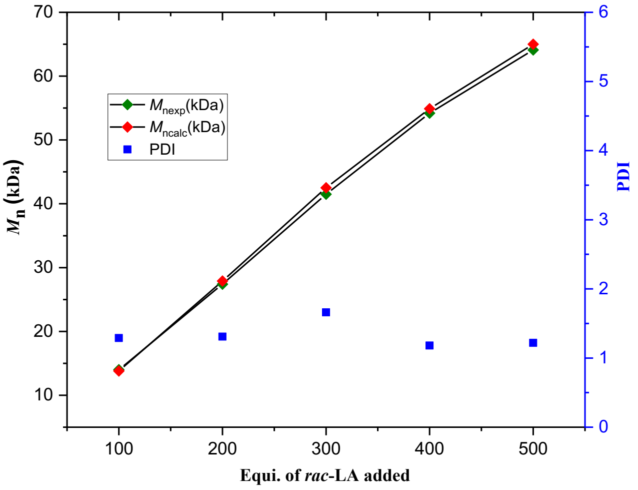

Figure S42. Plots of observed and calculated PLA M_n and molecular weight distribution as functions of added monomer (*rac*-LA) with respect to catalyst **4b** (M_n = number averaged molecular weight, PDI = polydispersity index). All reactions were carried out at 60 °C in toluene, and conversion to polymer samples was >80%.

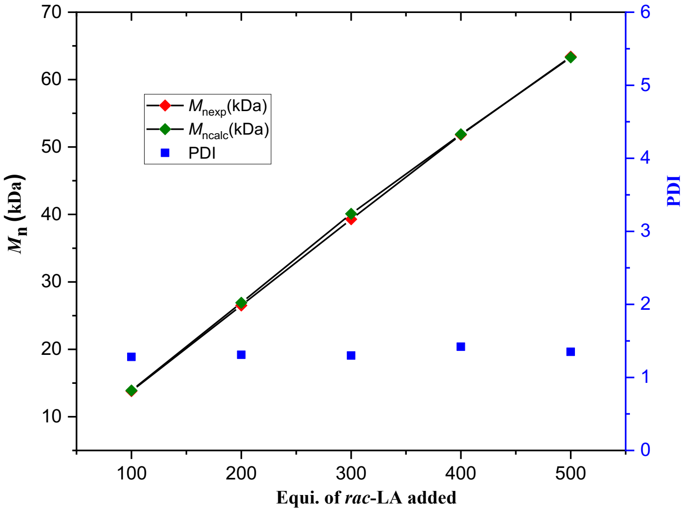

Figure S43. Plots of observed and calculated PLA M_n and molecular weight distribution as functions of added monomer (*rac*-LA) with respect to catalyst **4c** (M_n = number averaged molecular weight, PDI = polydispersity index). All reactions were carried out at 60 °C in toluene, and conversion to polymer samples was >80%.

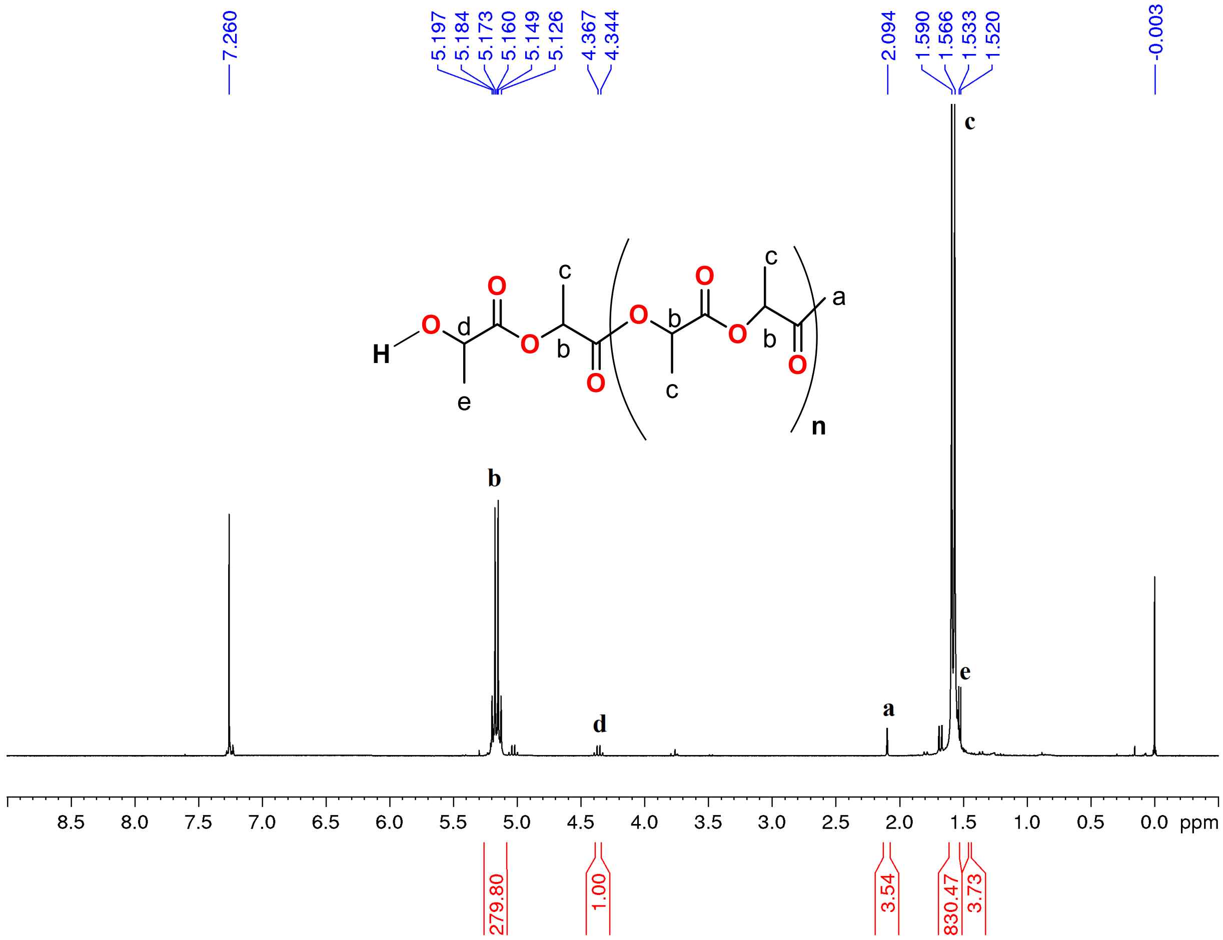

Figure S44. ^1H NMR spectrum of PLA prepared by catalyst **4a** ($[LA]_0/[M]$ = 300:1) in $CDCl_3$ with 91% conversion.

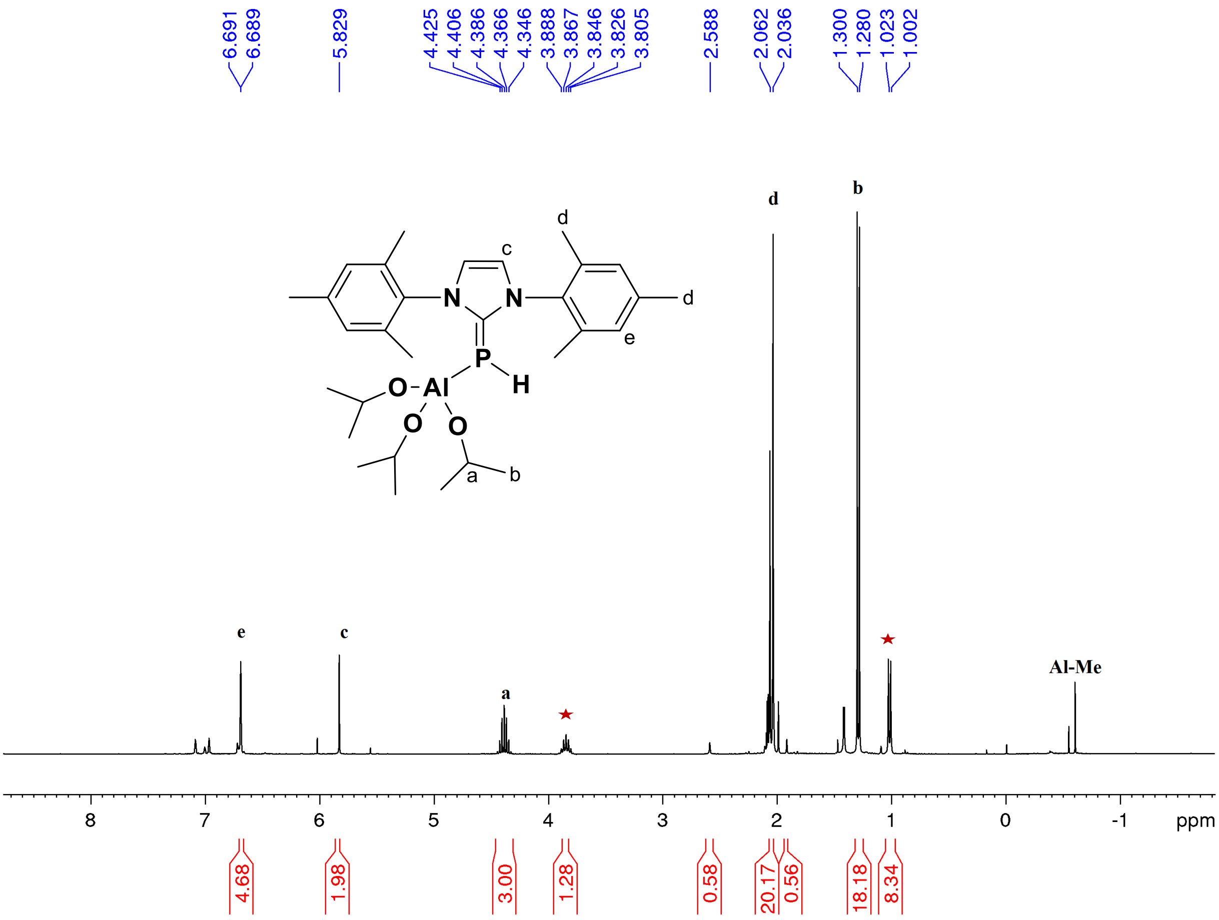

Figure S45. ^{1}H NMR spectrum of [{(IMes)PH}Al{OCH(CH_3)$_2$}$_3$] in toluene-d_8 (★excess isopropanol).

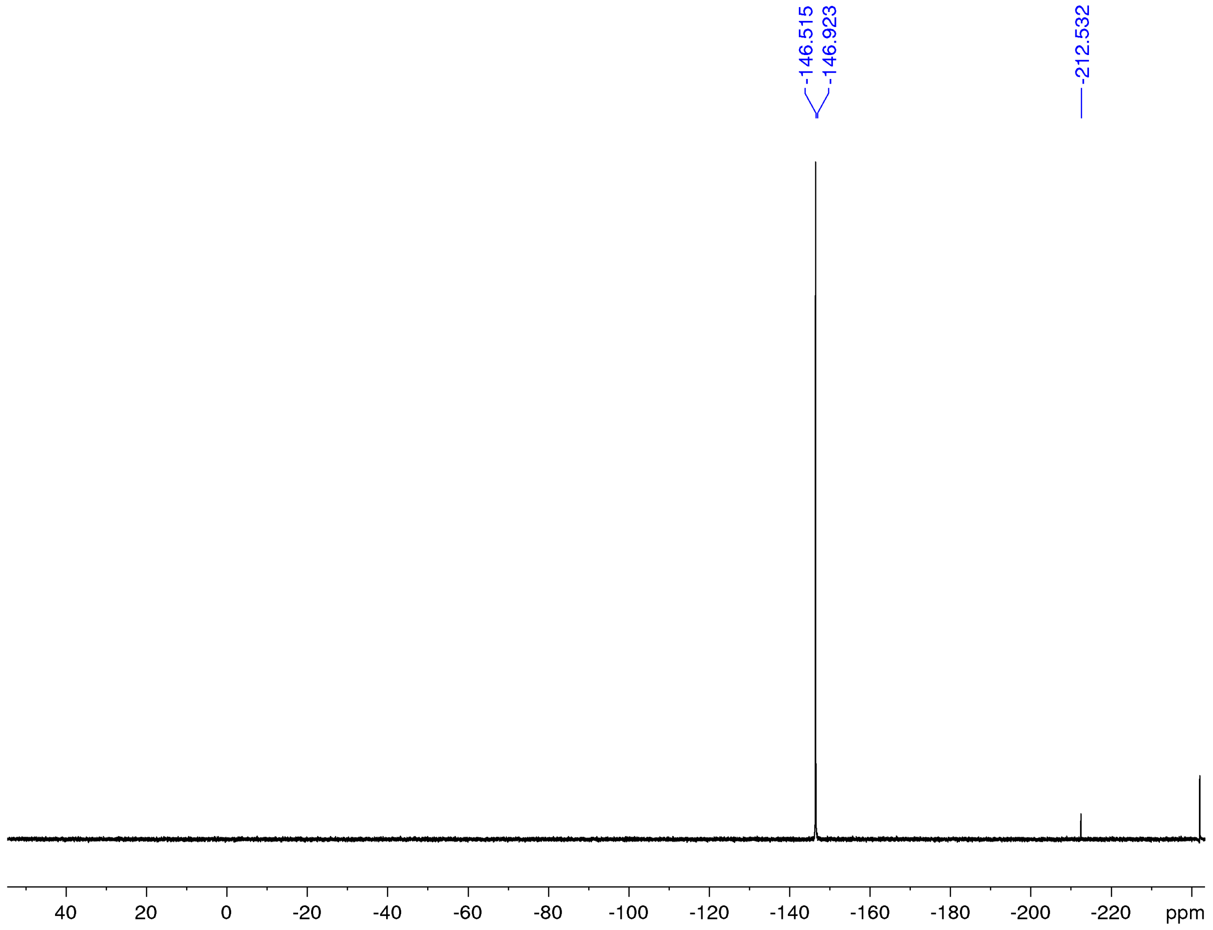

Figure 46. $^{31}P\{^{1}H\}$ NMR spectrum of [{(IMes)PH}Al{OCH(CH_3)$_2$}$_3$] in toluene-d_8.

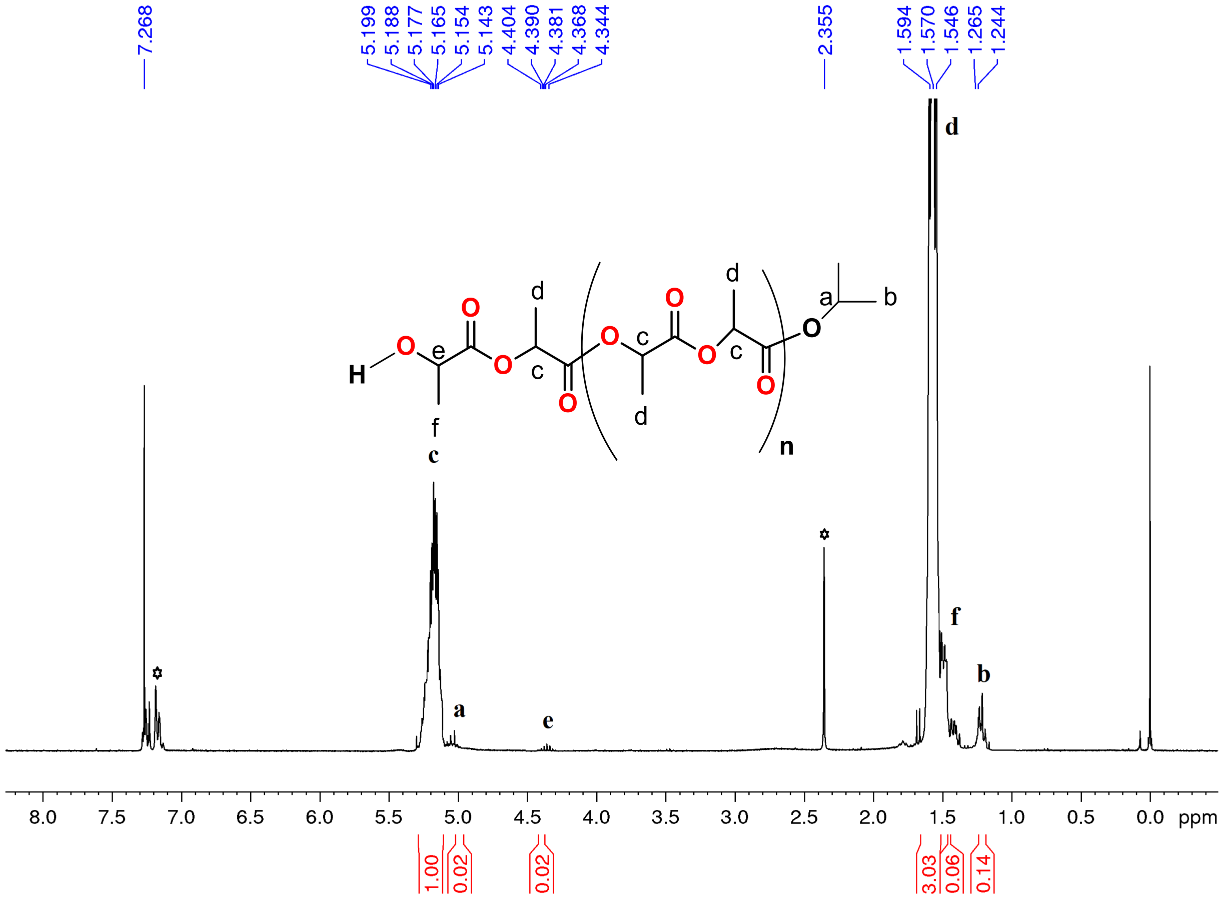

Figure S47. ^{1}H NMR spectrum of PLA prepared by catalyst **4b** ($[LA]_0/[Cat]_0/[^iPrOH]_0$ = 50:1:5) in $CDCl_3$ with 99% conversion (the asterisks mark residual toluene).

(S5) Kinetic studies

Details of the kinetic studies for ROP of *rac*-LA.

[{(IDipp)PH}AlMe$_3$] (4a) as catalyst.

A typical kinetic study was conducted to establish the reaction order with respect to monomer and [{(IDipp)PH}AlMe$_3$] (**4a**) as catalyst. For LA polymerization, *rac*-LA (0.0288 g, 0.20 mmol) was added to a solution of **4a** (0.002, 0.004, 0.006, 0.008, 0.01 mmol) in C_6D_6 (1 mL), respectively. Tetramethylsilane was added as an internal standard. The solution was heated in the NMR tube at 60 °C. At the indicated time intervals; the sample was analyzed by ^{1}H NMR. The *rac*-LA concentration [LA] was determined by integrating the quartet methine peak of LA at 3.7 ppm and the PLA concentration by integrating the broad methine peak at 5.09–5.20 ppm. As expected, the linear fit of data to plots of $[LA]_0/[LA]_t$ vs. time (t) indicate first order dependence on monomer concentration (Figure S48, Table S1). Thus, the rate expression can be written as $-d[LA]/dt = k_p[LA]^1[\{(IDipp)PH\}AlMe_3]^x = k_{obs}[LA]^1$, where $k_{obs} = k_p[\{(IDipp)PH\}AlMe_3]^x$. A plot of ($k_{obs}$) vs. [{(IDipp)PH}AlMe$_3$] (Figure S49, Table S2) is linear,

indicating the first order dependence on [{(IDipp)PH}$AlMe_3$] concentration and affording a second-order rate constant of propagation (k_p), which is 20.1 ± 1.2 $M^{-1}h^{-1}$.

Table S1. Data for first order kinetic plots of *rac*-LA polymerizations in C_6D_6 (1 mL) with different concentrations of **4a**.

Entry	[LA]/[Cat]	Time (h:m)	Conversion[a]	[PLA]	[*rac*- LA]$_t$	([LA]$_0$ /[LA]$_t$)	ln([LA]$_0$/ [LA]$_t$)
1	100/1	00.00	0%	0.00	0.20	0.00	0
2	100/1	01.00	8.5%	0.017	0.183	1.09	0.09
3	100/1	03.00	16%	0.032	0.168	1.19	0.18
4	100/1	05.00	18%	0.043	0.157	1.27	0.24
5	100/1	07.00	22%	0.055	0.145	1.38	0.32
6	100/1	09.00	33%	0.066	0.134	1.49	0.4
7	100/1	11.00	38%	0.076	0.124	1.61	0.48
8	100/1	13.00	43%	0.086	0.114	1.75	0.56
9	100/2	00.00	0%	0.00	0.20	0.00	0
10	100/2	01.00	18%	0.035	0.165	1.21	0.19
11	100/2	03.00	26.5%	0.053	0.147	1.36	0.31
12	100/2	05.00	34%	0.068	0.132	1.52	0.42
13	100/2	07.00	43%	0.085	0.115	1.73	0.55
14	100/2	09.00	48%	0.098	0.102	1.95	0.67
15	100/2	11.00	56%	0.112	0.088	2.27	0.82
16	100/2	13.00	63%	0.126	0.074	2.69	0.99
17	100/3	00.00	0%	0.00	0.20	0.00	0
18	100/3	01.00	25%	0.050	0.150	1.33	0.29
19	100/3	03.00	36%	0.072	0.128	1.56	0.45
20	100/3	05.00	47%	0.094	0.106	1.88	0.63
21	100/3	07.00	56%	0.112	0.088	2.27	0.82
22	100/3	09.00	63%	0.126	0.074	2.70	0.99
23	100/3	11.00	70%	0.140	0.060	3.32	1.2
24	100/3	13.00	77%	0.154	0.046	4.31	1.46
25	100/4	00.00	0%	0.00	0.20	0.00	0
26	100/4	01.00	29%	0.058	0.142	1.40	0.34
27	100/4	03.00	46%	0.092	0.108	1.84	0.61
28	100/4	05.00	59%	0.117	0.083	2.41	0.88
29	100/4	07.00	67%	0.134	0.066	3.03	1.11
30	100/4	09.00	75%	0.150	0.050	3.95	1.38
31	100/4	11.00	81.5%	0.163	0.037	5.37	1.68
32	100/4	13.00	86.5%	0.173	0.027	7.39	2
33	100/5	00.00	0%	0.00	0.20	0.00	0
34	100/5	01.00	39.5%	0.079	0.121	1.65	0.5
35	100/5	03.00	57.5%	0.115	0.085	2.36	0.86
36	100/5	05.00	71%	0.142	0.058	3.42	1.23
37	100/5	07.00	81%	0.162	0.038	5.26	1.66
38	100/5	09.00	86%	0.176	0.024	8.17	2.10
39	100/5	11.00	91%	0.182	0.018	11.1	2.41
40	100/5	13.00	94%	0.188	0.012	16.4	2.8

[a] Obtained from ^1H NMR analysis.

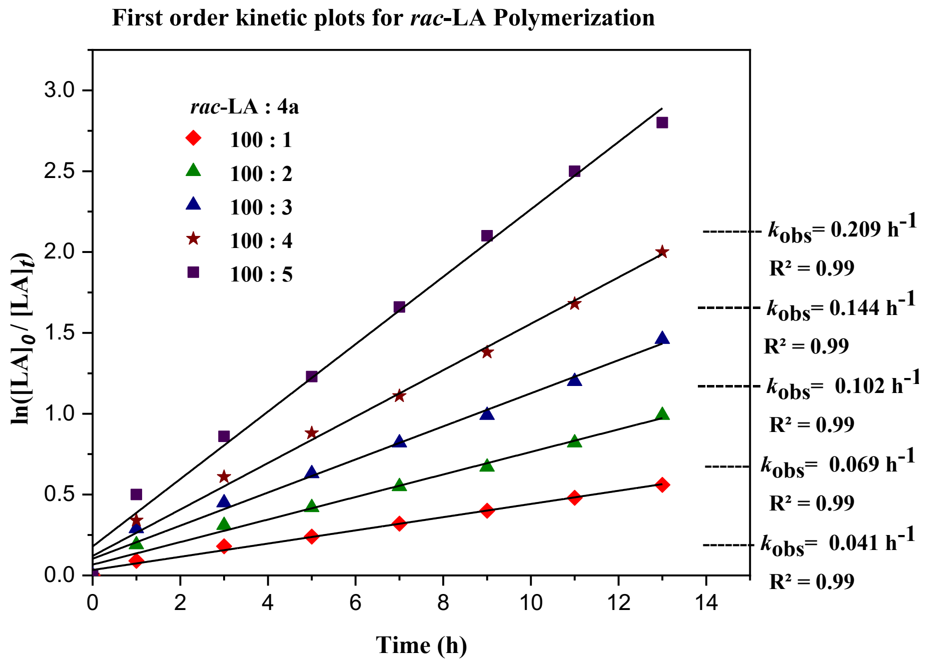

Figure S48. First order kinetic plots for *rac*-LA polymerization in C_6D_6 (1 mL) with different concentrations of [{(IDipp)PH}$AlMe_3$] (**4a**) at 60 °C.

Table S2. Data for kinetic plot of k_{obs} vs [{(IDipp)PH}$AlMe_3$](**4a**) for the polymerization of *rac*-LA with [LA] = 0.20 M in C_6D_6 (1 mL) at 60 °C.

Entry	[**4a**] (M)	k_{obs} (h^{-1})
1	0	0
2	0.002	0.041(1)
3	0.004	0.069(3)
4	0.006	0.102(4)
5	0.008	0.144(5)
6	0.010	0.209(7)

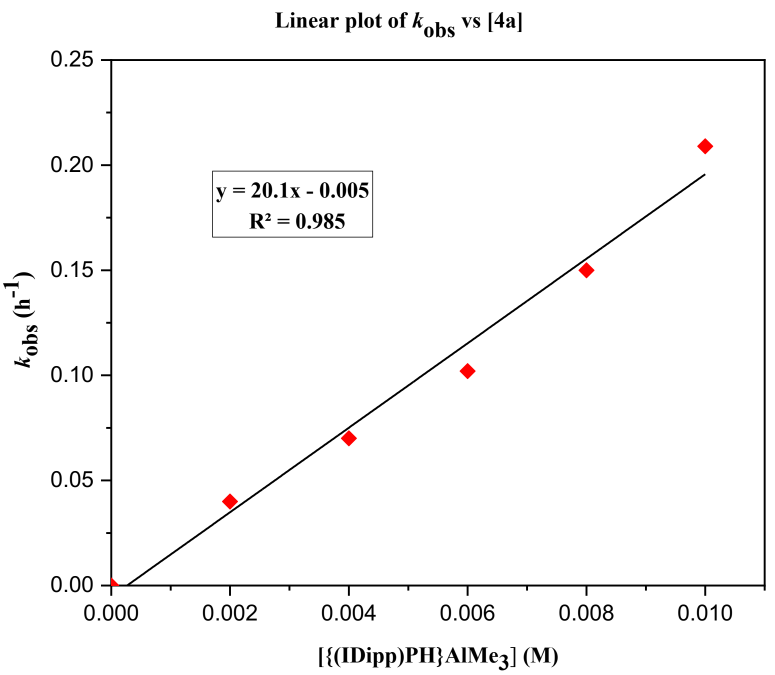

Figure S49. Kinetic plot of k_{obs} vs [{(IDipp)PH}AlMe$_3$] (**4a**) for the polymerization of *rac*-LA with [LA] = 0.20 M in C_6D_6 (1 mL) at 60 °C.

[{(IMes)PH}AlMe$_3$] (4b) as a catalyst.

A typical kinetics study was conducted to establish the reaction order with respect to monomer and [{(IMes)PH}AlMe$_3$] (**4b**) as catalyst. For LA polymerization, *rac*-LA (0.0288 g, 0.2 mmol) was added to a solution of **4b** (0.002, 0.004, 0.006, 0.008, 0.01 mmol) in C_6D_6 (1 mL), respectively. Tetramethylsilane was added as an internal standard. The solution was heated in the NMR tube at 60 °C. At the indicated time intervals; the sample was analyzed by ^{1}H NMR. The *rac*-LA concentration [LA] was determined by integrating the quartet methine peak of LA at 3.7 ppm and the PLA concentration by integrating the broad methine peak at 5.09–5.20 ppm. As expected, the linear fit of data to plots of $[LA]_0/[LA]_t$ vs. time (t) indicate first order dependence on monomer concentration (Figure S50, Table S3). Thus, the rate expression can be written as $-d[LA]/dt = k_p[LA]^1[\{(IMes)PH\}AlMe_3]^x = k_{obs}[LA]^1$, where $k_{obs} = k_p[\{(IMes)PH\}AlMe_3]^x$. A plot of ($k_{obs}$) vs. [{(IMes)PH}AlMe$_3$] (Figure S51, Table S4) is linear, indicating first order dependence on [{(IMes)PH}AlMe$_3$] concentration and affording a second-order rate constant of propagation (k_p) which is 24.4 ± 1.7 M^{-1}h^{-1}.

Table S3. Data for first order kinetic plots of *rac*-LA polymerizations in C_6D_6 (1 mL) with different concentrations of **4b**.

Entry	[LA]/[Cat]	Time (h:m)	Conversion[a]	[PLA]	[*rac*- LA]$_t$	([LA]$_0$/ [LA]$_t$)	ln([LA]$_0$/ [LA]$_t$)
1	100/1	00.00	0%	0.00	0.20	0.0	0
2	100/1	01.00	10%	0.021	0.179	1.12	0.11
3	100/1	02.00	19%	0.038	0.162	1.23	0.21
4	100/1	04.00	26.5%	0.053	0.147	1.36	0.31
5	100/1	06.00	36%	0.072	0.127	1.57	0.45
6	100/1	08.00	45%	0.089	0.111	1.80	0.59
7	100/1	10.00	51%	0.101	0.098	2.03	0.71

8	100/1	12.00	75%	0.115	0.085	2.63	0.86
9	100/2	00.00	0%	0.00	0.20	0.00	0
10	100/2	01.00	22.5%	0.045	0.155	1.29	0.25
11	100/2	02.00	35%	0.070	0.130	1.54	0.43
12	100/2	04.00	50.5%	0.101	0.099	2.01	0.7
13	100/2	06.00	59%	0.118	0.082	2.43	0.89
14	100/2	08.00	66.5%	0.133	0.067	2.98	1.09
15	100/2	10.00	74%	0.148	0.052	3.86	1.35
16	100/2	12.00	80%	0.160	0.040	4.95	1.6
17	100/3	00.00	0%	0.00	0.20	0.00	0
18	100/3	01.00	25.5%	0.056	0.144	1.38	0.32
19	100/3	02.00	39%	0.078	0.121	1.65	0.50
20	100/3	04.00	55%	0.110	0.090	2.22	0.8
21	100/3	06.00	67%	0.134	0.066	3.01	1.1
22	100/3	08.00	75.5%	0.151	0.049	4.05	1.4
23	100/3	10.00	81.5%	0.163	0.037	5.37	1.68
24	100/3	12.00	86.5 %	0.173	0.027	7.53	2.02
25	100/4	00.00	0%	0.00	0.20	0.00	0
26	100/4	01.00	28.5%	0.057	0.143	1.39	0.34
27	100/4	02.00	46%	0.091	0.109	1.84	0.61
28	100/4	04.00	63.5%	0.127	0.072	2.77	1.02
29	100/4	06.00	74%	0.148	0.052	3.82	1.34
30	100/4	08.00	81.5%	0.163	0.037	5.47	1.7
31	100/4	10.00	87%	0.174	0.026	7.76	2.05
32	100/4	12.00	89%	0.178	0.022	9.09	2.20
33	100/5	00.00	0%	0.00	0.20	0.00	0
34	100/5	01.00	49%	0.098	0.102	1.73	0.55
35	100/5	02.00	58%	0.120	0.083	2.41	0.88
36	100/5	04.00	79%	0.158	0.042	4.48	1.5
37	100/5	06.00	81%	0.172	0.028	7.09	1.96
38	100/5	08.00	92%	0.182	0.018	11.24	2.42
39	100/5	10.00	93%	0.186	0.014	14.28	2.65
40	100/5	12.00	96%	0.193	0.007	27.11	3.3

[a] Obtained from ^{1}H NMR analysis.

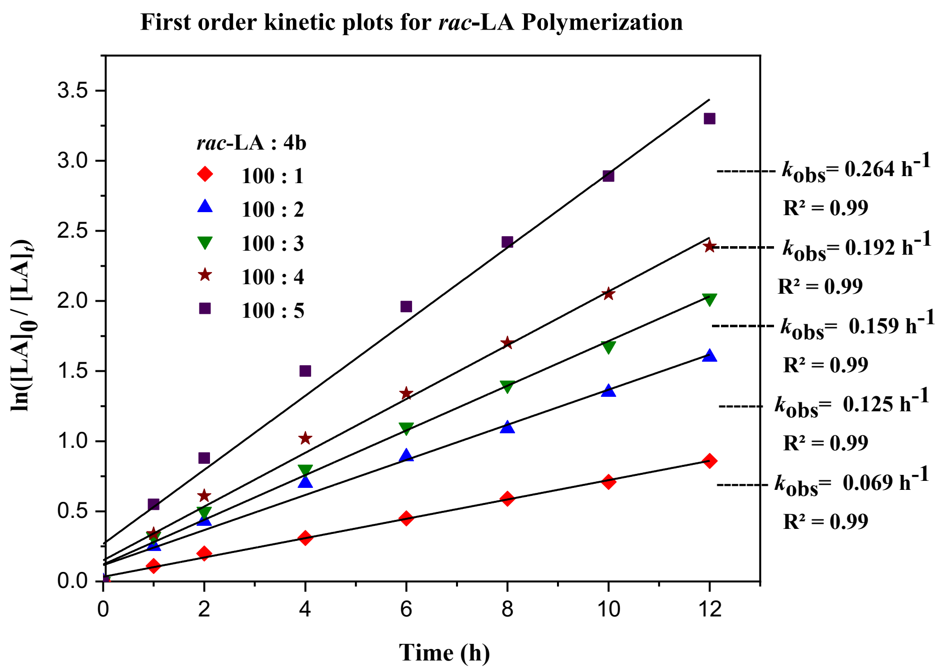

Figure S50. First order kinetic plots for *rac*-LA polymerizations in C_6D_6 (1 mL) with different concentrations of [{(IMes)PH}AlMe$_3$] (**4b**) at 60 °C.

Table S4. Data for kinetic plot of k_{obs} vs [{(IMes)PH}AlMe$_3$] (**4b**) for the polymerization of *rac*-LA with [LA] = 0.20 M in C_6D_6 (1 mL) at 60 °C.

Entry	[**4b**] (M)	k_{obs} (h^{-1})
1	0	0
2	0.002	0.07(1)
3	0.004	0.125(5)
4	0.006	0.159(5)
5	0.008	0.192(7)
6	0.010	0.264(6)

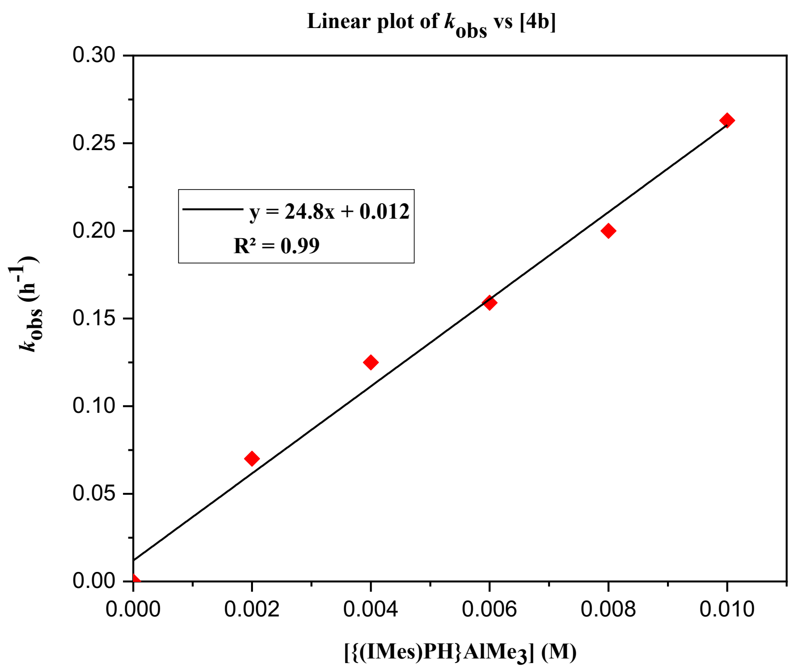

Figure S51. Kinetic plot of k_{obs} vs [{(IMes)PH}AlMe$_3$](**4b**) for the polymerization of *rac*-LA with [LA] = 0.20 M in C_6D_6 (1 mL) at 60 °C.

[{(^{Me}IMes)PH}(AlMe$_3$)$_2$] (4c) as a catalyst.

A typical kinetics study was conducted to establish the reaction order with respect to monomer and [{(^{Me}IMes)PH}(AlMe$_3$)$_2$] (**4c**) as catalyst. For LA polymerization, *rac*-LA (0.0288 g, 0.2 mmol) was added to a solution of **4c** (0.002, 0.004, 0.006, 0.008, 0.01 M) in C_6D_6 (1 mL), respectively. The solution was heated in the NMR tube at 60 °C. At the indicated time intervals; the sample was analyzed by ^{1}H NMR. The *rac*-LA concentration [LA] was determined by integrating the quartet methine peak of LA at 3.7 ppm and the PLA concentration by integrating the broad methine peak at 5.09–5.20 ppm. As expected, the linear fit of data to plots of $[LA]_0/[LA]_t$ vs. time (t) indicate first order dependence on monomer concentration (Figure S52, Table S5). Thus, the rate expression can be written as $-d[LA]/dt = k_p[LA]^1[\{(^{Me}IMes)PH\}(AlMe_3)_2]^x = k_{obs}[LA]^1$, where $k_{obs} = k_p[\{(^{Me}IMes)PH\}(AlMe_3)_2]^x$. A plot of ($k_{obs}$) vs. [{(^{Me}IMes)PH}(AlMe$_3$)$_2$] (Figure S53, Table S6) is linear, indicating first order dependence on [{(^{Me}IMes)PH}(AlMe$_3$)$_2$] concentration and affording a second-order rate constant of propagation (k_p) which is 21.28 ± 0.8 M^{-1}h^{-1}.

Table S5. Data for first order kinetic plots of *rac*-LA polymerizations in C_6D_6 (1 mL) with different concentrations of **4c**.

Entry	[LA]/[Cat]	Time (h:m)	Conversion[a]	[PLA]	[*rac*- LA]$_t$	([LA]$_0$ /[LA]$_t$)	ln([LA]$_0$/ [LA]$_t$)
1	100/1	00.00	0%	0.00	0.20	0.00	0
2	100/1	01.00	10%	0.020	0.180	1.11	0.1
3	100/1	03.00	18%	0.036	0.163	1.22	0.2
4	100/1	05.00	26.5%	0.053	0.147	1.36	0.31
5	100/1	07.00	39%	0.078	0.122	1.64	0.4
6	100/1	09.00	39%	0.078	0.122	1.64	0.51
7	100/1	11.00	50.5%	0.101	0.099	2.01	0.7

8	100/1	13.00	55%	0.110	0.090	2.22	0.8
9	100/2	00.00	0%	0.00	0.20	0.00	0
10	100/2	01.00	20%	0.04	0.16	1.25	0.23
11	100/2	03.00	36%	0.072	0.128	1.56	0.45
12	100/2	05.00	41.5%	0.083	0.117	1.71	0.54
13	100/2	07.00	52.5%	0.105	0.095	2.11	0.75
14	100/2	09.00	60%	0.120	0.080	2.50	0.92
15	100/2	11.00	66.5%	0.133	0.066	3.00	1.1
16	100/2	13.00	74%	0.148	0.052	3.85	1.35
17	100/3	00.00	0%	0.00	0.20	0.00	0
18	100/3	01.00	23.5%	0.047	0.152	1.31	0.27
19	100/3	03.00	39.5%	0.079	0.121	1.65	0.5
20	100/3	05.00	48%	0.096	0.104	1.92	0.65
21	100/3	07.00	59.5%	0.119	0.081	2.46	0.9
22	100/3	09.00	70%	0.140	0.060	3.32	1.2
23	100/3	11.00	77.5%	0.155	0.045	4.48	1.5
24	100/3	13.00	83.5%	0.167	0.033	6.05	1.8
25	100/4	00.00	0%	0.00	0.20	0.00	0
26	100/4	01.00	26.5%	0.053	0.147	1.36	0.31
27	100/4	03.00	46.5%	0.093	0.107	1.86	0.625
28	100/4	05.00	61%	0.122	0.078	2.58	0.95
29	100/4	07.00	66%	0.132	0.068	2.94	1.33
30	100/4	09.00	74%	0.147	0.053	3.78	1.75
31	100/4	11.00	87.5%	0.175	0.025	8.16	2.1
32	100/4	13.00	90%	0.18	0.02	10.0	2.30
33	100/5	00.00	0%	0.00	0.20	0.00	0
34	100/5	01.00	29%	0.058	0.142	1.40	0.34
35	100/5	03.00	55%	0.110	0.090	2.22	0.8
36	100/5	05.00	70%	0.140	0.060	3.32	1.2
37	100/5	07.00	80%	0.160	0.040	4.95	1.6
38	100/5	09.00	86%	0.176	0.024	8.17	2.10
39	100/5	11.00	90%	0.18	0.016	12.18	2.5
40	100/5	13.00	95%	0.190	0.009	20.08	3

[a] Obtained from ^{1}H NMR analysis.

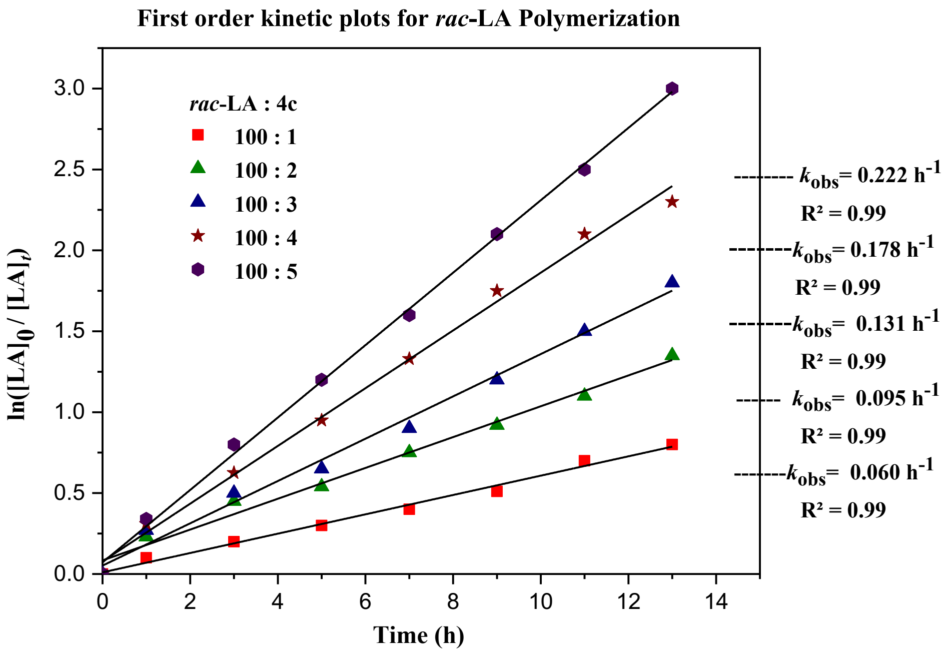

Figure S52. First order kinetic plots for *rac*-LA polymerizations in C_6D_6 (1 mL) with different concentrations of [{(^{Me}IMes)PH}($AlMe_3$)$_2$] (**4c**) at 60 °C.

Table S6. Data for kinetic plot of k_{obs} vs [{(^{Me}IMes)PH}($AlMe_3$)$_2$] (**4c**) for the polymerization of *rac*-LA with [LA] = 0.20 M in C_6D_6 (1 mL) at 60 °C.

Entry	[**4c**] (M)	k_{obs} (h^{-1})
1	0	0
2	0.002	0.060(2)
3	0.004	0.095(4)
4	0.006	0.131(5)
5	0.008	0.178(5)
6	0.010	0.222(3)

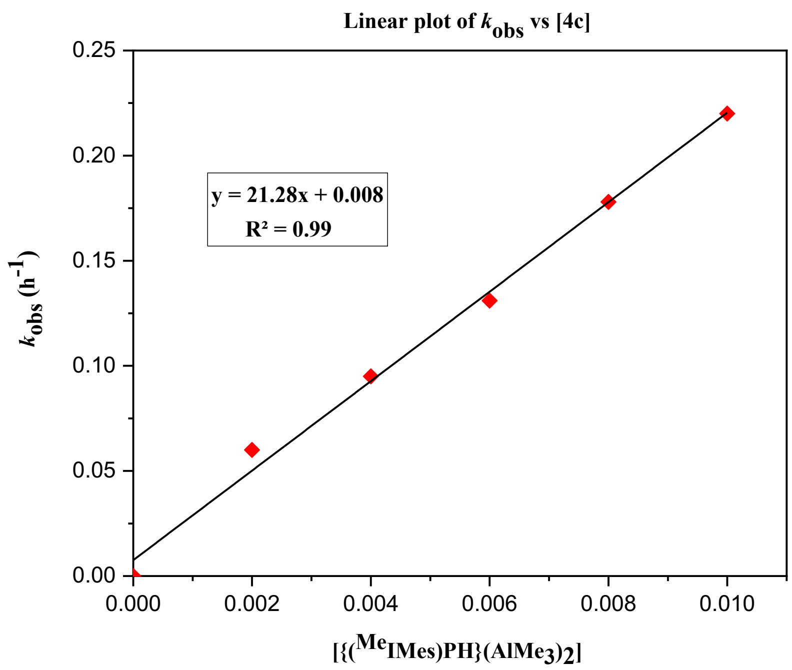

Figure S53. Kinetic plot of k_{obs} vs [{(^{Me}IMes)PH}(AlMe$_3$)$_2$] (**4c**) for the polymerization of *rac*-LA with [LA] = 0.20 M in C_6D_6 (1 mL) at 60 °C.

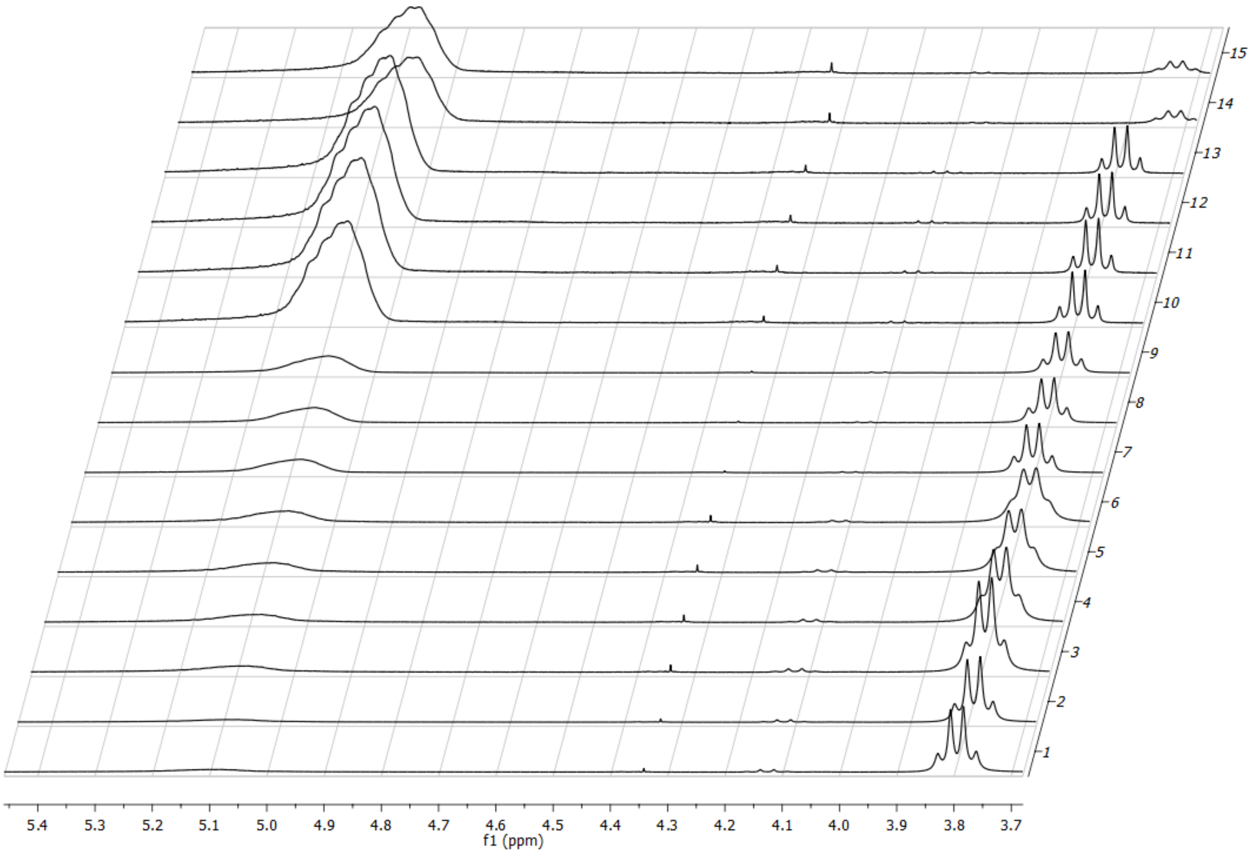

Figure S54. Stacked ^{1}H NMR spectra for the polymerization of *rac*-LA with [LA] = 0.20 M in C_6D_6 (1 mL) at 60 °C catalyzed by [{(IMes)PH}AlMe$_3$] (**4b**) (0.002 M); the NMR spectra were recorded every hour.

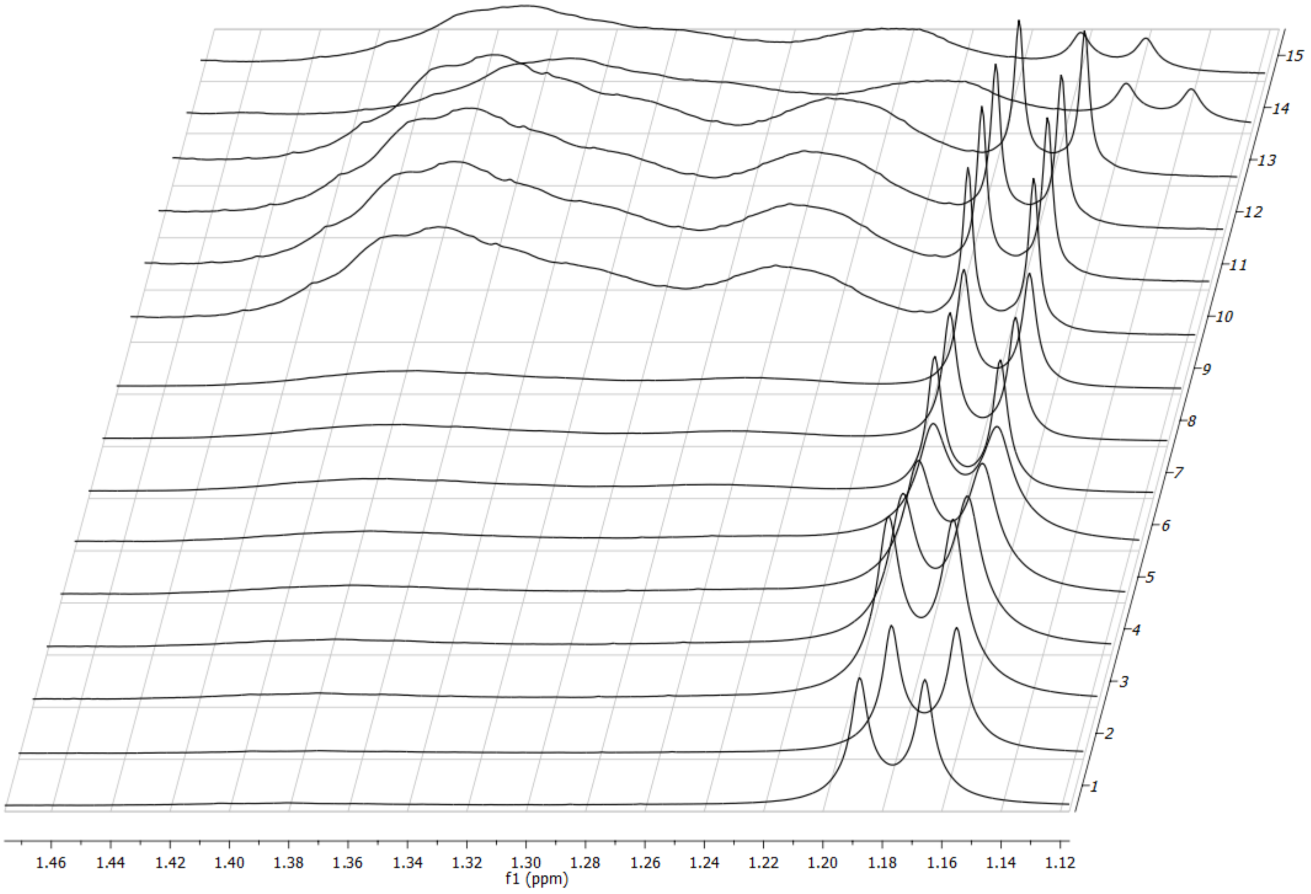

Figure S55. Stacked ^{1}H NMR spectra for the polymerization of *rac*-LA with [LA] = 0.20 M in C_6D_6 (1 mL) at 60 °C catalyzed by [{(IMes)PH}$AlMe_3$] (**4b**) (0.002 M); the NMR spectra were recorded every hour.

Table S7: Rate constants for polymerization of *rac*-LA with various concentrations of [{(IDipp)PH}$AlMe_3$] (**4a**), [{(IMes)PH}$AlMe_3$] (**4b**), [{(^{Me}IMes)PH}$(AlMe_3)_2$] (**4c**) as a catalyst.

Entry	Catalyst	*rac*-LA : catalyst	k_{obs} (h^{-1})
1	[{(IDipp)PH}$AlMe_3$] (**4a**)	100 : 1	0.041(1)
2	[{(IMes)PH}$AlMe_3$] (**4b**)	100 : 1	0.069(1)
3	[{(^{Me}IMes)PH}$(AlMe_3)_2$] (**4c**)	100 : 1	0.060(2)
4	[{(IDipp)PH}$AlMe_3$] (**4a**)	100 : 2	0.069(3)
5	[{(IMes)PH}$AlMe_3$] (**4b**)	100 : 2	0.125(5)
6	[{(^{Me}IMes)PH}$(AlMe_3)_2$] (**4c**)	100 : 2	0.095(4)
7	[{(IDipp)PH}$AlMe_3$] (**4a**)	100 : 3	0.102(4)
8	[{(IMes)PH}$AlMe_3$] (**4b**)	100 : 3	0.159(5)
9	[{(^{Me}IMes)PH}$(AlMe_3)_2$] (**4c**)	100 : 3	0.131(5)
10	[{(IDipp)PH}$AlMe_3$] (**4a**)	100 : 4	0.144(5)
11	[{(IMes)PH}$AlMe_3$] (**4b**)	100 : 4	0.192(7)

12	[{(^{Me}IMes)PH}($AlMe_3$)$_2$] (**4c**)	100 : 4	0.178(5)
13	[{(IDipp)PH}$AlMe_3$] (**4a**)	100 : 5	0.209(7)
14	[{(IMes)PH}$AlMe_3$] (**4b**)	100 : 5	0.264(6)
15	[{(^{Me}IMes)PH}($AlMe_3$)$_2$] (**4c**)	100 : 1	0.222(3)

(S6) Thermodynamic Parameters

Eyring Equation

$$\ln\frac{k}{T} = \frac{-\Delta H^{\ddagger}}{R}\cdot\frac{1}{T} + \ln\frac{k_B}{h} + \frac{\Delta S^{\ddagger}}{R}$$

Arrhenius equation

$$ln\ k = -\frac{E_a}{RT} + ln\ A$$

[{(IDipp)PH}$AlMe_3$] (4a) as catalyst

The activation parameters for the ring-opening polymerization of *rac*-LA catalyzed by [{(IDipp)PH}$AlMe_3$] (**4a**) in C_6D_6 were found to be $\Delta H^{\ddagger}$ = 57.0(3) kJ mol^{-1} $\Delta S^{\ddagger}$ = –123.0(7) J mol^{-1}K^{-1} and ΔE_a = 59.93(3) kJmol^{-1}. These values were calculated from the temperature-dependent second order rate constants determined from k_p divided by [T] as provided in Table S8 and from the slope and intercept of Eyring and Arrhenius plots (Figure S57 and S58).

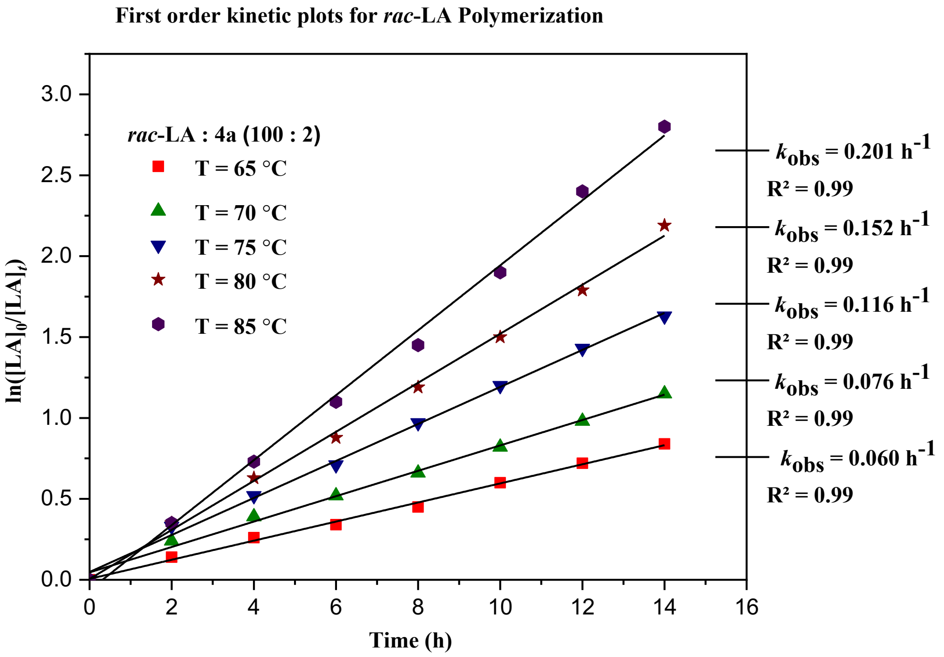

Figure S56. First order kinetic plots for *rac*-LA polymerizations in C_6D_6 (1 mL) with different temperatures using catalyst [{(IDipp)PH}$AlMe_3$] (**4a**).

Table S8. Data for Eyring and Arrhenius plots of ln(k_p/T) vs (1/T) and ln(k_p) vs (1/T) catalyzed by [{(IDipp)PH}AlMe$_3$] (**4a**) [reaction conditions: [LA] = 0.20 M and 0.004 M concentration of [{(IDipp)PH}AlMe$_3$] (**4a**) in C_6D_6 (1 mL)].

Entry	T in K	(1/T) (K^{-1})	K_p ($mol^{-1}L\ s^{-1}$)	lnk_p	ln(k_p/T)
1	338	0.00295	0.00416(1)	-5.48	-11.3
2	343	0.00292	0.00527(2)	-5.24	-11.08
3	348	0.00287	0.00805(2)	-4.82	-10.67
4	353	0.00283	0.01055(3)	-4.55	-10.41
5	358	0.00278	0.01395(5)	-4.27	-10.15

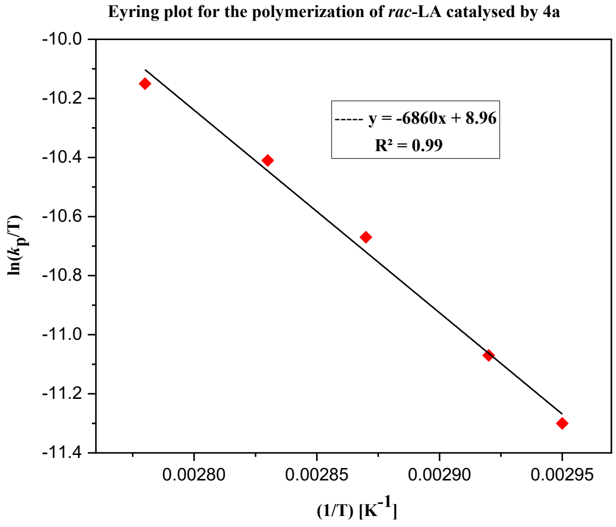

Figure S57. Eyring plot of ln(k_p/T) vs (1/T) for [{(IDipp)PH}AlMe$_3$] (**4a**) catalyzed ROP of *rac*-LA with [LA] = 0.20 M in C_6D_6 (1 mL) affording $\Delta H^‡$ = 57.0(3) kJmol^{-1} and $\Delta S^‡$ = –123.0(7) Jmol^{-1}K^{-1}.

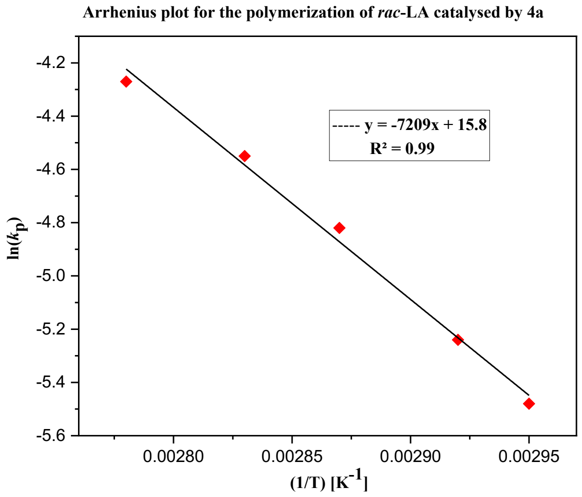

Figure S58. Arrhenius plot of ln(k_p) vs (1/T) for [{(IDipp)PH}AlMe$_3$] (**4a**) catalyzed ROP of *rac*-LA with [LA] = 0.20 M in C_6D_6 (1 mL) affording ΔE_a = 59.93 (3) kJ mol^{-1}.

[{(IMes)PH}AlMe$_3$] (4b) as catalyst

The activation parameters for the ring-opening polymerization of *rac*-LA catalyzed by [{(IMes)PH}AlMe$_3$] (**4b**) in C_6D_6 were found to be $\Delta H^{‡}$ = 47.3(2) kJ mol^{-1} $\Delta S^{‡}$ = –148.2(6) J mol^{-1}K^{-1} and ΔE_a = 50.2 (2) kJmol^{-1}. These values were calculated from the temperature-dependent second order rate constants determined from k_p divided by [T] as provided in Table S9 and from the slope and intercept of Eyring and Arrhenius plots (Figure S60 and S61).

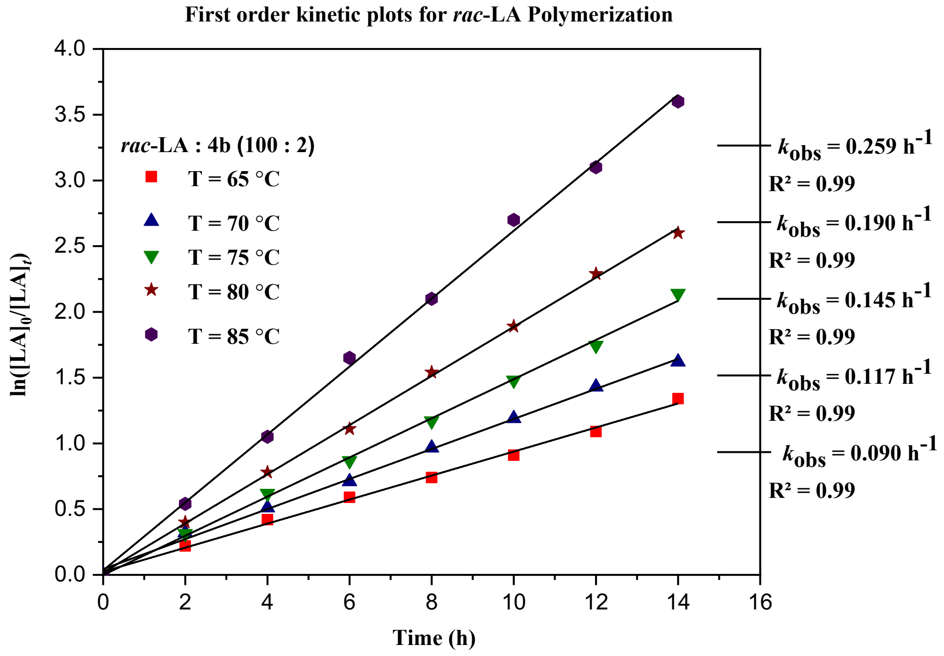

Figure S59. First order kinetic plots for *rac*-LA polymerizations in C_6D_6 (1 mL) with different temperatures catalyzed by [{(IMes)PH}AlMe$_3$] (**4b**).

Table S9. Data for Eyring and Arrhenius plots of ln(k_p/T) vs (1/T) and ln(k_p) vs (1/T) catalyzed by [{(IMes)PH}AlMe$_3$] (**4b**). [reaction conditions: [LA] = 0.20 M and 0.004 M concentration of [{(IMes)PH}AlMe$_3$] (**4b**) in C_6D_6 (1 mL)].

Entry	T in K	(1/T) (K^{-1})	K_p (mol^{-1}L s^{-1})	lnk_p	ln(k_p/T)
1	338	0.00295	0.00625(2)	-5.08	-10.90
2	343	0.00292	0.00813(2)	-4.81	-10.65
3	348	0.00287	0.01007(3)	-4.60	-10.45
4	353	0.00283	0.01319(2)	-4.32	-10.19
5	358	0.00278	0.01798(4)	-4.02	-9.9

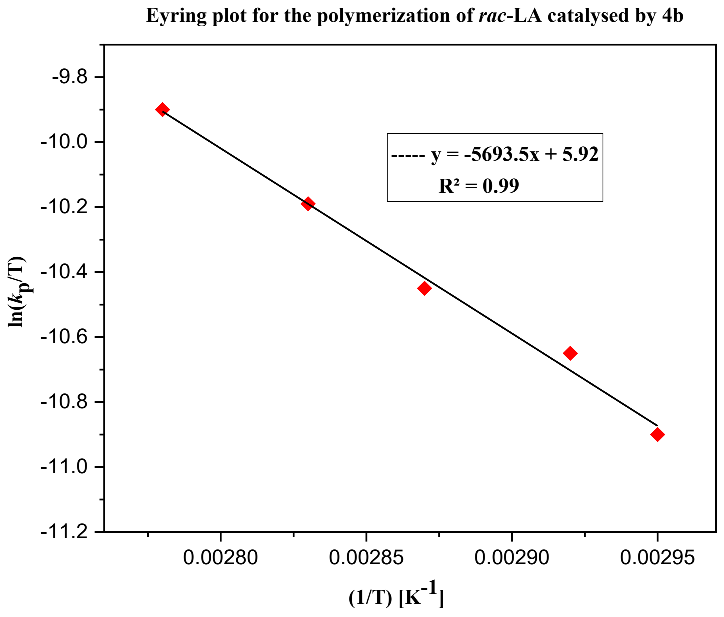

Figure S60. Eyring plot of ln(k_p/T) vs (1/T) for [{(IMes)PH}AlMe$_3$] (**4b**) catalyzed ROP of *rac*-LA with [LA] = 0.20 M in C_6D_6 (1 mL) affording $\Delta H^‡$ = 47.34(2) kJ mol^{-1} and $\Delta S^‡$ = –148.2(6) J mol^{-1}K^{-1}.

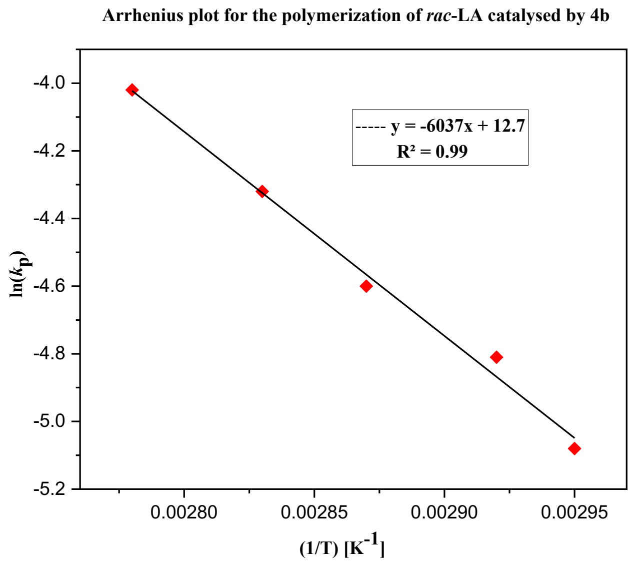

Figure S61. Arrhenius plot of ln(k_p) vs (1/T) for [{(IMes)PH}AlMe$_3$] (**4b**) catalyzed ROP of *rac*-LA with [LA] = 0.20 M in C_6D_6 (1 mL) affording ΔE_a = 50.2 (2) kJ mol^{-1}.

[{(^{Me}IMes)PH}(AlMe$_3$)$_2$] (4c) as catalyst.

The activation parameters for the ring-opening polymerization of *rac*-LA catalyzed by [{(^{Me}IMes)PH}(AlMe$_3$)$_2$] (**4c**) in C_6D_6 were found to be $\Delta H^‡$ = 51.80(3) kJ mol^{-1}, $\Delta S^‡$ = –138.3(1) J mol^{-1}K^{-1} and ΔE_a = 54.5(4) kJmol^{-1}. These values were calculated from the temperature-dependent second order rate constants determined from k_p divided by [T] as provided in Table S10 and from the slope and intercept of Eyring and Arrhenius plots (Figure S63 and S64).

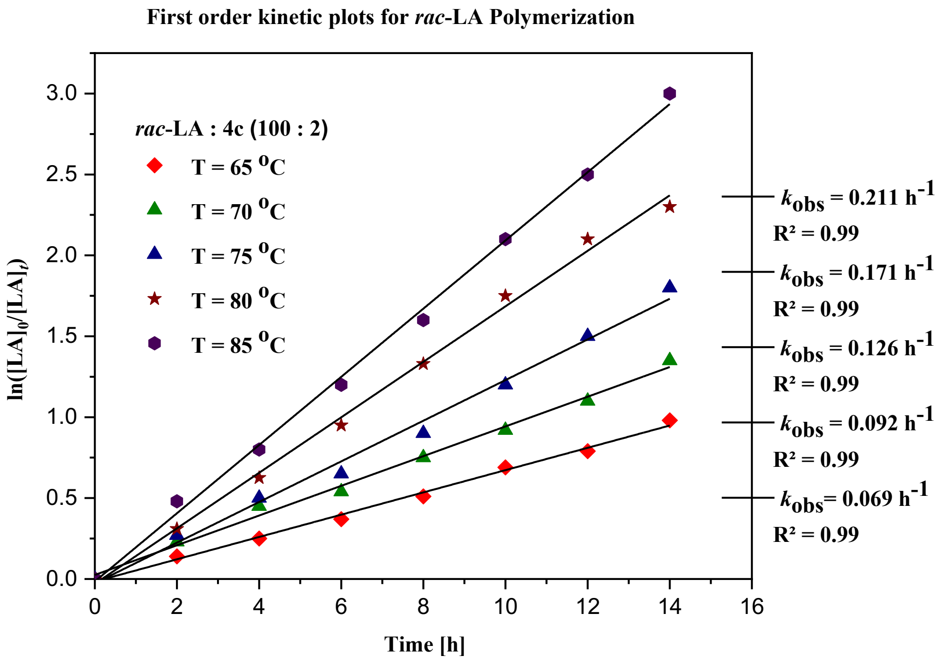

Figure S62. First order kinetic plots for *rac*-LA polymerizations in C_6D_6 (1 mL) with different temperatures catalyzed by [{(^{Me}IMes)PH}($AlMe_3$)$_2$] (**4c**).

Table S10. Data for Eyring and Arrhenius plots of ln(k_p/T) vs (1/T) and ln(k_p) vs (1/T) catalyzed by [{(^{Me}IMes)PH}($AlMe_3$)$_2$] (**4c**). [reaction condition: [LA] = 0.20 M and 0.004 M concentration of [{(^{Me}IMes)PH}($AlMe_3$)$_2$] (**4c**) in C_6D_6 (1 mL)].

Entry	T in K	(1/T) (K^{-1})	K_p ($mol^{-1}L\ s^{-1}$)	lnk_p	ln(k_p/T)
1	338	0.00295	0.00486(2)	-5.33	-11.15
2	343	0.00292	0.00638(3)	-5.05	-10.89
3	348	0.00287	0.00875(4)	-4.74	-10.59
4	353	0.00283	0.01187(4)	-4.43	-10.29
5	358	0.00278	0.01465(4)	-4.22	-10.10

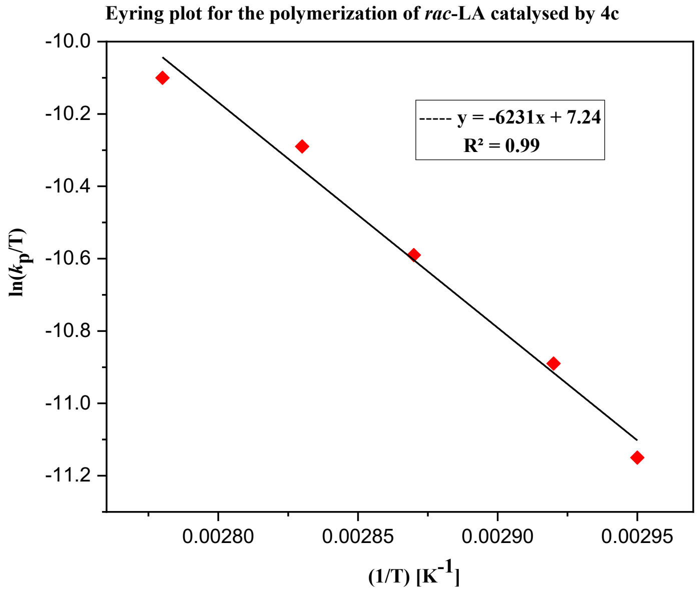

Figure S63. Eyring plot of ln(k_p/T) vs (1/T) for [{(^{Me}IMes)PH}($AlMe_3$)$_2$] (**4c**) catalyzed ROP of *rac*-LA with [LA] = 0.20 M in C_6D_6 (1 mL) affording Δ$H^‡$ = 51.80(3) kJ mol^{-1} and Δ$S^‡$ = –139.3(1) J mol^{-1}K^{-1}.

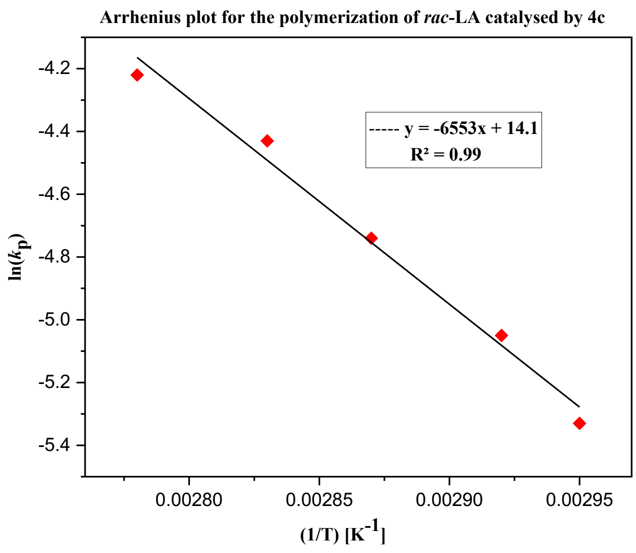

Figure S64. Arrhenius plot of ln(k_p) vs (1/T) for[{(^{Me}IMes)PH}($AlMe_3$)$_2$] (**4c**) catalyzed ROP of *rac*-LA with [LA] = 0.20 M in C_6D_6 (1 mL) affording ΔE_a = 54.5(4) kJ mol^{-1}.

Table S11: Comparison of thermodynamic parameters of *rac*-LA catalyzed by **(4a-c).**

Entry	Catalyst	Δ$H^‡$ (kJ/mol)	Δ$S^‡$ (J/mol.K)	E_a (kJ/mol)
1	[{(IDipp)PH}$AlMe_3$] (**4a**)	57.0(3)	–123.0(7)	59.93(3)
2	[{(IMes)PH}$AlMe_3$] (**4b**)	47.3(2)	–142.2(6)	50.2(2)
3	[{(^{Me}IMes)PH}($AlMe_3$)$_2$] (**4c**)	51.80(3)	–139.3(1)	54.50(4)

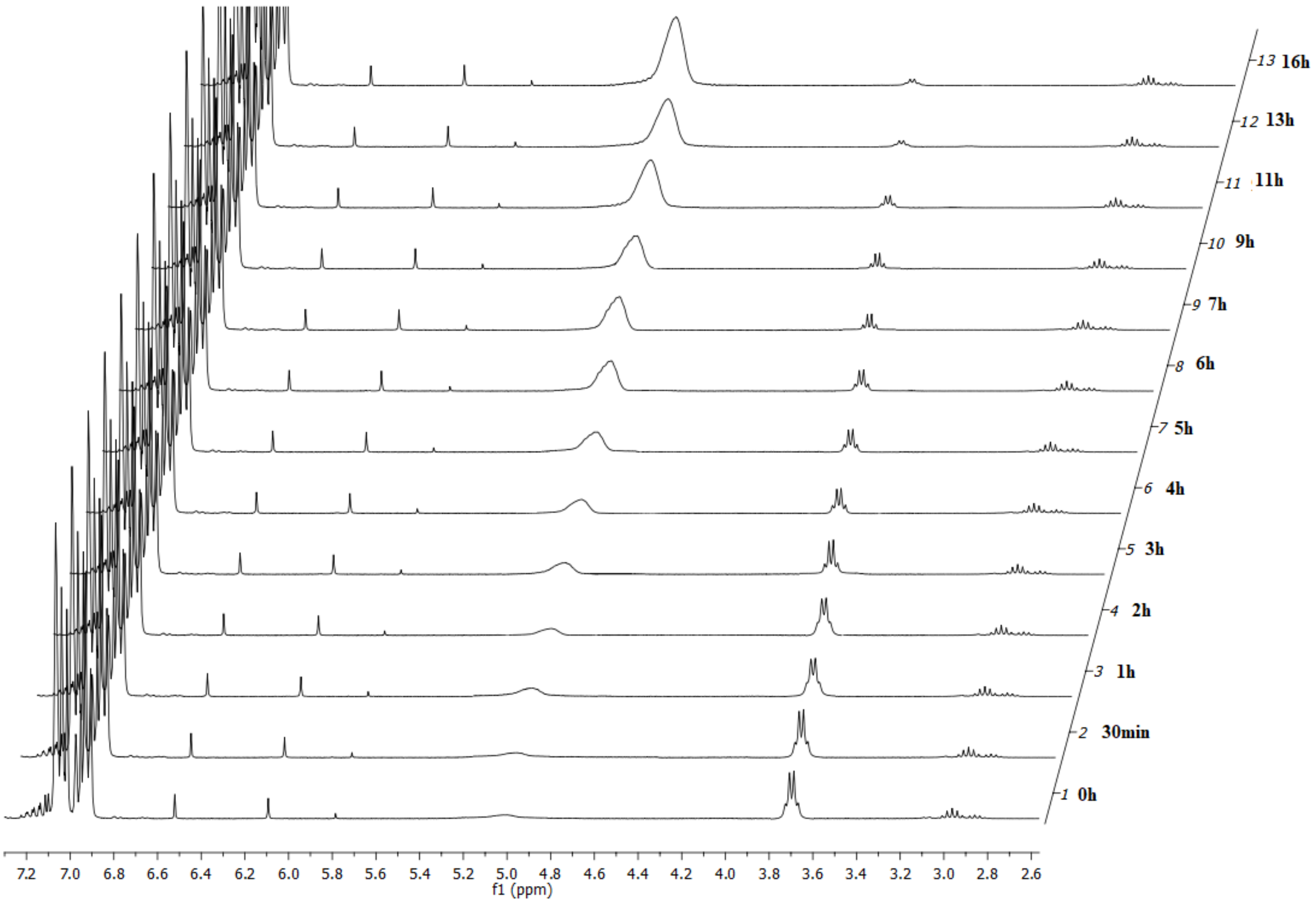

Figure S65. Stacked ^{1}H NMR spectra for the polymerization of *rac*-LA with [LA] = 0.20 M in C_6D_6 (1 mL) at 60 °C catalyzed by [{(IDipp)PH}$AlMe_3$] (**4a**) (0.008 M).

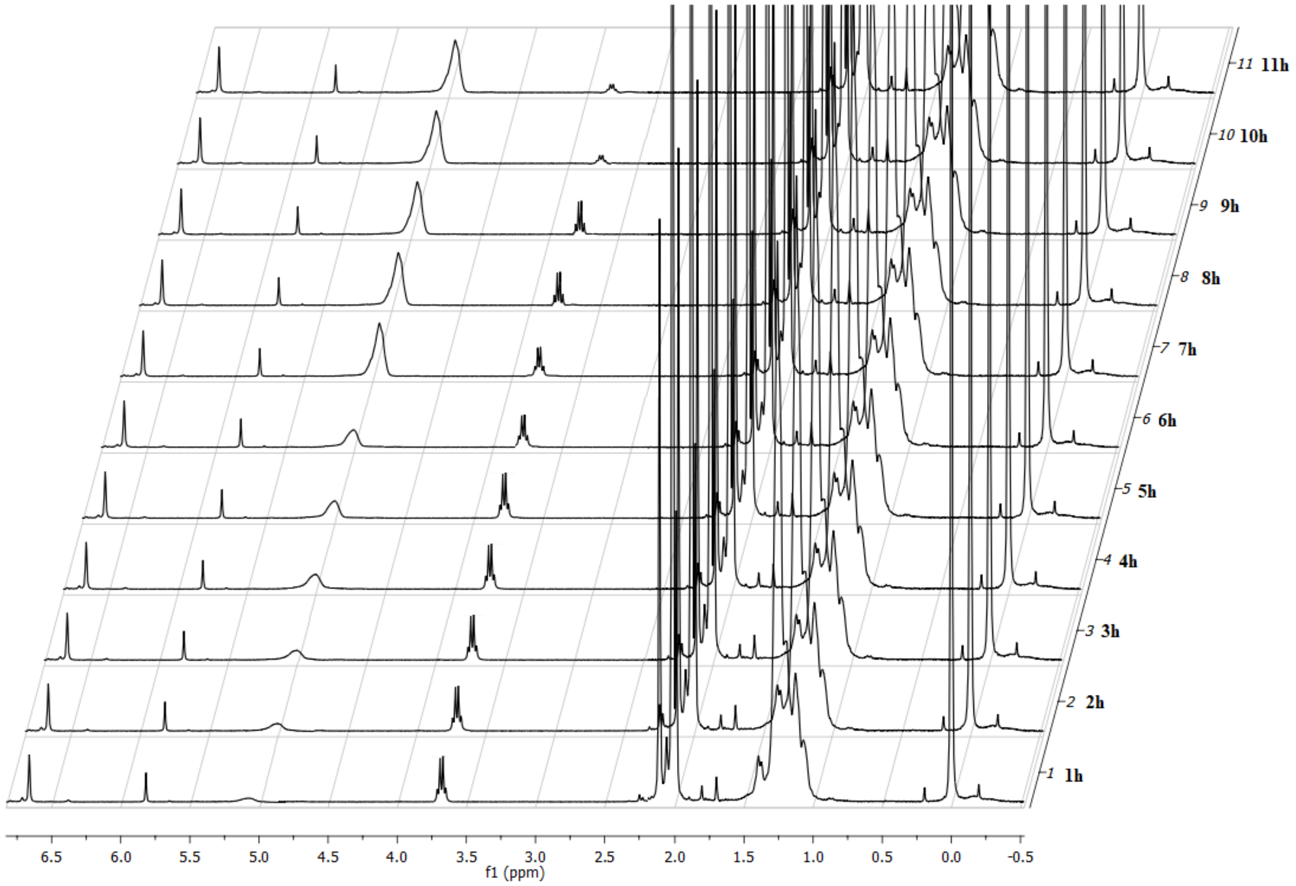

Figure S66. Stacked ^{1}H NMR spectra for the polymerization of *rac*-LA with [LA] = 0.20 M in toluene-d_8 (1 mL) at 60 °C catalyzed by [{(IMes)PH}$AlMe_3$](**4b**) (0.01 M).

(S7) PLAs: Stereochemistry and Microstructure

NMR spectroscopy provides a simple means to evaluate PLA stereochemistry and microstructure.[9] Through homonuclear decoupling ^{1}H NMR experiments, proton resonances of distinct methine carbons can be resolved. This results in a spectrum displaying distinct peaks corresponding to sequences of four consecutive methine carbons, known as a tetrad.[10] Eight arrangements exist for a sequence of four carbon atoms. However, PLA from *rac*-lactide only displays five of these resonances in the absence of epimerization due to the dimeric nature of the monomer. Two stereocenters of the same configuration are set with each insertion; therefore, resonances corresponding to the *rrr*, *rrm*, and *mrr* tetrads are not observed by ^{1}H NMR spectroscopy. Statistical analysis of the tetrad distribution in the decoupled ^{1}H NMR spectrum is used to differentiate two different stereocontrol processes. In a site-control process, the selectivity is defined in terms of *meso* (P_m), defined as the probability that a given enantiomer of the catalyst will react preferentially with one enantiomer of *rac*-LA. Chain-end control is defined in terms of probability of a *meso* (P_m) or *racemic* (P_r) placement (Table S12).

Table S12. Tetrad probabilities based on Bernoullian and enantiomorphic site control statistics for PLA from *rac*-LA.

Tetrad	chain-end control Bernouillan	site-control non-Bernouillan
[*mmm*]	$P_m{}^2 + P_rP_m/2$	$[P_m{}^2 + (1-P_m)^2 + P_m{}^3 + (1-P_m)^3]/2$
[*mmr*]	$P_rP_m/2$	$[P_m{}^2(1-P_m) + P_m(1-P_m)^2]/2$
[*rmm*]	$P_rP_m/2$	$[P_m{}^2(1-P_m) + P_m(1-P_m)^2]/2$
[*rmr*]	$P_r{}^2/2$	$[P_m{}^2(1-P_m) + P_m(1-P_m)^2]/2$
[*mrm*]	$(P_r{}^2 + P_rP_m)/2$	$[P_m(1-P_m) + P_m(1-P_m)]/2$
[*rrm*]	0	0
[*mrr*]	0	0
[*rrr*]	0	0

P_m and P_r are the probabilities of *meso* and *racemic* placement.

Table S13. *n*-ades probabilities based on Bernoullian statistics for PLA in terms of P_m.

n-ades	Probability				
Dyads	[*m*]	[*r*]			
	$(P_m+1)/2$	$(1-P_m)/2$			
Triads	[*mm*]	[*mr*]	[*rm*]		
	P_m	$(1-P_m)/2$	$(1-P_m)/2$		
Tetrads	[*mmm*]	[*mrm*]	[*mmr*]	[*rmm*]	[*rmr*]
	$P_m(P_m+1)/2$	$(1-P_m)/2$	$P_m(1-P_m)/2$	$P_m(1-P_m)/2$	$(1-P_m)2/2$

In addition to information about tacticity, NMR spectroscopy can also provide a great deal of insight into the polymerization mechanism. Two types of mechanisms of stereocontrol can operate in lactide polymerizations. An enantiomorphic site control mechanism is one in which the asymmetric environment of the catalyst reacts with the monomer selectively. In the case of enantiomorphic site control, an initial preference for ROP of one enantiomer of lactide, corresponding to the chirality of the catalyst, would be expected. However, as the chiral purity of the monomer pool is changed towards the enantiomer of lactide opposite to that of the catalyst, the polymer would be expected to slowly reach high conversions resulting in a tapered stereoblock copolymer. By

contrast, a chain-end control mechanism is one in which the stereocenter of the growing polymer chain dictates the stereochemical outcome of the next monomer insertion. These two mechanisms produce polymers that can be distinguished by NMR spectroscopy due to distinct stereochemical errors. The tetrad distribution is therefore different in PLAs formed from *rac*-LA under these CEC or ESC mechanisms. Assuming PLA would form by only a CEC mechanism exclusively, relative tetrad intensities would be expected as follows: [*mrm*] = [*rmm*] = [*mmr*] ≠ [*rmr*] (*rmr* = 0).[11,12] In this case, stereoblocks could be generated when a growing PLA chain incidentally incorporated the LA enantiomer of opposite configuration to that of the last inserted enantiomer. This would create a stereoerror from which "normal" growth would form a new stereoblock of opposite configuration to the previous one. Conversely, the ESC mechanism should generate single insertion stereoerrors of the type −RRRRSSRRR−/− SSSSRRSSSS−. In the latter case, the tetrad ratio should be [*rmr*] = [*mmr*] = [*rmm*] = 2/[*mrm*].[13] Thus, the *rmr* signal is a clear indicator of these two mechanisms. Analysis of the NMR spectra of the PLAs produced here revealed that the tetrad ratios were not fully consistent with neither the ESC nor the CEC mechanism, thus strongly suggesting that both mechanisms concomitantly occurred. Thereby, it should be emphasized that in addition to conventional chain-end control, in which the insertion of either LLA or DLA is controlled by the chirality of the previously enchained monomer, the ligand might cooperatively induce enantiomorphic site control, effecting the stereoselectivity of the catalyst system.

The tetrad ratios of the resultant PLA are somewhat more close with the CEC mechanism than ESC mechanism here (S68, S71 and S73), so Bernoullian statistics can be used to define relationships between tetrad probabilities and P_m. In Table S13, the dyad, triads, tetrad intensities for the polymer sample were assigned. The P_m values were calculated from the formulas in Table S13 which are based on tetrad probabilities in the polymerization of *rac*-lactide as calculated from Bernoullian statistics.[10] The methine region of the (homonuclear coupled and decoupled) ^{1}H NMR spectra of a PLA prepared by polymerization of *rac*-LA with complex **4a** (Table 1, Entry 1–5) are shown in Figures S67-68, S70-71, S72-73, S74 and S75. This isotactic PLA displays only one major peak, corresponding to the *mmm* tetrad. Here we found that the relative ratio of the *rmr* tetrad increases in respect to both the *mmm* and the *rmm*/*mmr* tetrads as a function of monomer equiv. Namely, the CEC control becomes more dominant in lower monomer equiv.(100-200 equiv. and Entries 1–2, Table 2). Whereas with increasing the monomer equiv. the contribution of the ESC also simultenously participate with CEC . Analysis of the NMR spectra of the PLAs produced here (Entries 5, Table 2, Figure 75) revealed the tetrad ratios to be [*rmr*] = 0.06 [*mmr*] = 0.09 [*rmm*] = 0.05 and [*mrm*] = 0.04, which were consistent with neither the ESC nor the CEC mechanism, thus strongly suggesting that both mechanisms concomitantly occurred which can bias the stereocontrol, creating additional stereoerrors. As a result, this both mechanism different interplay of opposing effects which demises the P_m value. Same observation already reported in literature. [14] Also we measured the P_m value as a function of time in the ROP of 500 equiv of LA with **4a** (included ^{13}C NMR data as Figure S83) which showed that the P_m value remain constant at higher conversion. This confirmed the stability of the catalyst under the polymerization condition.

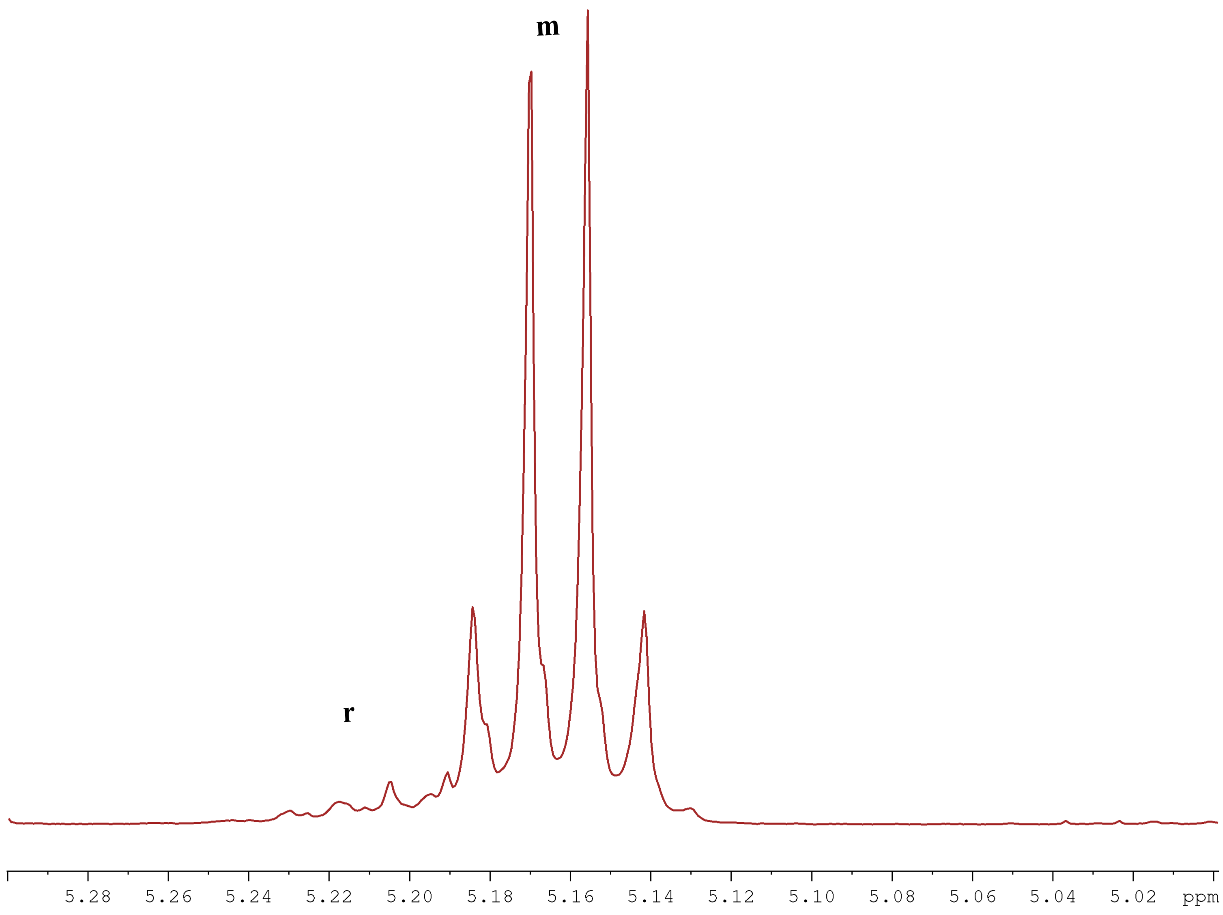

Figure S67. Methine region of the ^{1}H NMR spectrum (500 MHz, $CDCl_3$) of a PLA prepared by polymerization of *rac*-LA with complex **4a** (Table 2, Entry 1). Observation of dyads [*m*] and [*r*].

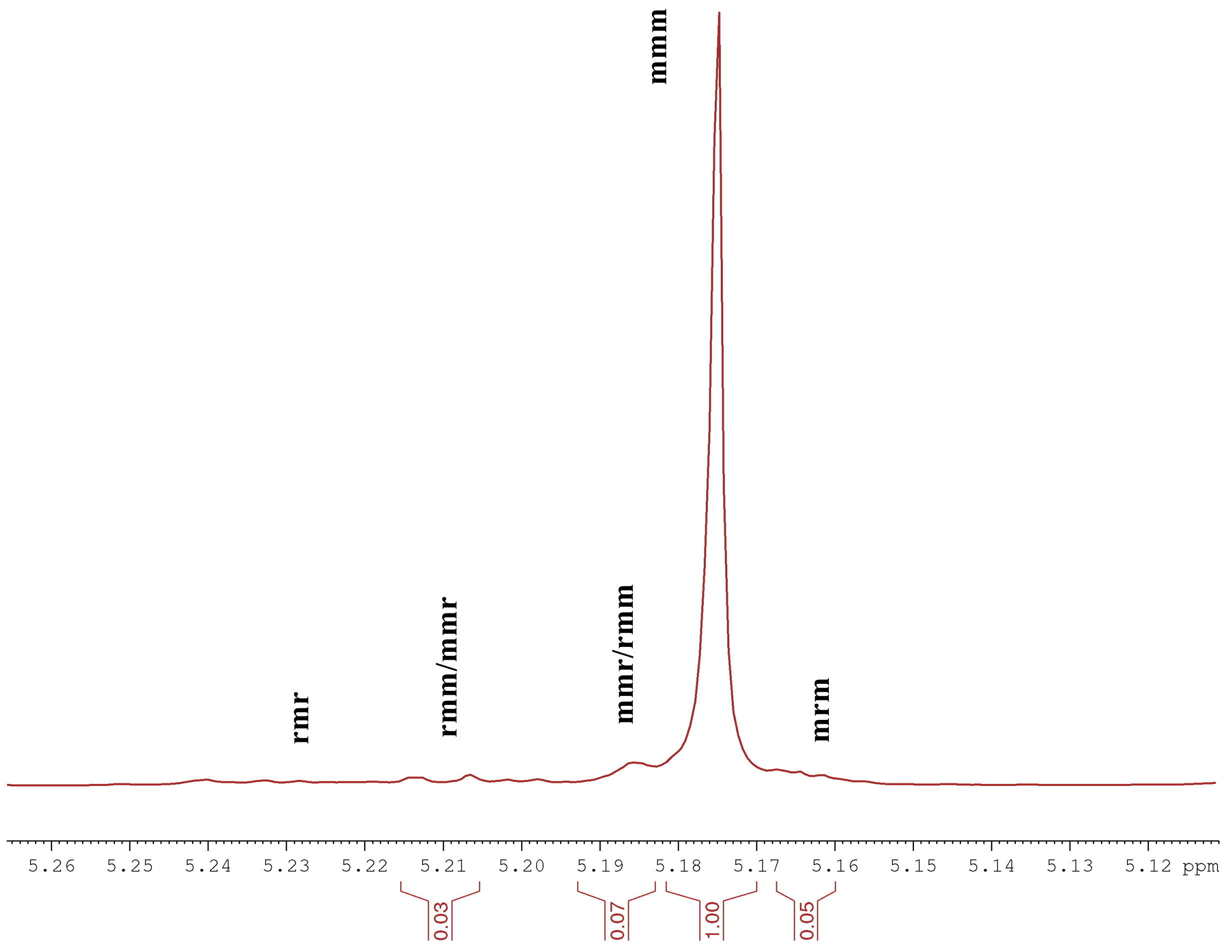

Figure S68. Methine region of the homodecoupled ^{1}H NMR spectrum (500 MHz, $CDCl_3$) of a PLA prepared by polymerization of *rac*-LA with complex **4a** (Table 2, Entry 1). Observation of tetrads.

Analysis

[mmm] = $P_m (P_m + 1)/2$
[mmr] = $P_m (1 - P_m)/2$
[rmm] = $P_m (1 - P_m)/2$
[rmr] = $(1 - P_m)^2/2$
[mrm] = $(1 - P_m)/2$

**Peak**	**Integration**	P_m
[mmm]	0.87	0.91
[mmr]	0.06	0.86
[rmm]	0.026	0.945
[rmr]	0.001	0.95
[mrm]	0.04	0.92
**Avarage**		**0.92**

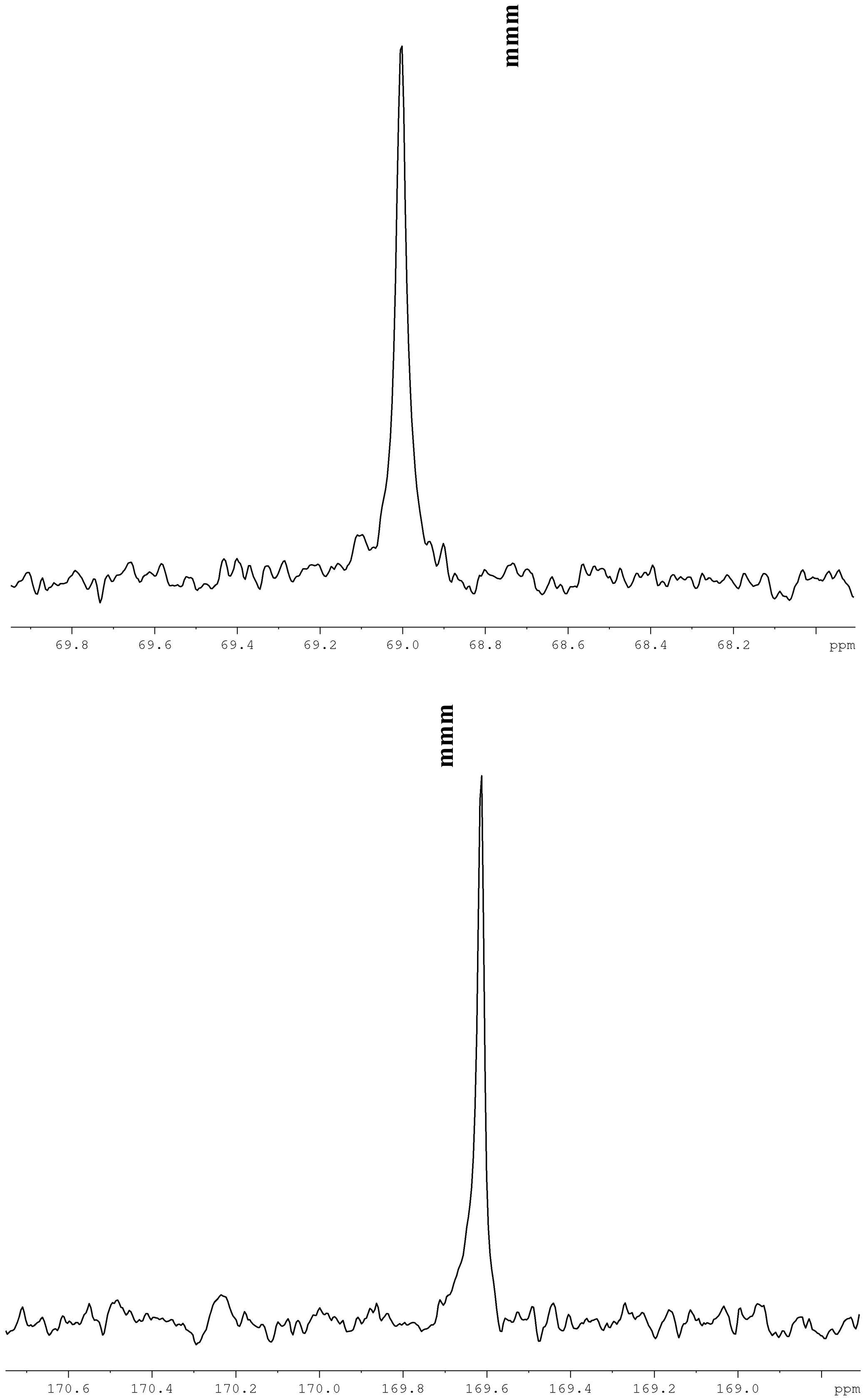

Figure S69. Methine (top) and carbonyl (bottom) region of the ^{13}C NMR spectrum (125.8 MHz, $CDCl_3$) of a PLA prepared by polymerization of *rac*-LA with complex **4a** (Table 2, Entry 1).

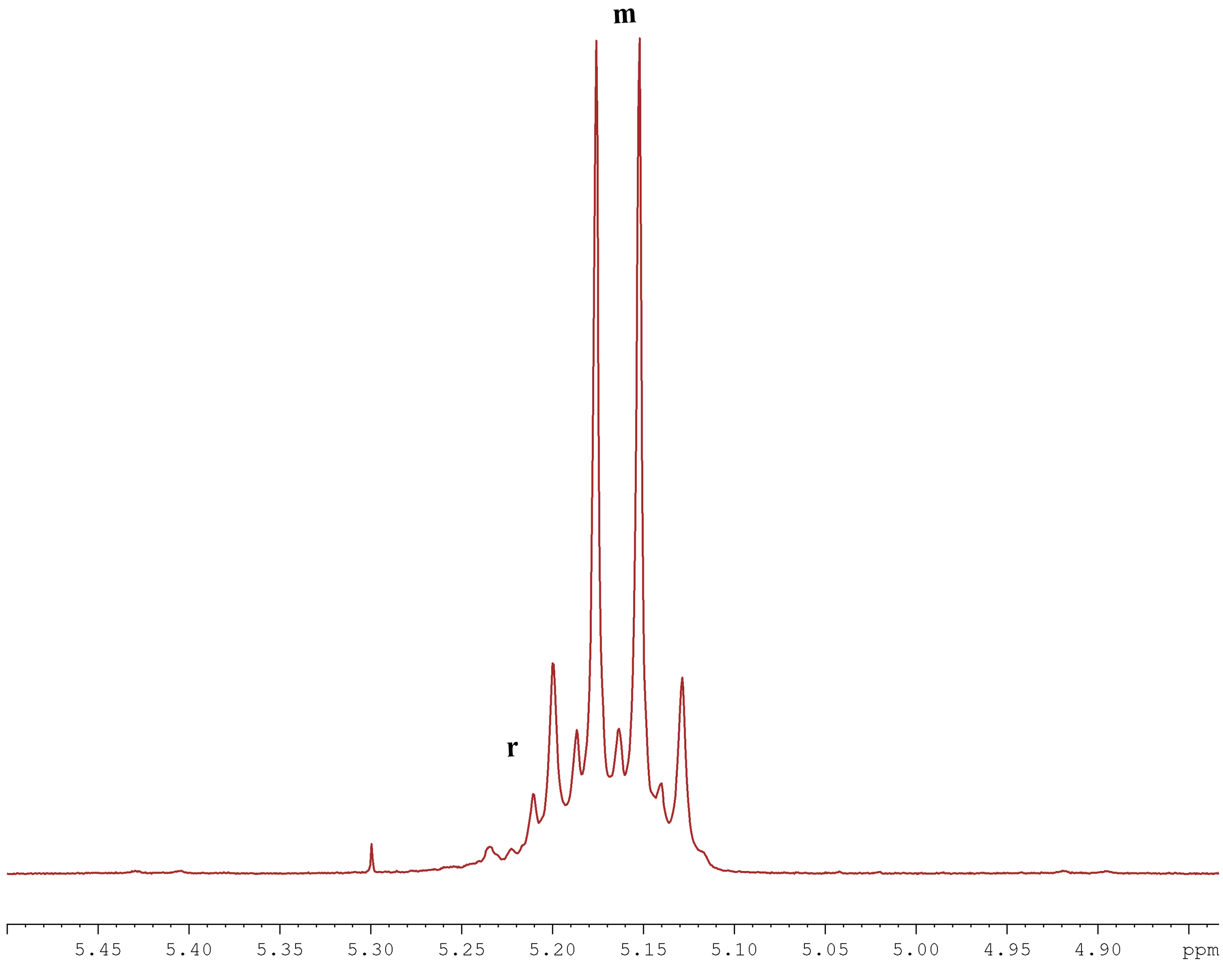

Figure S70. Methine region of the ^{1}H NMR spectrum (500 MHz, $CDCl_3$) of a PLA prepared by polymerization of *rac*-LA with complex **4a** (Table 2, Entry 2). Observation of dyads [*m*] and [*r*].

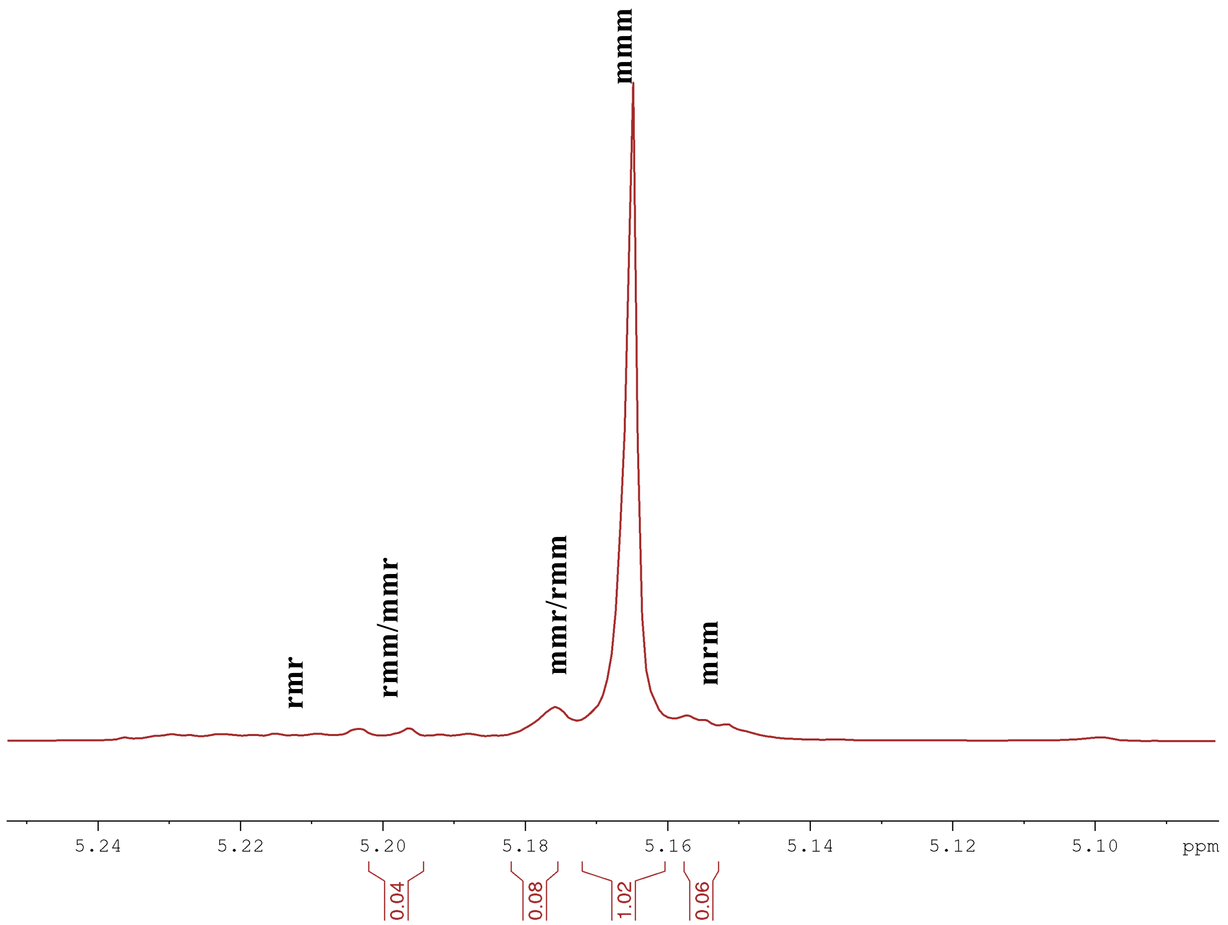

Figure S71. Methine region of the homodecoupled ^{1}H NMR spectrum (500 MHz, $CDCl_3$) of a PLA prepared by polymerization of *rac*-LA with complex **4a** (Table 2, Entry 2). Observation of tetrads.

Analysis

[mmm] = $P_m (P_m + 1)/2$
[mmr] = $P_m (1 - P_m)/2$
[rmm] = $P_m (1 - P_m)/2$
[rmr] = $(1 - P_m)^2/2$
[mrm] = $(1 - P_m)/2$

Peak	Integration	P_m
[mmm]	0.85	0.90
[mmr]	0.066	0.84
[rmm]	0.03	0.94
[rmr]	0.001	0.95
[mrm]	0.05	0.90
**Avarage**		**0.906**

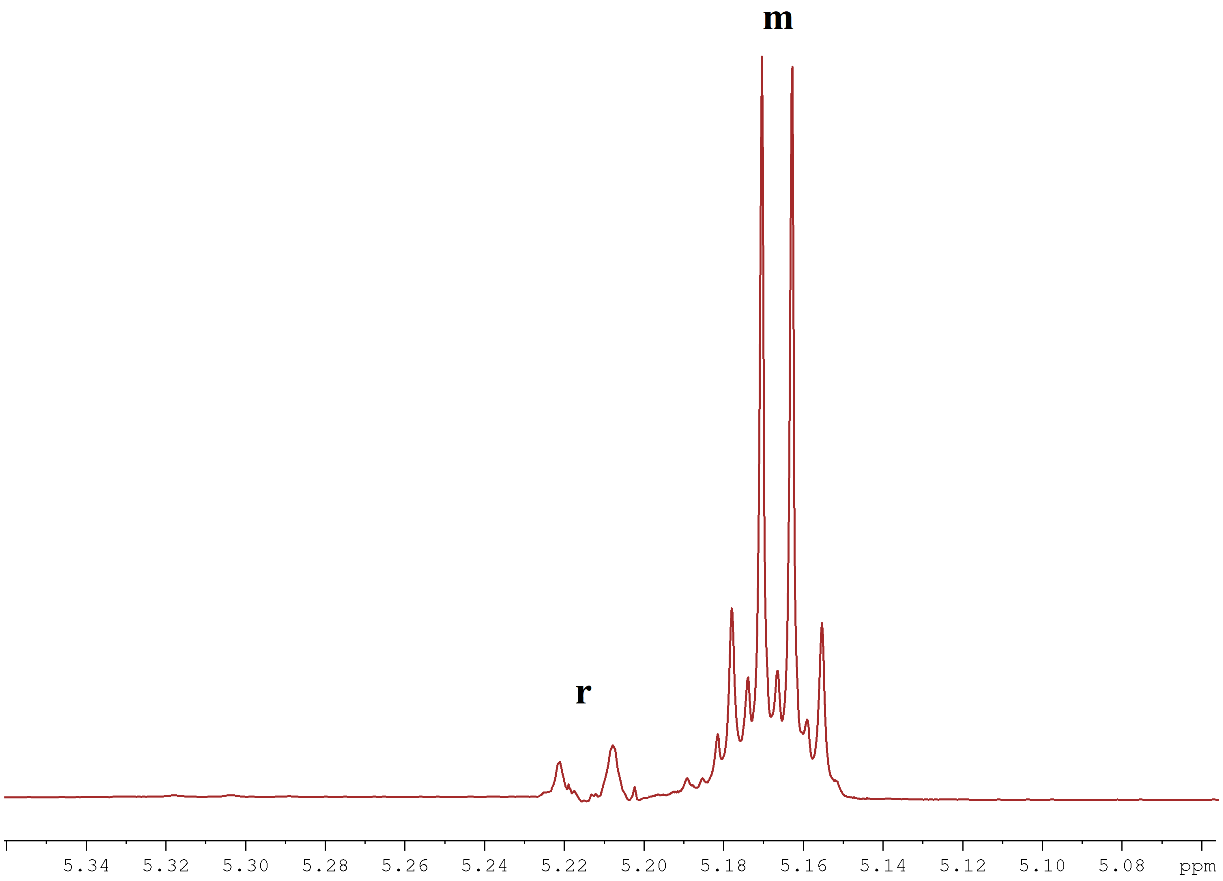

Figure S72. Methine region of the ^{1}H NMR spectrum (500 MHz, $CDCl_3$) of a PLA prepared by polymerization of *rac*-LA with complex **4a** (Table 2, Entry 3). Observation of dyads [*m*] and [*r*].

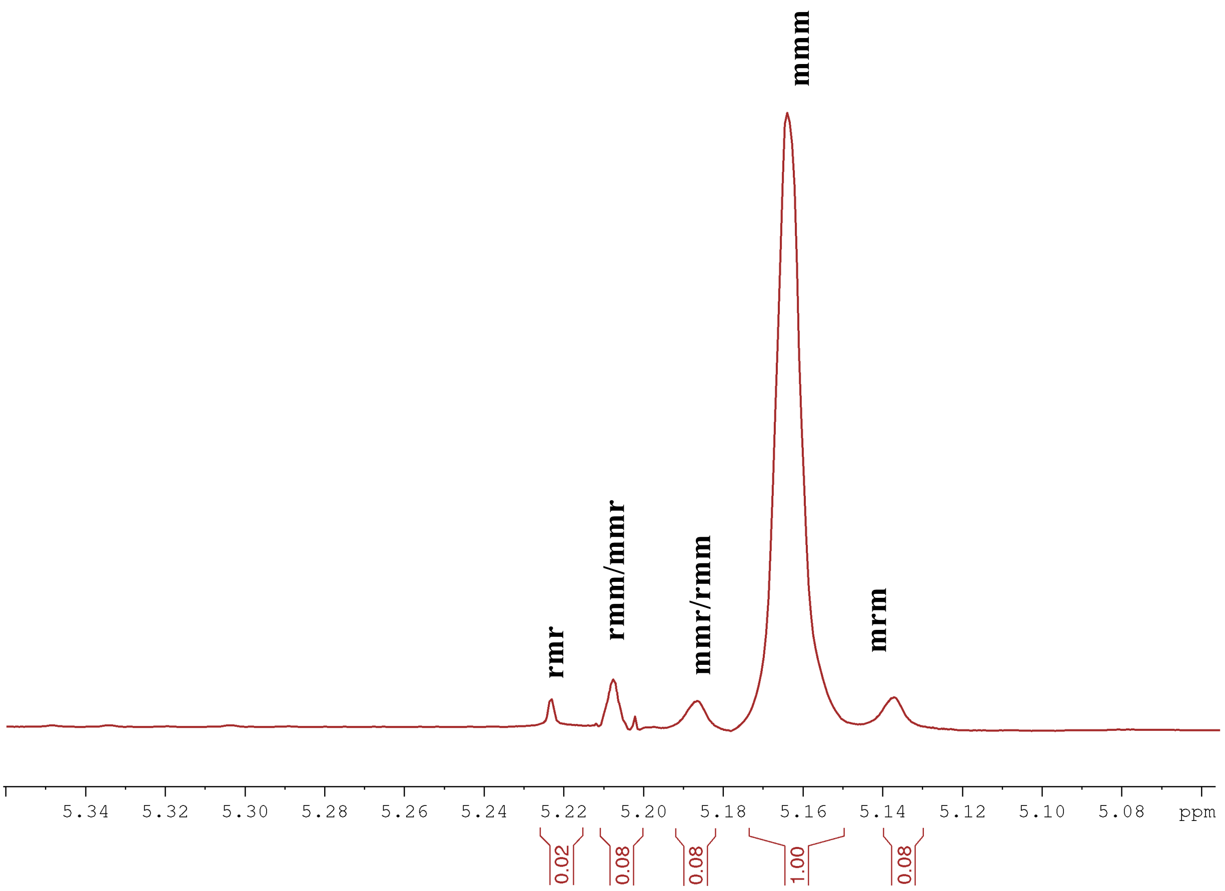

Figure S73. Methine region of the homodecoupled ^{1}H NMR spectrum (500 MHz, $CDCl_3$) of a PLA prepared by polymerization of *rac*-LA with complex **4a** (Table 2, Entry 3). Observation of tetrads.

Analysis

[mmm] = P_m (P_m +1)/2
[mmr] = P_m (1 - P_m)/2
[rmm] = P_m (1 - P_m)/2
[rmr] = (1 - P_m)^2/2
[mrm] = (1 - P_m)/2

Peak	Integration	P_m
[mmm]	0.79	0.85
[mmr]	0.063	0.85
[rmm]	0.063	0.85
[rmr]	0.015	0.82
[mrm]	0.063	0.87
**Avarage**		**0.85**

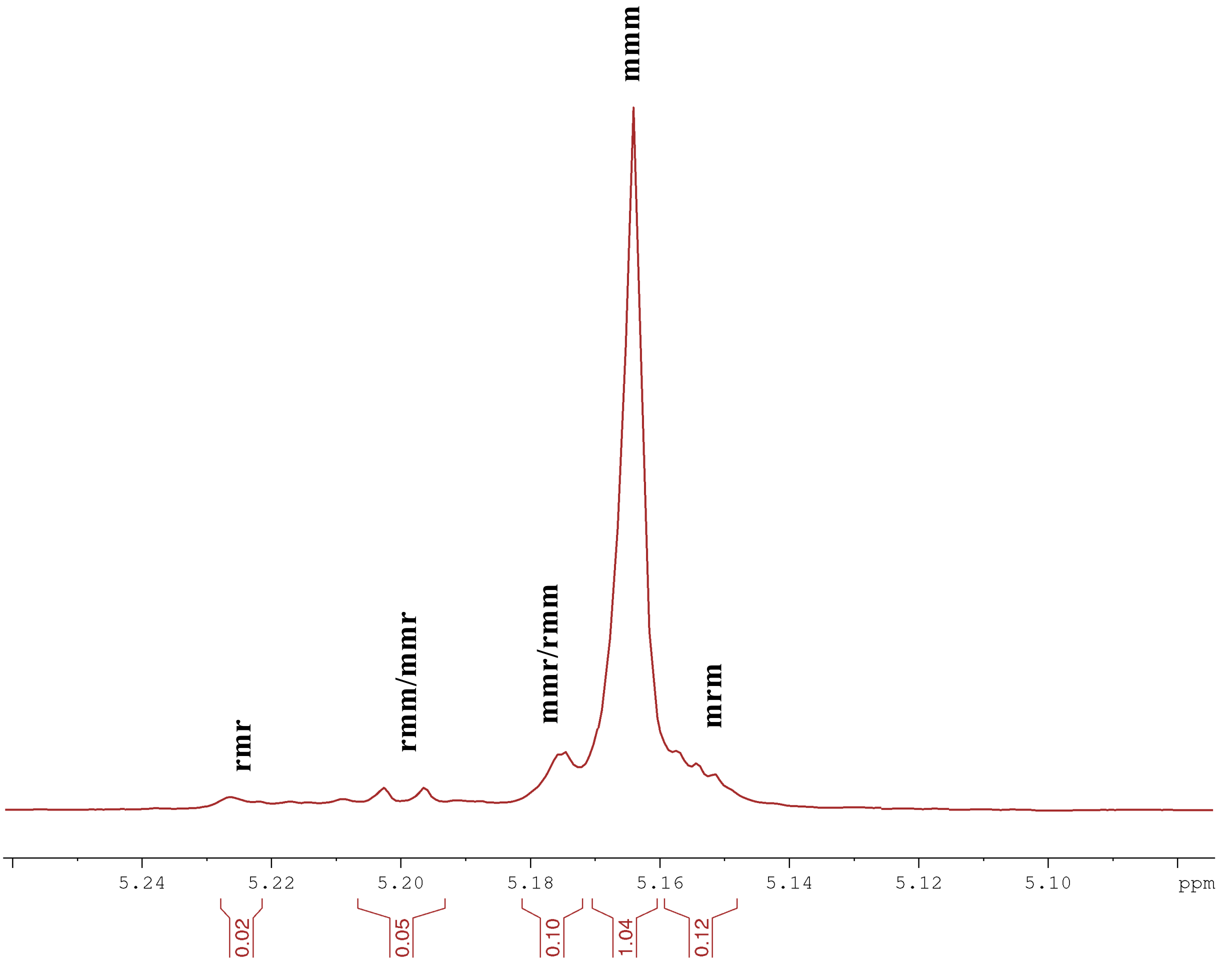

Figure S74. Methine region of the homodecoupled ^1H NMR spectrum (500 MHz, $CDCl_3$) of a PLA prepared by polymerization of *rac*-LA with complex **4a** (Table 2, Entry 4). Observation of tetrads.

Analysis

[mmm] = $P_m (P_m + 1)/2$

[mmr] = $P_m (1 - P_m)/2$

[rmm] = $P_m (1 - P_m)/2$

[rmr] = $(1 - P_m)^2/2$

[mrm] = $(1 - P_m)/2$

**Peak**	**Integration**	P_m
[mmm]	0.78	0.84
[mmr]	0.08	0.80
[rmm]	0.04	0.91
[rmr]	0.015	0.81
[mrm]	0.09	0.82
**Avarage**		**0.83**

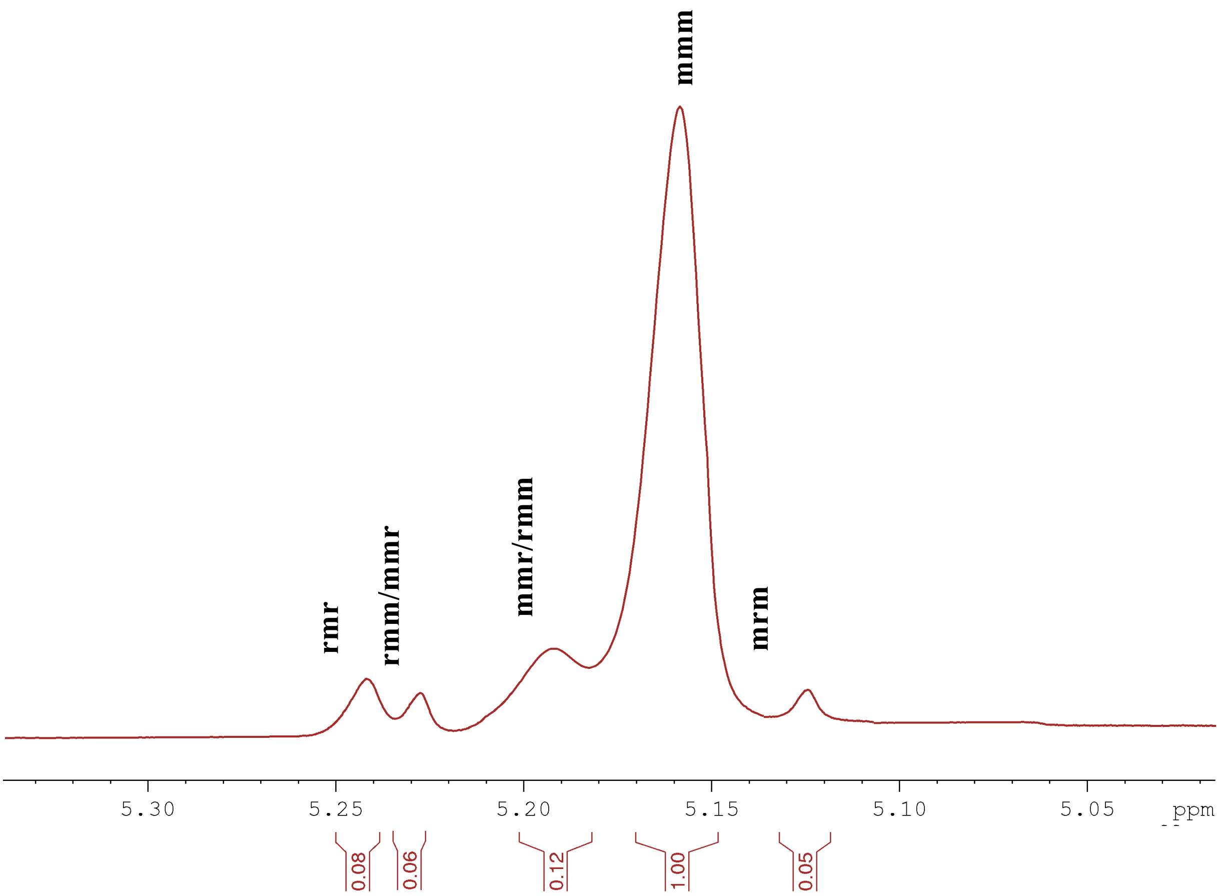

Figure S75. Methine region of the homodecoupled ^{1}H NMR spectrum (500 MHz, $CDCl_3$) of a PLA prepared by polymerization of *rac*-LA with complex **4a** (Table 2, Entry 5). Observation of tetrads.

Analysis

[mmm] = P_m (P_m +1)/2
[mmr] = P_m (1 - P_m)/2
[rmm] = P_m (1 - P_m)/2
[rmr] = (1 - P_m)^2/2
[mrm] = (1 - P_m)/2

Peak	Integration	P_m
[mmm]	0.76	0.83
[mmr]	0.09	0.76
[rmm]	0.048	0.89
[rmr]	0.06	0.65
[mrm]	0.04	0.92
**Avarage**		**0.81**

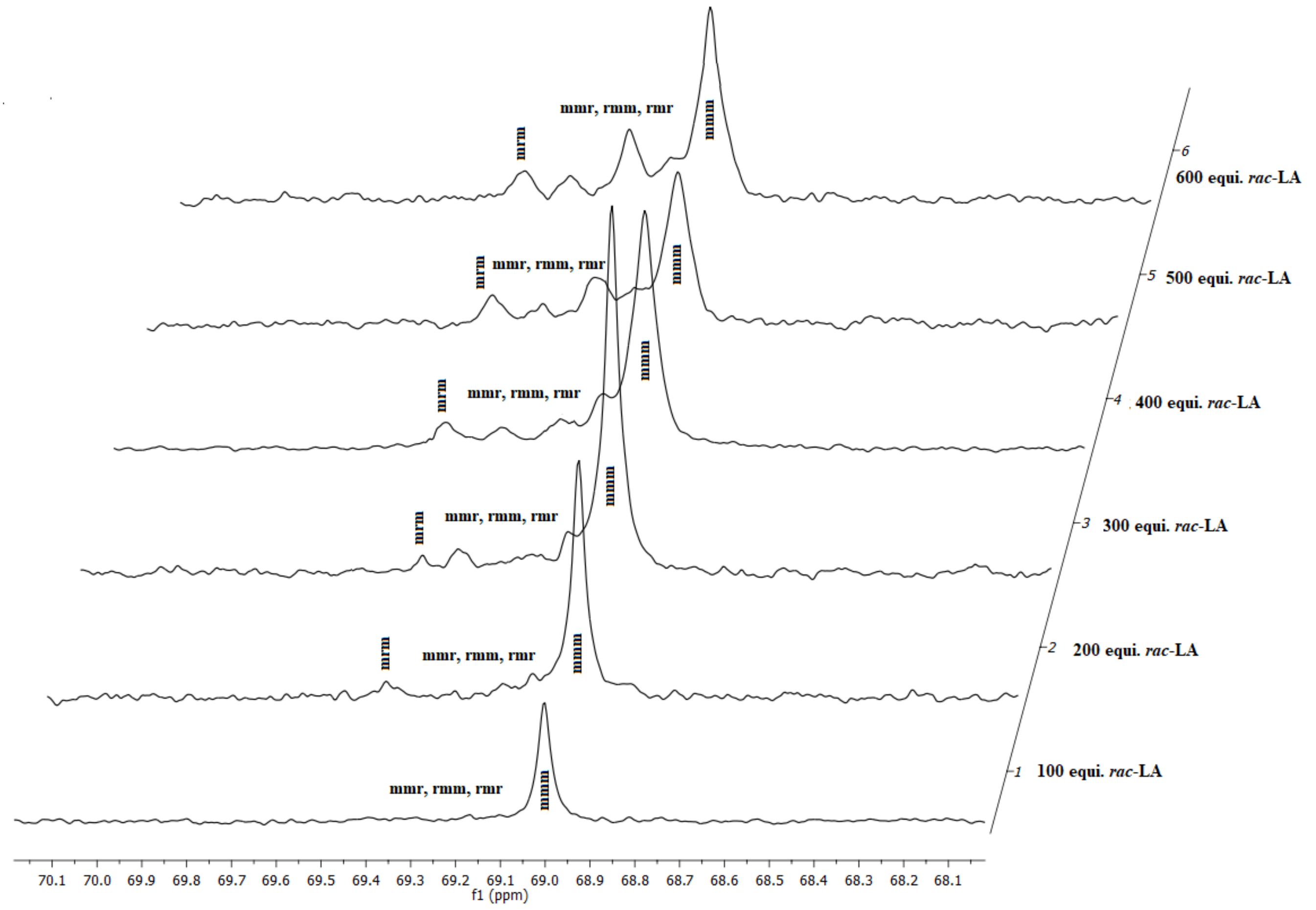

Figure S76. Stack ^{13}C NMR spectrum (125.8 MHz, $CDCl_3$) of the methine region of the PLA prepared by polymerization of *rac*-LA with complex **4a** at different monomer equiv. (Table 2, Entry 1-5).

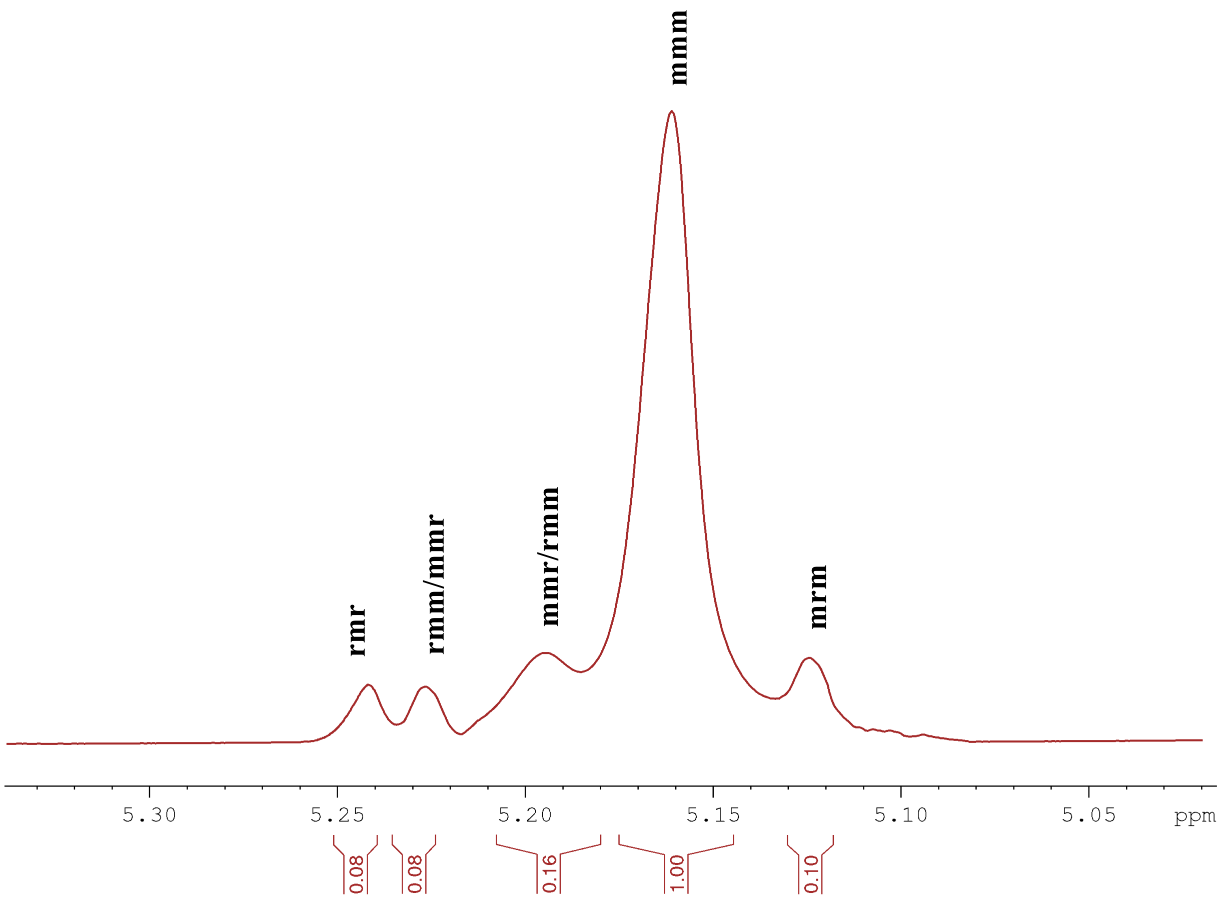

Figure S77. Methine region of the homodecoupled ^{1}H NMR spectrum (500 MHz, $CDCl_3$) of a PLA prepared by polymerization of *rac*-LA with complex **4b** (Table 2, Entry 6). Observation of tetrads.

Analysis

[mmm] = P_m (P_m +1)/2
[mmr] = P_m (1 - P_m)/2
[rmm] = P_m (1 - P_m)/2
[rmr] = (1 - P_m)^2/2
[mrm] = (1 - P_m)/2

**Peak**	**Integration**	$\boldsymbol{P_m}$
[mmm]	0.704	0.79
[mmr]	0.11	0.62
[rmm]	0.06	0.85
[rmr]	0.056	0.66
[mrm]	0.07	0.86
**Avarage**		**0.75**

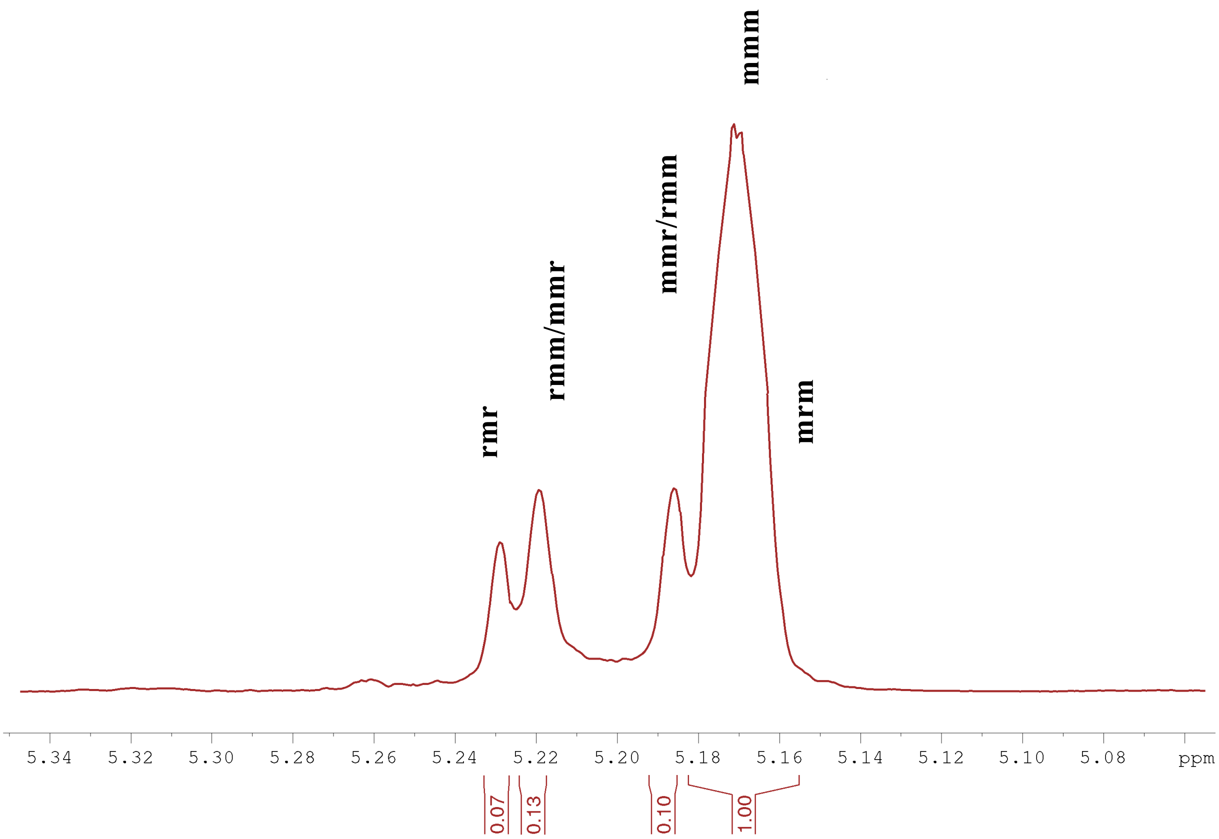

Figure S78. Methine region of the homodecoupled ^{1}H NMR spectrum (500 MHz, $CDCl_3$) of a PLA prepared by polymerization of *rac*-LA with complex **4b** (Table 2, Entry 7). Observation of tetrads.

Analysis

[mmm] = P_m (P_m +1)/2
[mmr] = P_m (1 - P_m)/2
[rmm] = P_m (1 - P_m)/2
[rmr] = $(1 - P_m)^2/2$
[mrm] = (1 - P_m)/2

**Peak**	**Integration**	P_m
[mmm]	0.77	0.84
[mmr]	0.08	0.79
[rmm]	0.10	0.71
[rmr]	0.054	0.67
[mrm]	0	-
**Avarage**		**0.75**

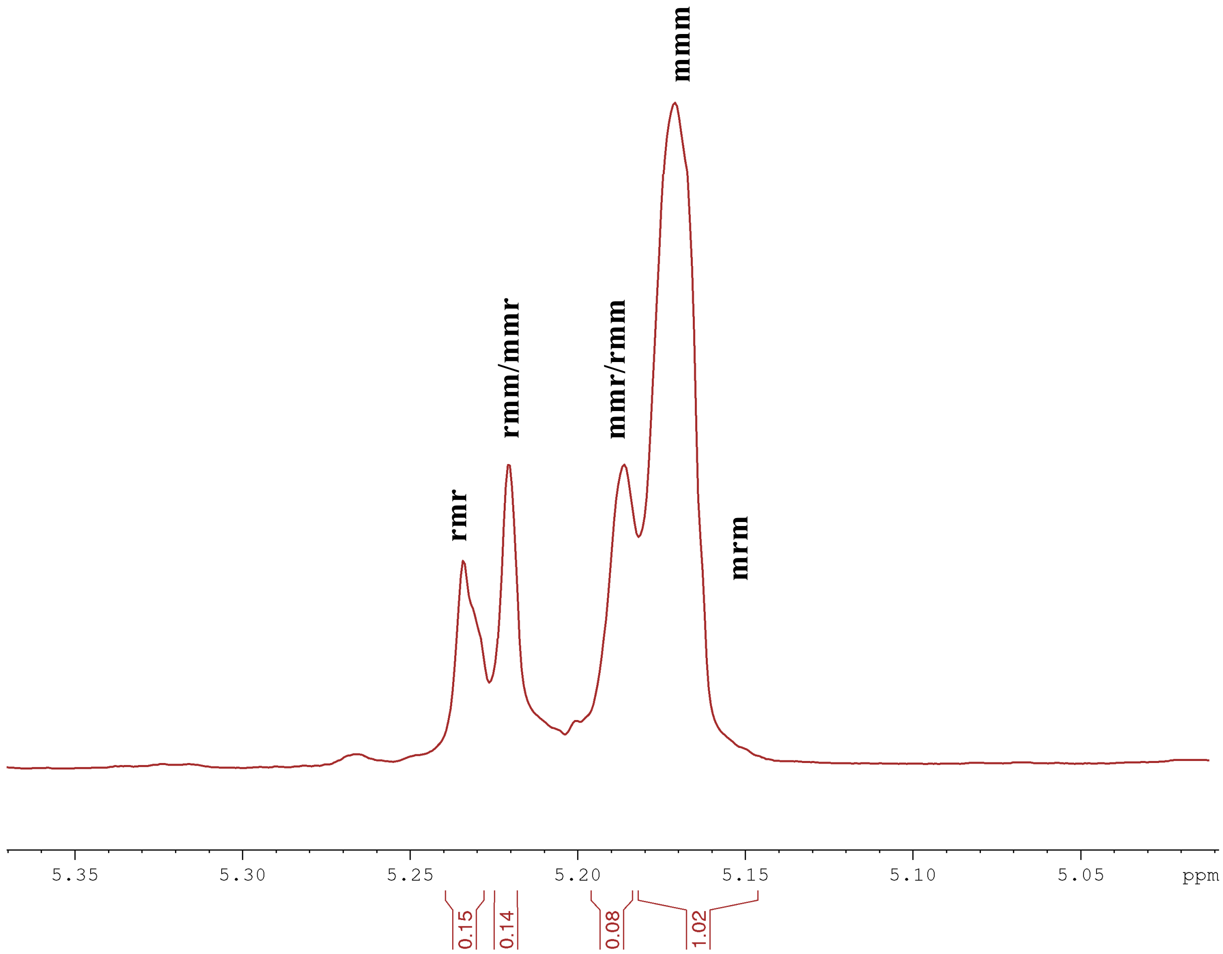

Figure S79. Methine region of the homodecoupled ^{1}H NMR spectrum (500 MHz, $CDCl_3$) of a PLA prepared by polymerization of *rac*-LA with complex **4b** (Table 2, Entry 8). Observation of tetrads.

Analysis

[mmm] = P_m (P_m +1)/2
[mmr] = P_m (1 - P_m)/2
[rmm] = P_m (1 - P_m)/2
[rmr] = (1 - P_m)^2/2
[mrm] = (1 - P_m)/2

**Peak**	**Integration**	P_m
[mmm]	0.73	0.81
[mmr]	0.06	0.85
[rmm]	0.10	0.71
[rmr]	0.1	0.55
[mrm]	0.0	-
**Avarage**		**0.73**

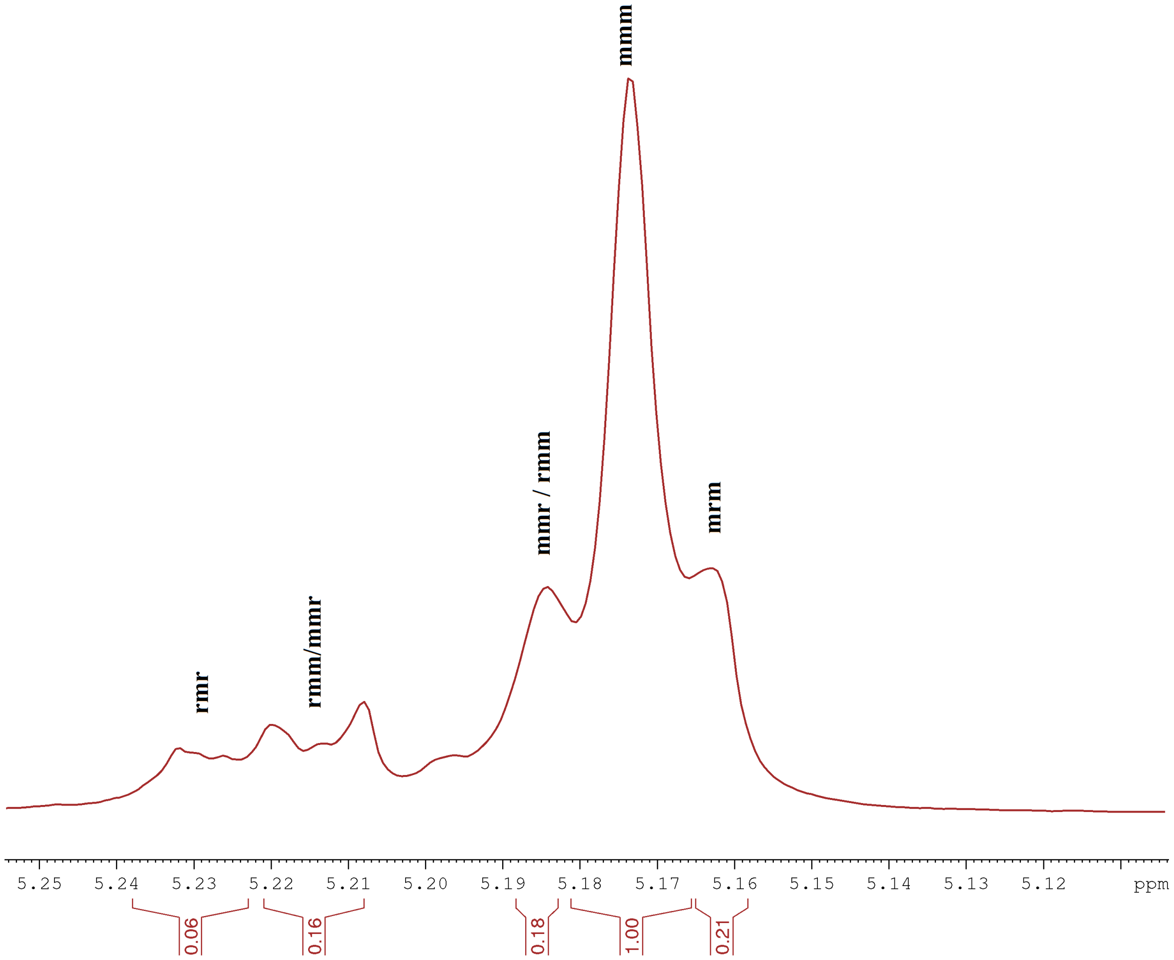

Figure S80. Methine region of the homodecoupled ^{1}H NMR spectrum (500 MHz, $CDCl_3$) of a PLA prepared by polymerization of *rac*-LA with complex **4b** (Table 2, Entry 9). Observation of tetrads.

Analysis

[mmm] = $P_m (P_m + 1)/2$
[mmr] = $P_m (1 - P_m)/2$
[rmm] = $P_m (1 - P_m)/2$
[rmr] = $(1 - P_m)^2/2$
[mrm] = $(1 - P_m)/2$

**Peak**	**Integration**	$\boldsymbol{P_m}$
[mmm]	0.62	0.72
[mmr]	0.11	0.62
[rmm]	0.10	0.71
[rmr]	0.035	0.74
[mrm]	0.13	0.74
**Avarage**		**0.71**

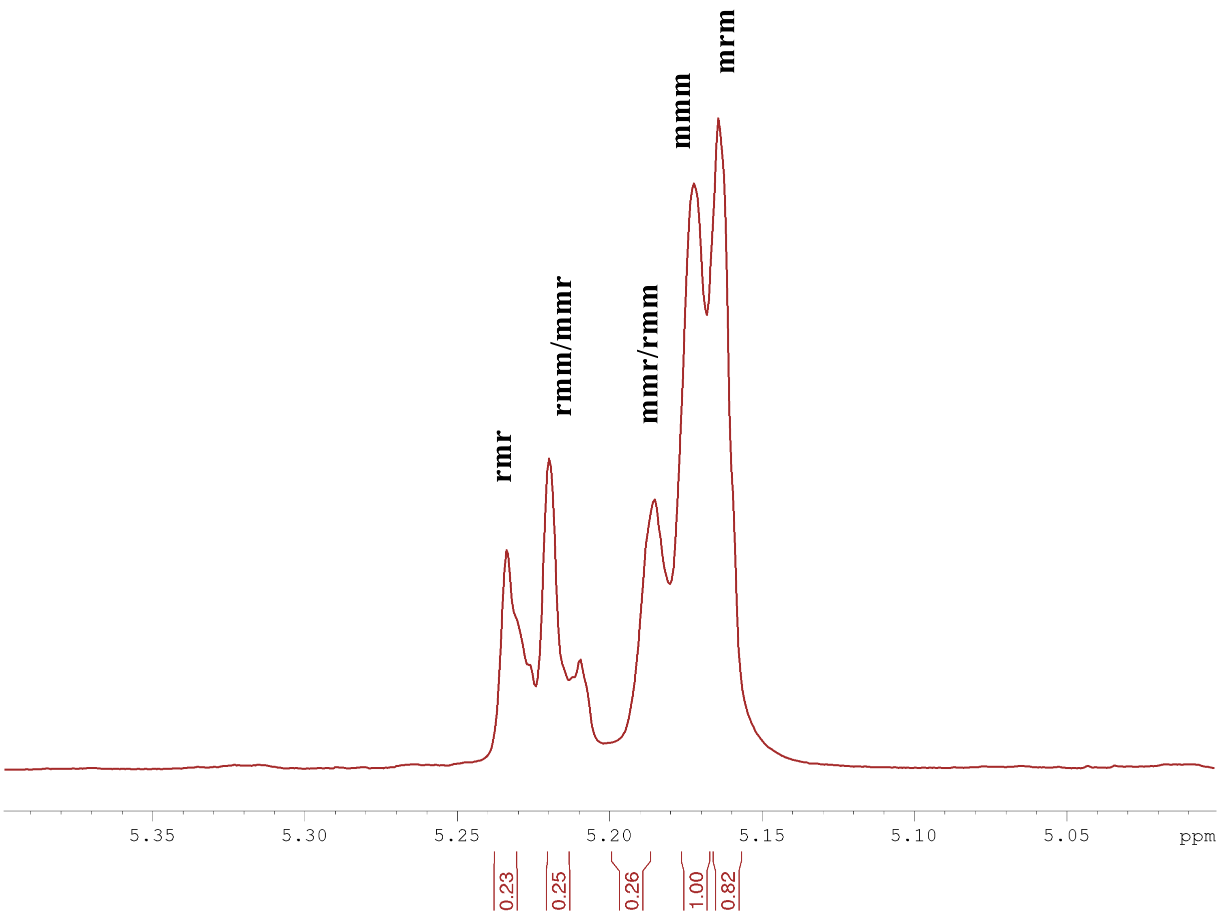

Figure S81. Methine region of the homodecoupled ^{1}H NMR spectrum (500 MHz, $CDCl_3$) of a PLA prepared by polymerization of *rac*-LA with complex **4c** (Table 2, Entry 11). Observation of tetrads.

Analysis

[mmm] = $P_m (P_m +1)/2$
[mmr] = $P_m (1 - P_m)/2$
[rmm] = $P_m (1 - P_m)/2$
[rmr] = $(1 - P_m)^2/2$
[mrm] = $(1 - P_m)/2$

Peak	Integration	P_m
[mmm]	0.39	0.52
[mmr]	0.10	0.72
[rmm]	0.098	0.73
[rmr]	0.09	0.57
[mrm]	0.32	0.36
**Avarage**		**0.58**

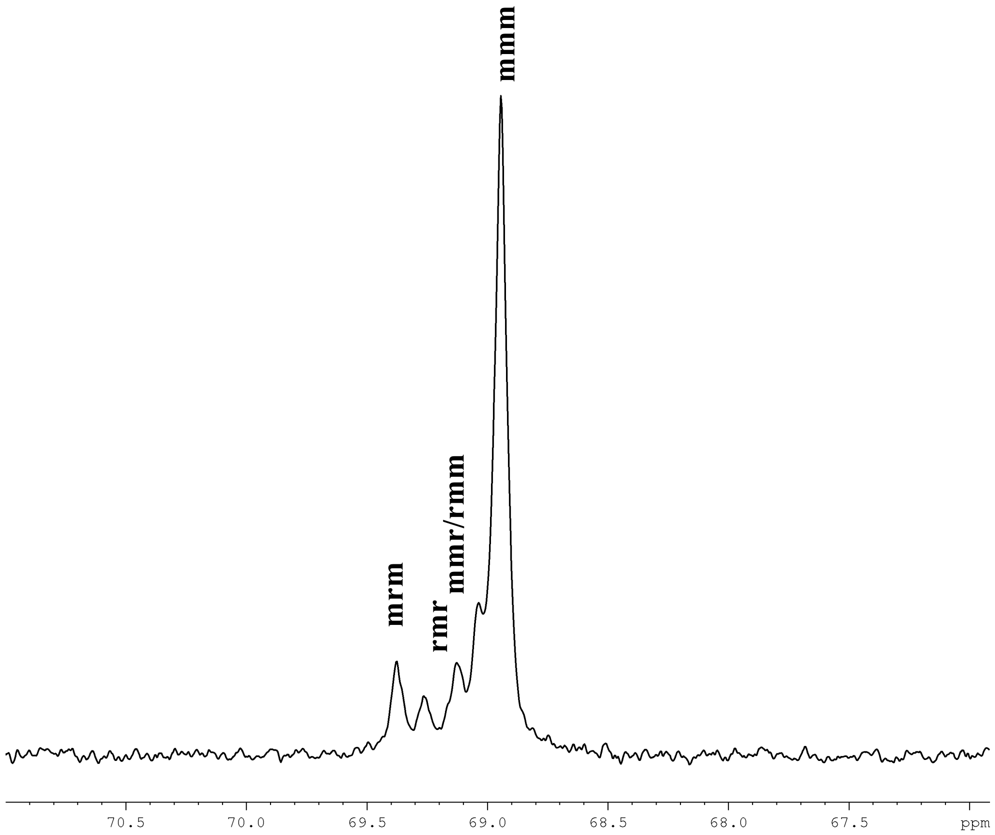
mmm
mmr/rmm
mrm
rmr
70.5
70.0
69.5
69.0
68.5
68.0
67.5
ppm

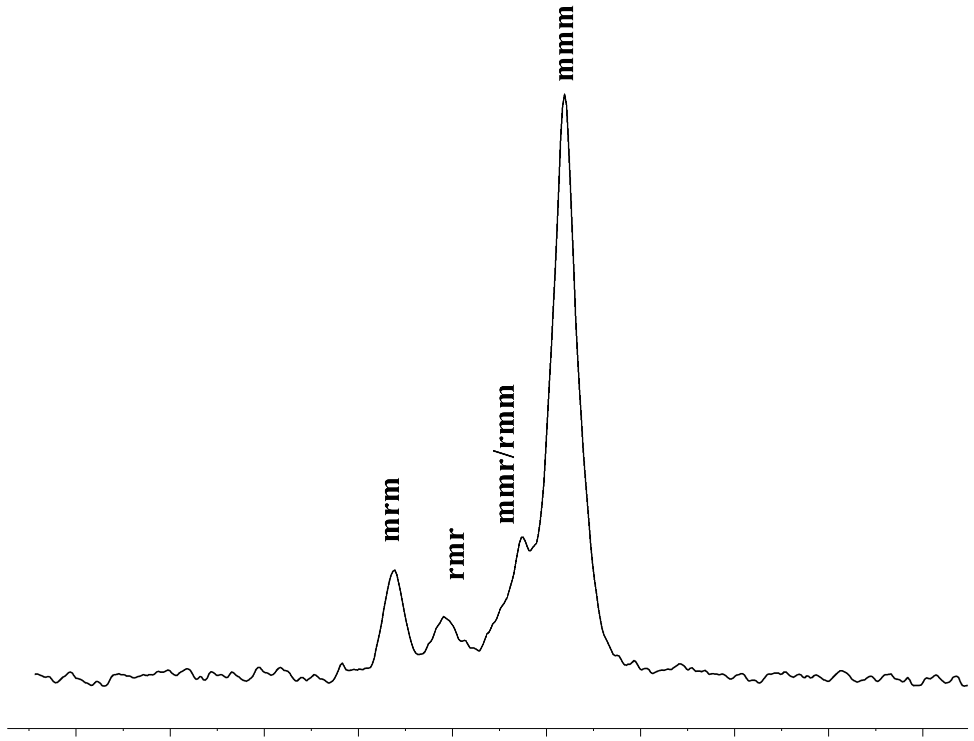
mmm
mmr/rmm
mrm
rmr
70.0
69.8
69.6
69.4
69.2
69.0
68.8
68.6
68.4
ppm

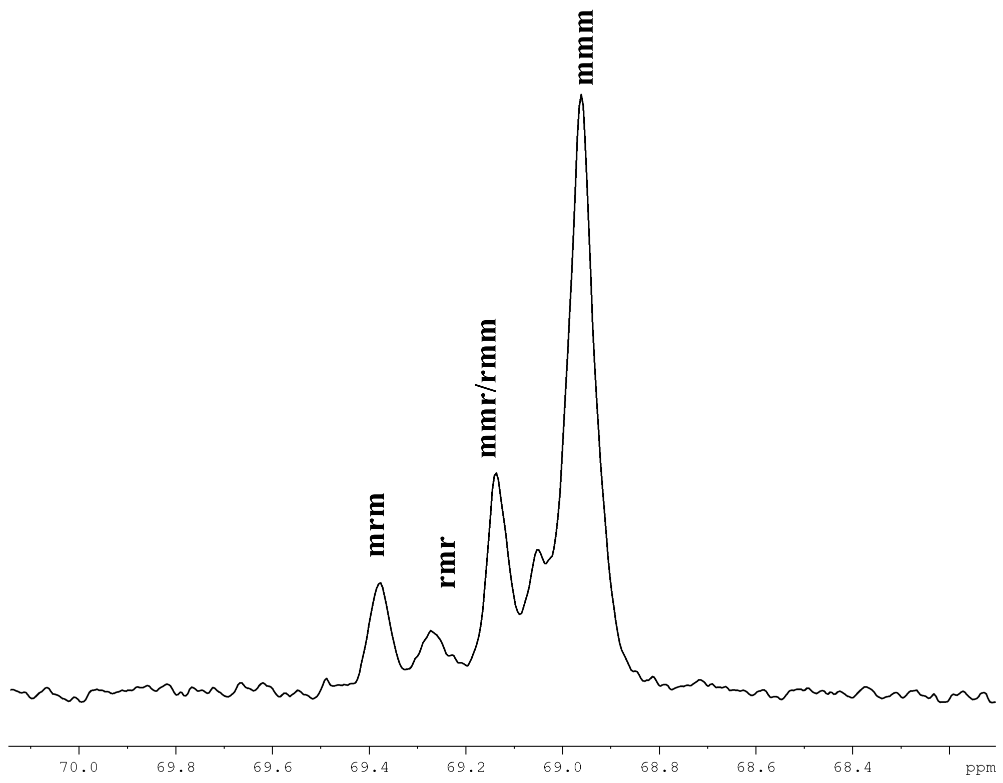

Figure S82. Methine region of ^{13}C NMR spectra (125.8 MHz, $CDCl_3$) of various PLAs prepared by polymerization of *rac*-LA with complex **4b** and **4c** from entries 6, 7 and 11 (from top to bottom, Table 2).

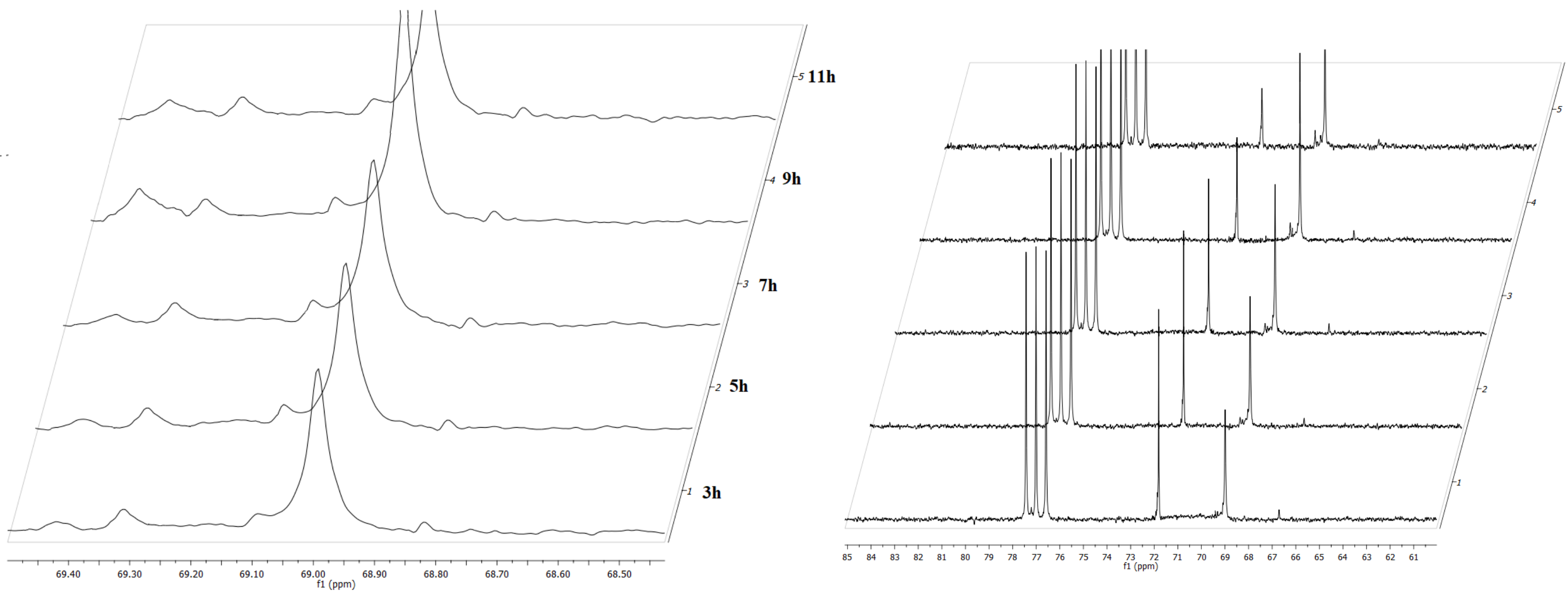

Figure S83. Stack ^{13}C NMR spectrum (125.8 MHz, $CDCl_3$) of the methine region of the PLA prepared by polymerization of *rac*-LA with complex **4a** at different conservion (Reaction condition = $[LA]_0/[M]$ = 500:1, 60 °C, toluene). Right: Same spectrum, expanded region *δ* 60–85 ppm.

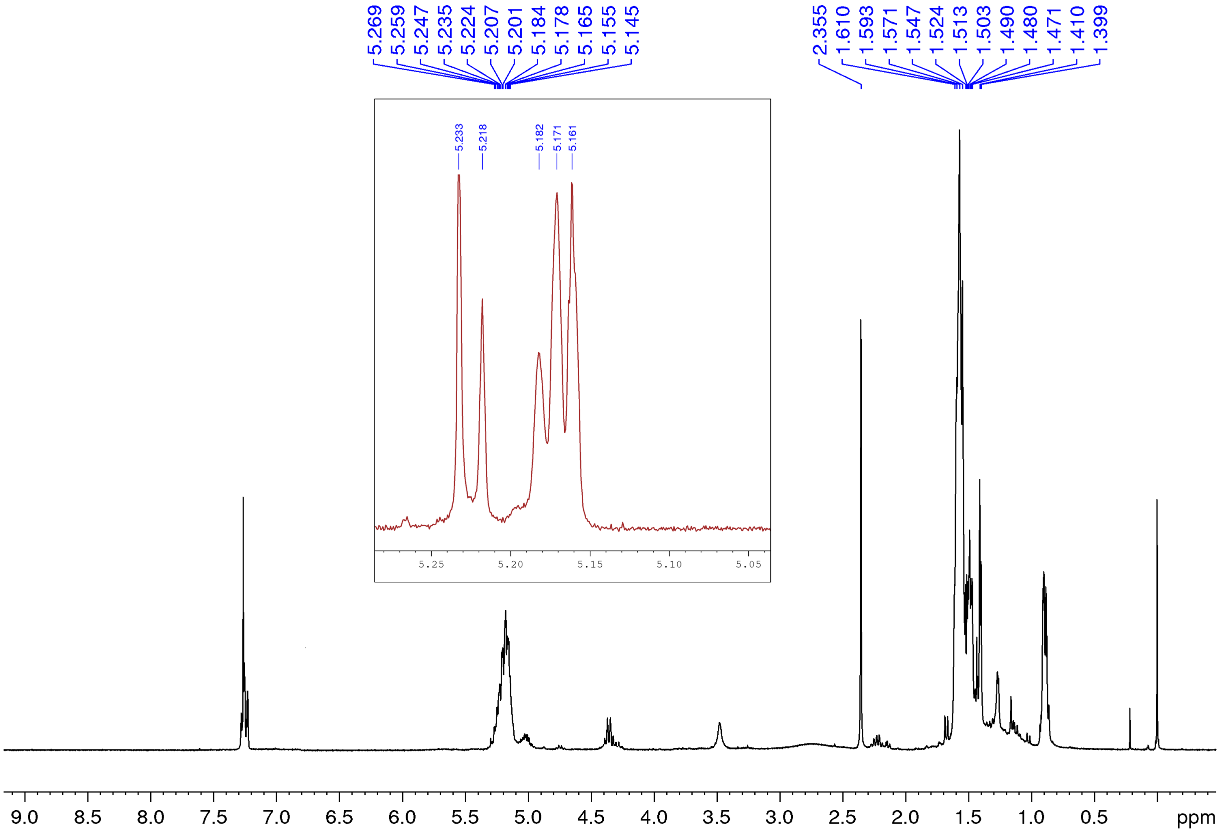

Figure S84. ^1H NMR and homodecoupled ^1H NMR spectrum of PLA prepared by $AlMe_3$ (2 M solution in toluene) in $CDCl_3$ [Reaction condition = {$[LA]_0/[Al]_0$ = 100:1}, 60 °C, 9h].

(S8) Characterization of polymers

TGA experiment of the polymers

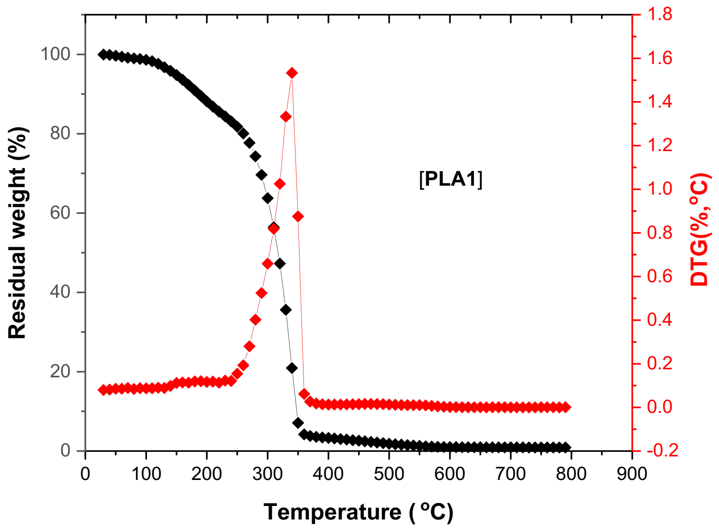

Figure S85. TGA and derivative thermogram of PLA formed by **4c** (Table 2, entry 12).

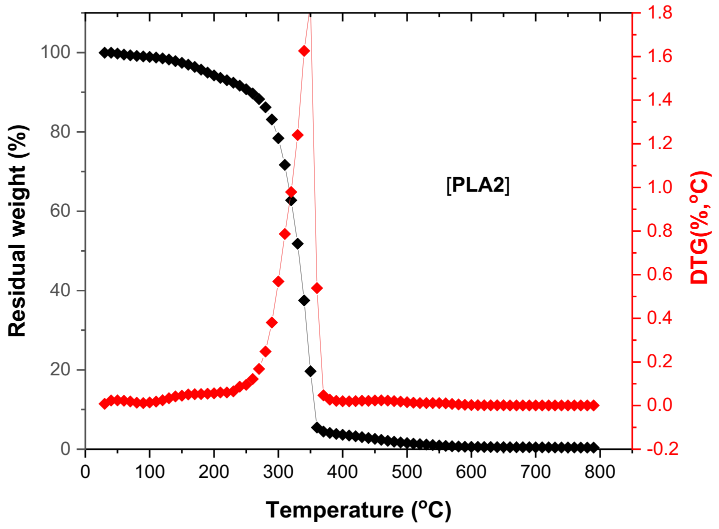

Figure S86. TGA and derivative thermogram of PLA formed by **4b** (Table 2, entry 6).

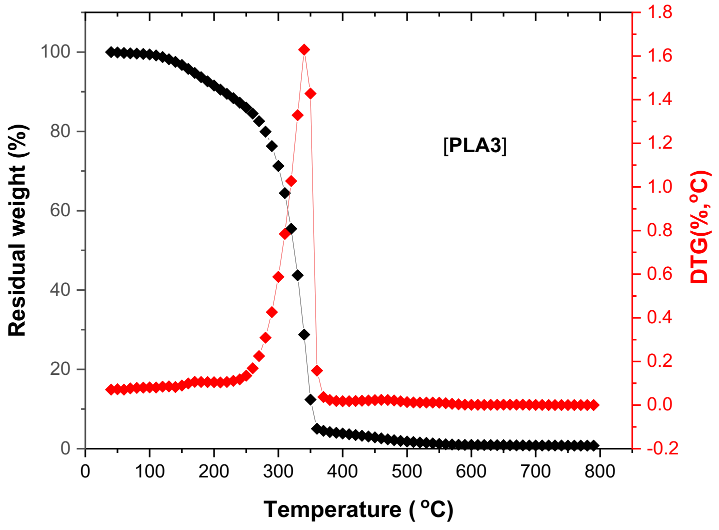

Figure S87. TGA and derivative thermogram of PLA formed by **4b** (Table 2, entry 7).

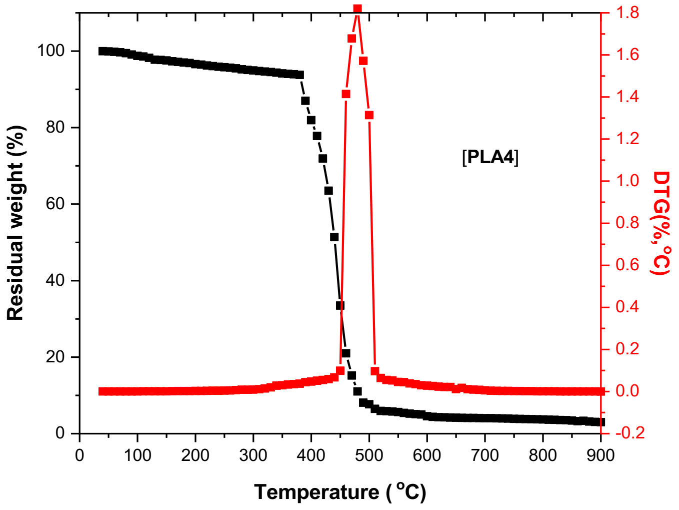

Figure S88. TGA and derivative thermogram of PLA formed by **4a** (Table 2, entry 1).

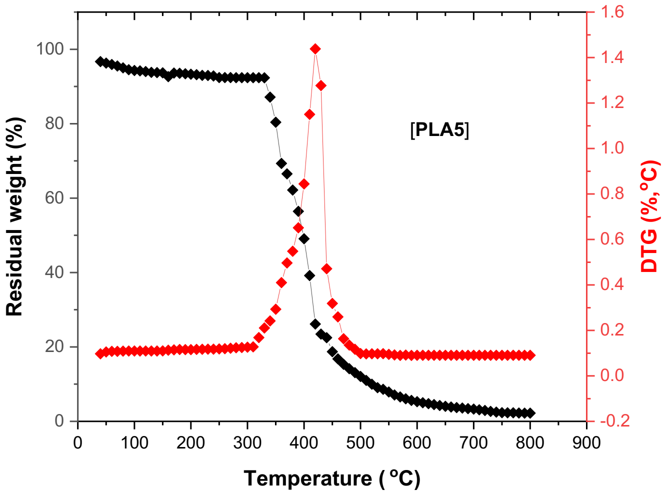

Figure S89. TGA and derivative thermogram of PLA formed by **4a** (Table 2, entry 3).

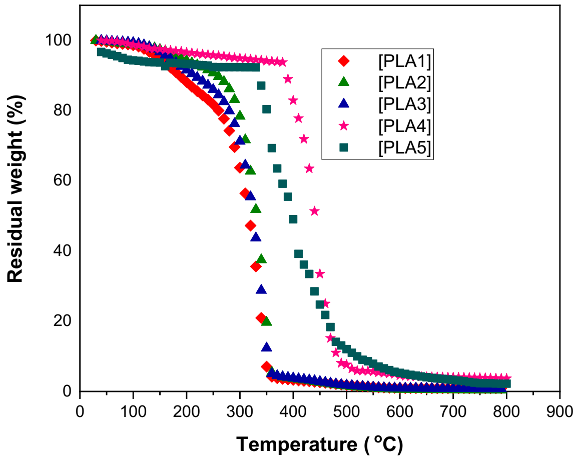

Figure S90. TGA thermograms of various composition of PLA

Table S14: Data from TGA thermograms of polylactide.

Sample	PLA entry from Table 1	T_{DTG} (0) (°C)	T_{DTG} (50) (°C)	T_{DTG} (Max) (°C)
PLA1	Entry 12, 200 mg *rac*-LA : **4c**	255	340	370
PLA2	Entry 6, 100 mg *rac*-LA : **4b**	270	350	390
PLA3	Entry 7, 300 mg *rac*-LA : **4b**	260	340	390
PLA4	Entry 1, 100 mg *rac*-LA : **4a**	385	450	520
PLA5	Entry 3, 300 mg *rac*-LA : **4a**	350	420	460

T_{DTG} = degradation temperature valued by weight loss derivative maximum of TGA thermogram.

T_{DTG} (0) = degradation temperature when zero weight loss of the poymer happen, T_{DTG} (50) = degradation temperature when 50% weight loss of the poymer happen, T_{DTG} (Max) = degradation temperature when maximum weight loss of the poymer happen.

DSC Experment of the polymers

T_g andT_m values were analyzed by differential scanning calorimetry (DSC).

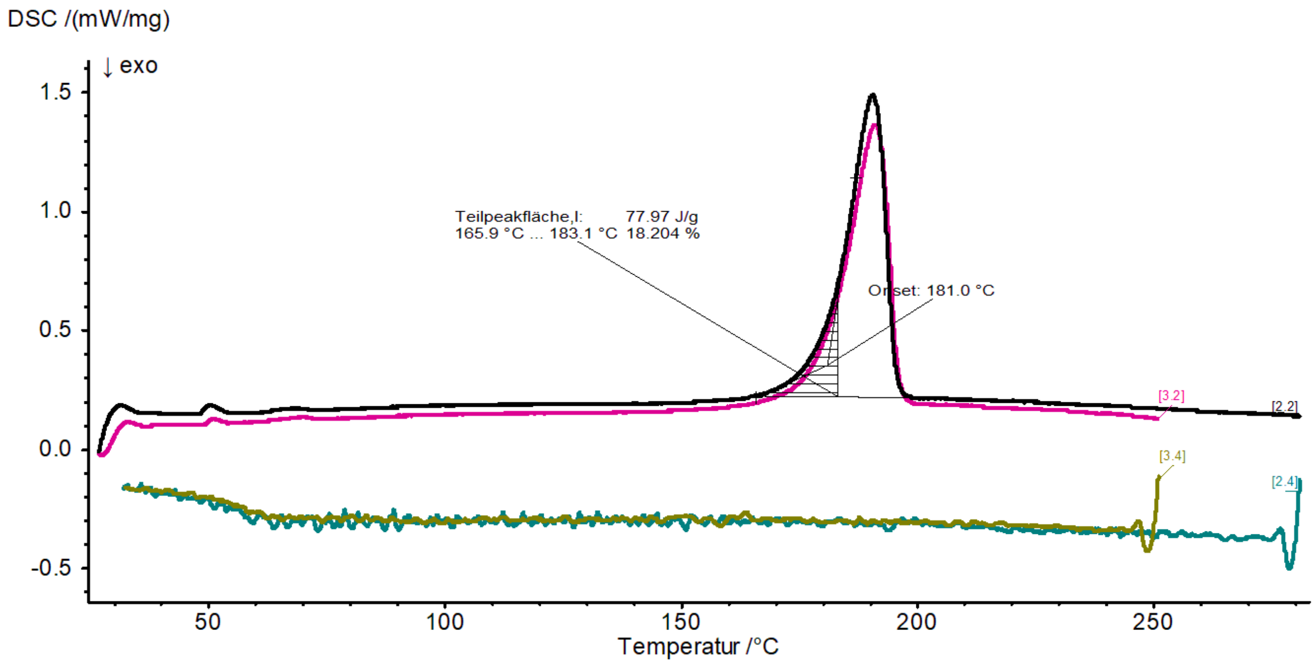

Figure S91. DSC Thermogram of second DSC heating run of PLA formed by **4a** (Table 2, entry 1).

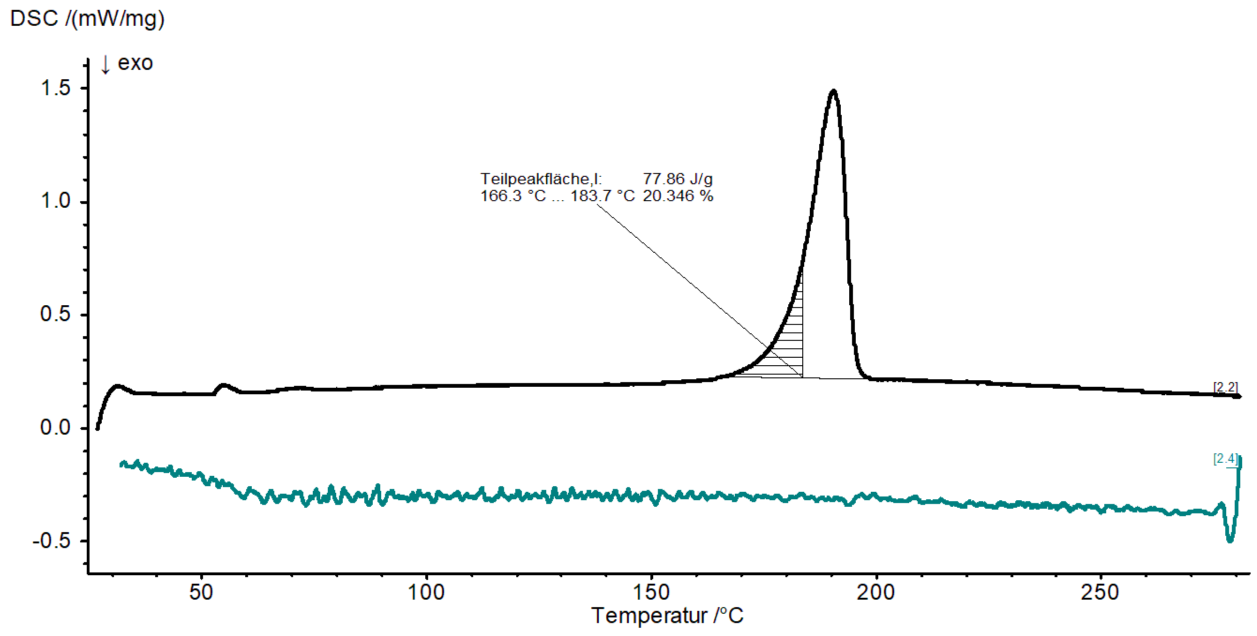

Figure S92. DSC Thermogram of second DSC heating run of PLA formed by **4a** (Table 2, entry 2).

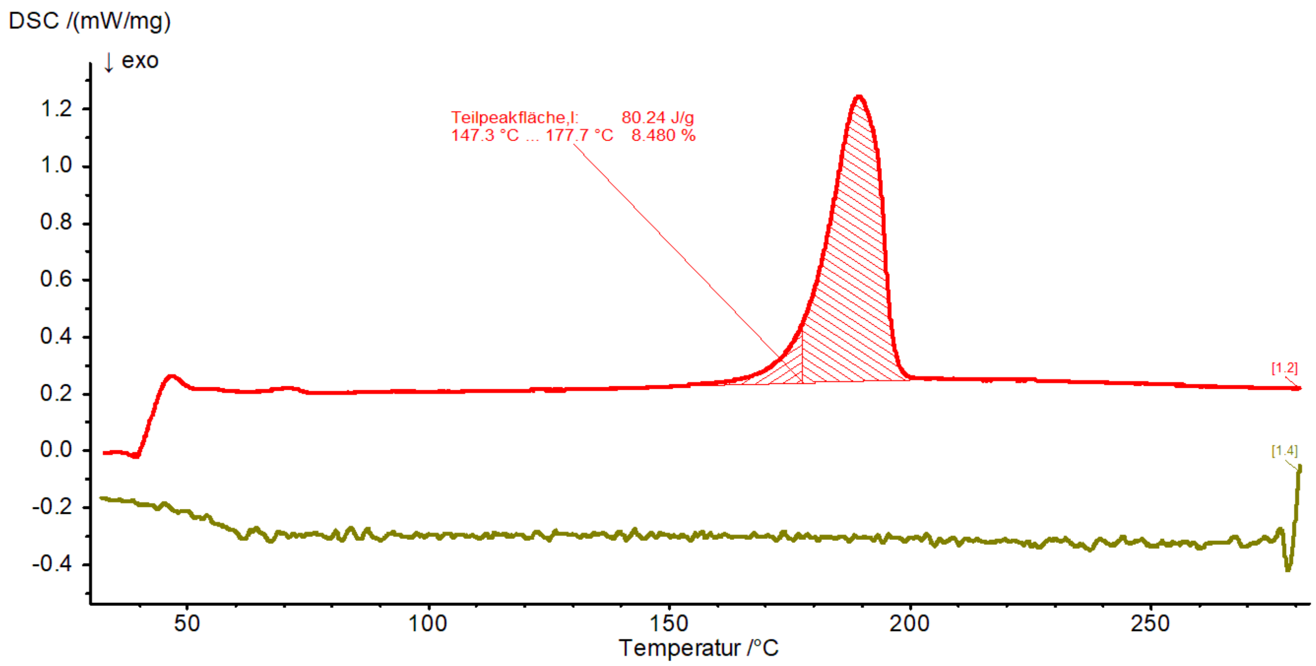

Figure S93. DSC Thermogram of second DSC heating run of PLA formed by **4a** (Table 2, entry 3).

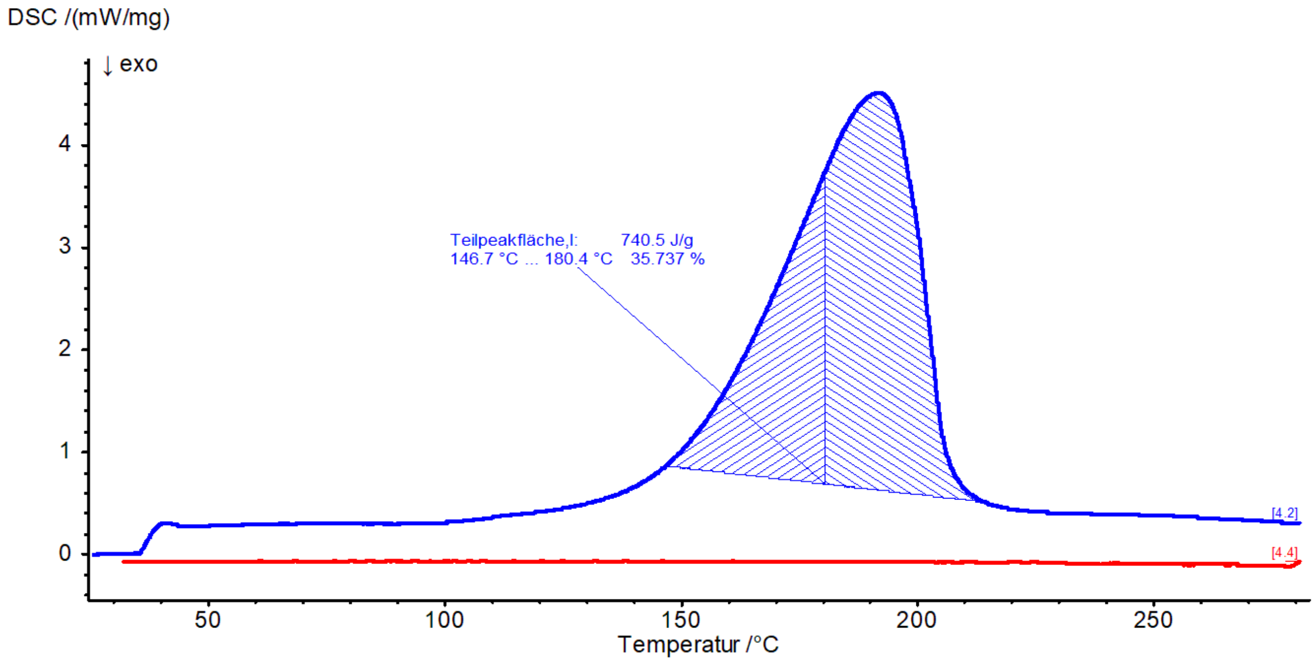

Figure S94. DSC Thermogram of second DSC heating run of PLA formed by **4a** (Table 2, entry 4).

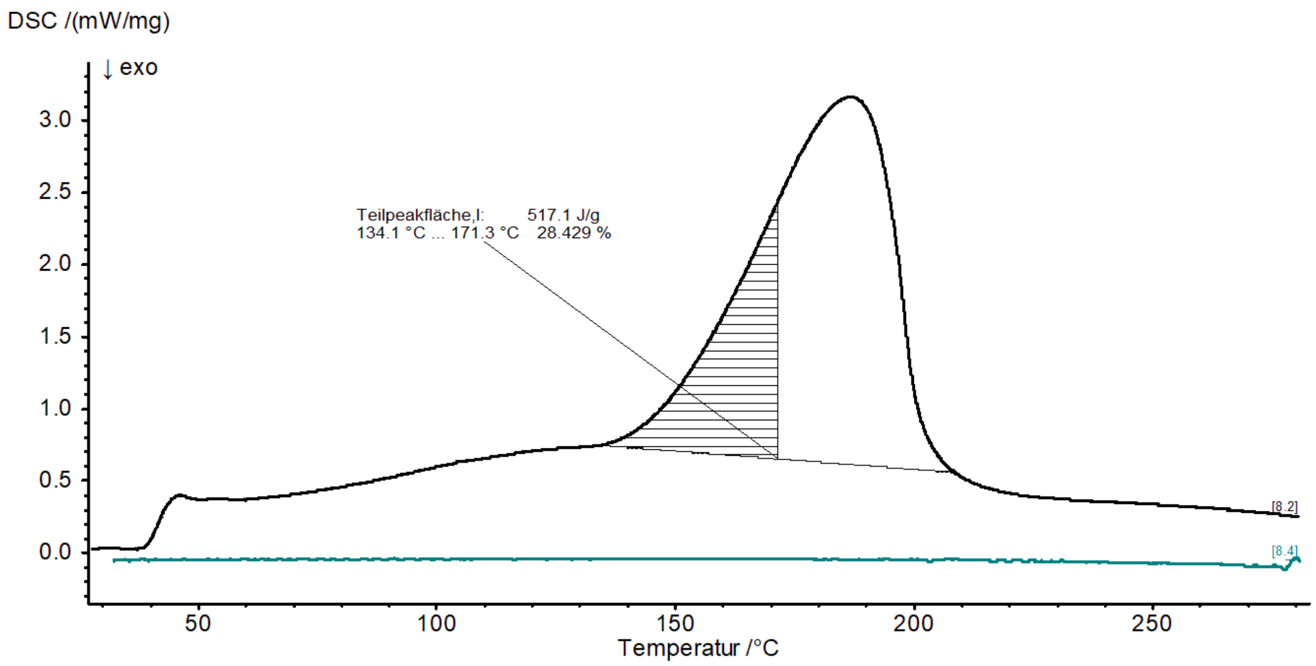

Figure S95. DSC Thermogram of second DSC heating run of PLA formed by **4a** (Table 2, entry 5).

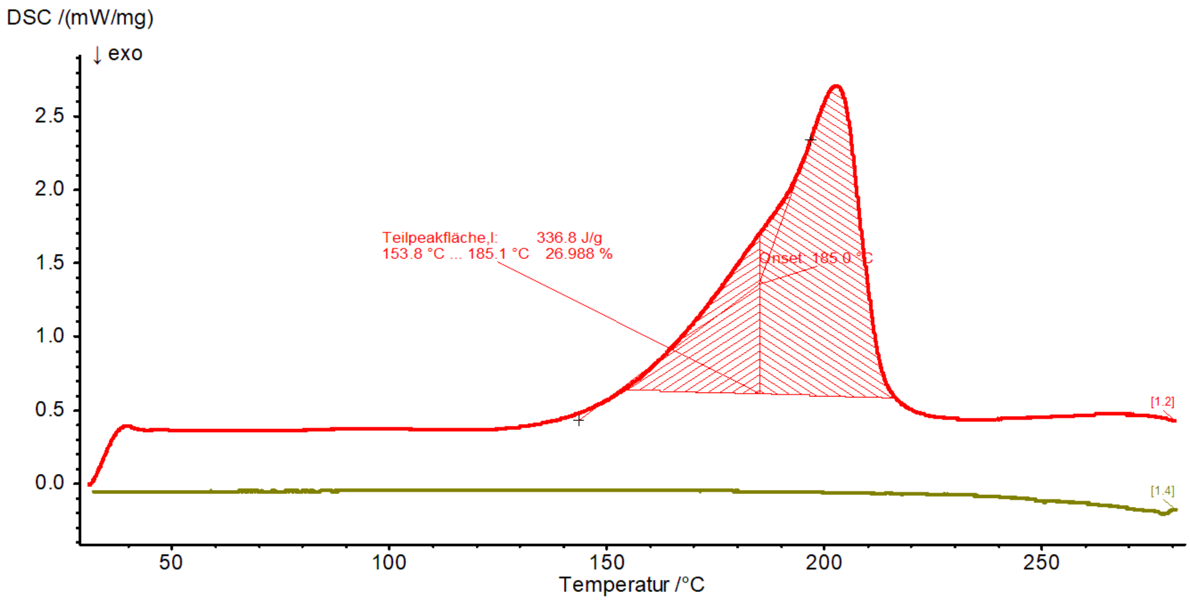

Figure S96. DSC Thermogram of second DSC heating run of PLA formed by **4b** (Table 2, entry 6).

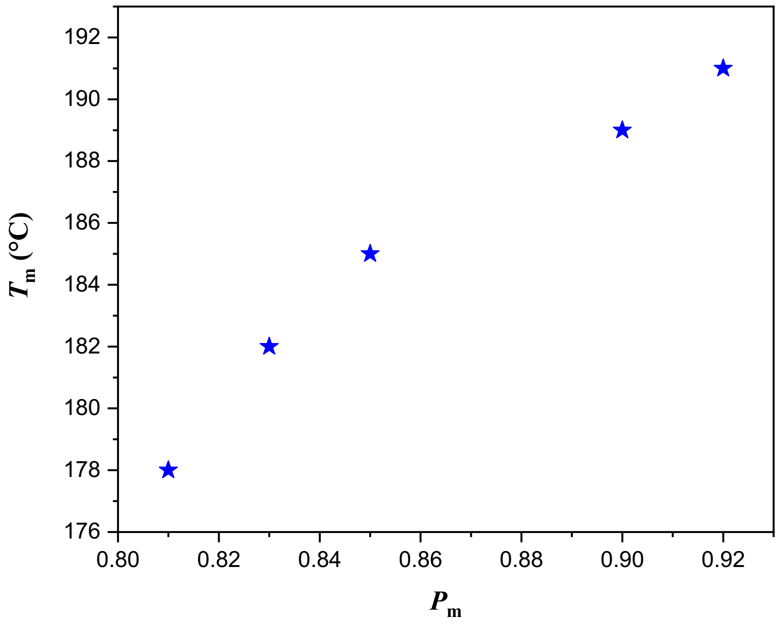

Figure S97. Relationship between the P_m values and the melting temperature (T_m) of PLA formed by **4a** (Nomura curve).[15]

Table S15: Data from DSC thermograms of PLA.

Entry	T_g (°C)	T_m (°C)
Table1, Entry 1	52	191
Table1, Entry2	52	189
Table1, Entry3	50	185
Table1, Entry4	49	182
Table1, Entry5	47	178

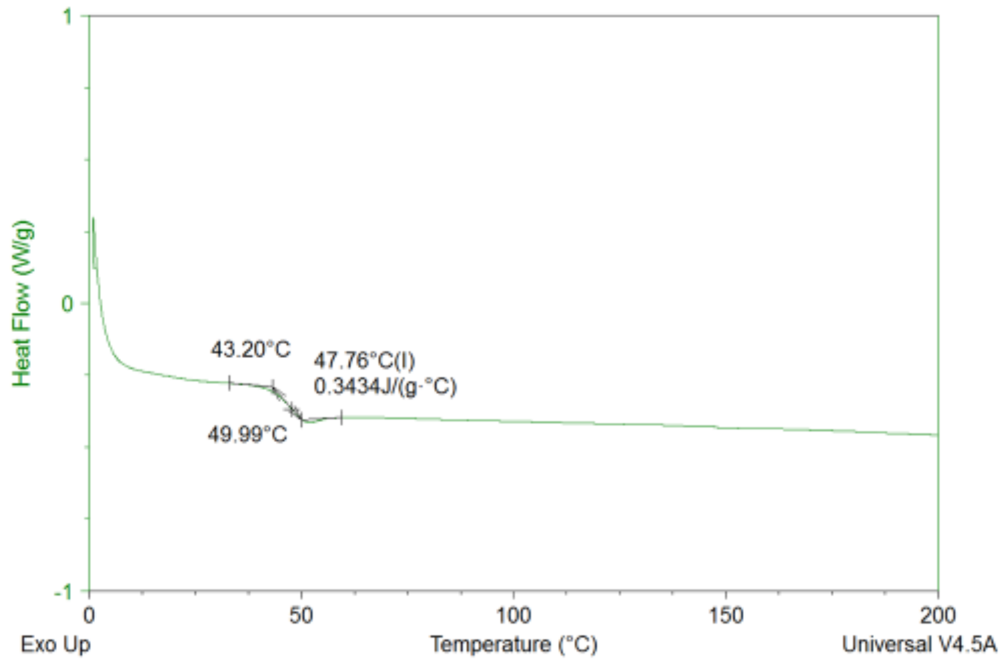

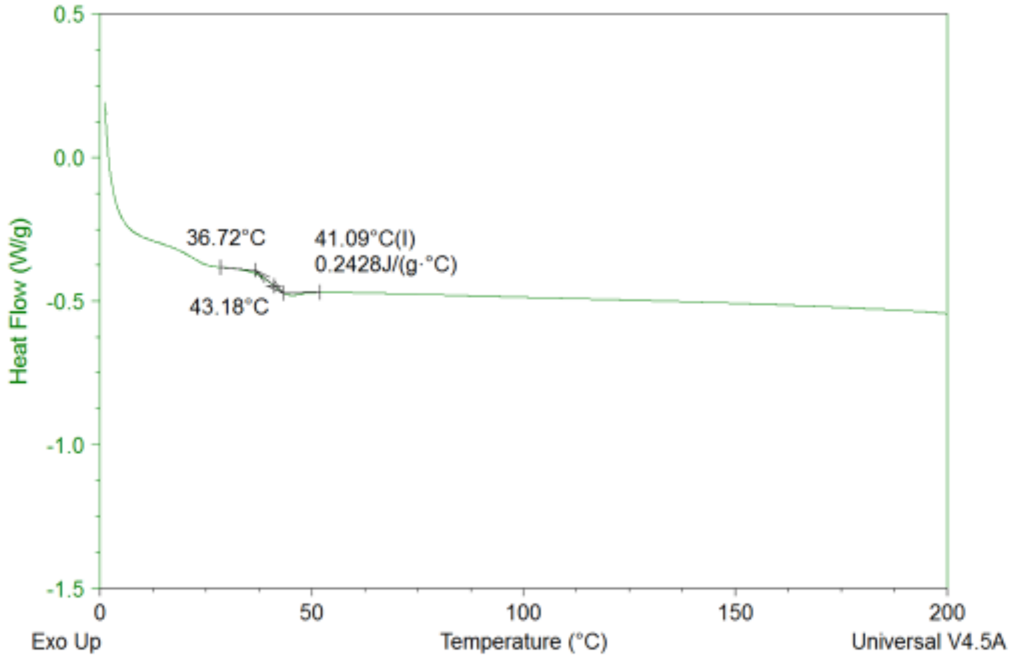

Figure S98. DSC Thermograms of PLA formed by **4c**, P_m < 0.60, (Table 2, Entry 11 and Entry 12). Second heating curve shown.

(S9) Single crystal X-ray structural data of compounds

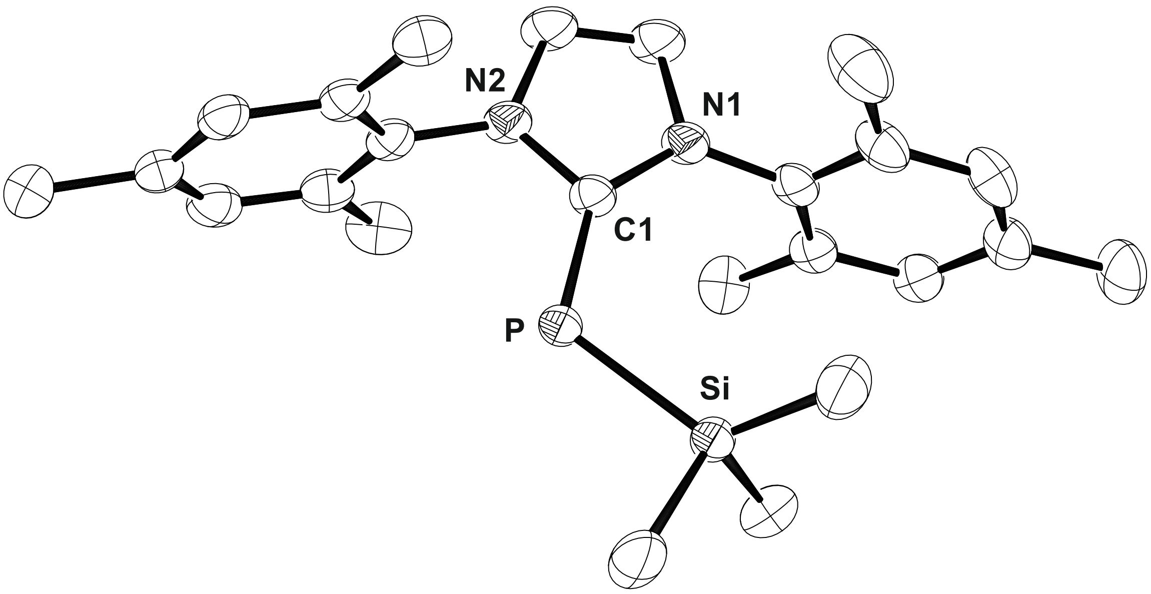

Figure S99. ORTEP diagram of **2b** with thermal displacement parameters drawn at 50% probability levels. Hydrogen atoms and solvent molecules were omitted for clarity.

Table S16. Crystal data and structure refinement for compound **2b**.

CCDC	2054479	
Empirical formula	$C_{24}H_{33}N_2PSi$	
Formula weight	408.58	
Temperature	100(2) K	
Wavelength	1.54184 Å	
Crystal system	Monoclinic	
Space group	$P2_1/n$	
Unit cell dimensions	a = 19.0170(7) Å	α= 90°
	b = 9.1324(4) Å	β= 95.355(4)°
	c = 28.2583(12) Å	γ = 90°
Volume	4886.2(3) $Å^3$	
Z	8	
Density (calculated)	1.111 Mg/m^3	
Absorption coefficient	1.535 mm^{-1}	

F(000)	1760
Crystal habitus	block (dichroic brown colourless)
Crystal size	0.295 x 0.254 x 0.129 mm^3
Theta range for data collection	2.689 to 77.459°
Index ranges	$-24 \le h \le 24$, $-11 \le k \le 10$, $-35 \le l \le 35$
Reflections collected	105602
Independent reflections	10229 [R_{int} = 0.0413]
Completeness to θ = 67.684°	99.9 %
Absorption correction	Gaussian
Max. and min. transmission	1.000 and 0.300
Refinement method	Full-matrix least-squares on F^2
Data / restraints / parameters	10229 / 0 / 523
Goodness-of-fit on F^2	1.076
Final R indices [I>2σ(I)]	R_1 = 0.0399, wR_2 = 0.1082
R indices (all data)	R_1 = 0.0410, wR_2 = 0.1090
Largest diff. peak and hole	0.278 and -0.351 e·$Å^{-3}$

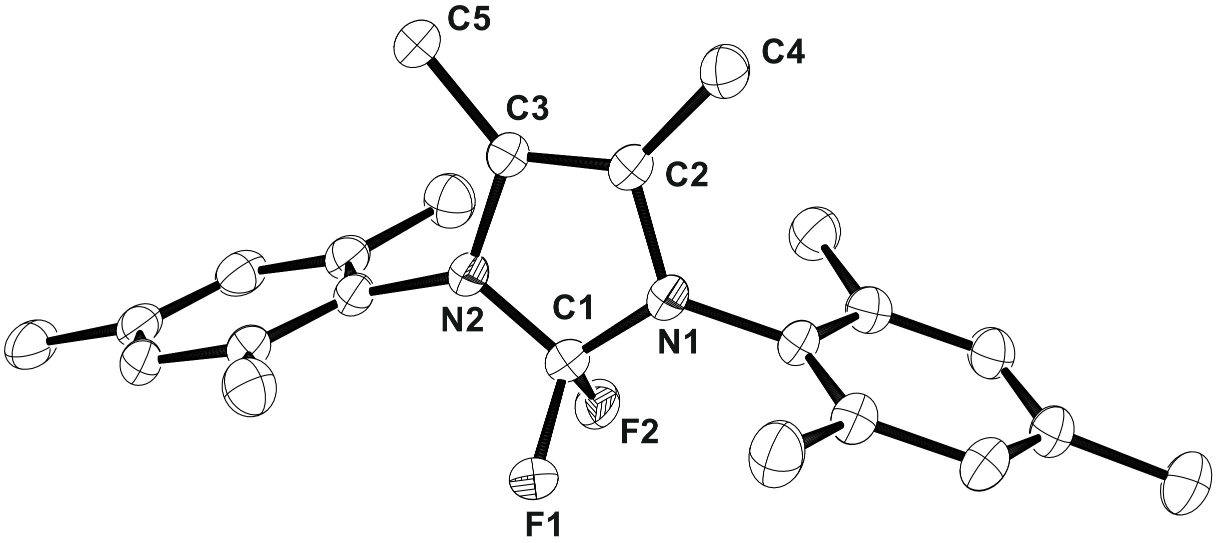

Figure S100. ORTEP diagram of **1c** with thermal displacement parameters drawn at 50% probability levels. Hydrogen atoms, solvent molecules and the minor component (18 %) of the disorder model for the cocrystallized oxidation product were omitted for clarity.

Table S17. Crystal data and structure refinement for compound **1c**.

CCDC	2054478	
Empirical formula	$C_{23}H_{28}F_{1.65}N_2O_{0.17}$	
Formula weight	366.67	
Temperature	100(2) K	
Wavelength	1.54184 Å	
Crystal system	Monoclinic	
Space group	$P2_1/n$	
Unit cell dimensions	a = 12.5557(5) Å	α= 90°
	b = 7.7569(3) Å	β= 97.401(4)°
	c = 20.5485(10) Å	γ = 90°
Volume	1984.61(15) Å^3	
Z	4	
Density (calculated)	1.227 Mg/m^3	
Absorption coefficient	0.667 mm^{-1}	
F(000)	785	
Crystal habitus	irregular (colourless)	

Crystal size	0.158 x 0.077 x 0.076 mm^3
Theta range for data collection	3.915 to 76.662°
Index ranges	-15 ≤ h ≤ 15, -9 ≤ k ≤ 9, -25 ≤ l ≤ 25
Reflections collected	50774
Independent reflections	4151 [R_{int} = 0.0614]
Completeness to θ = 67.684°	100.0 %
Absorption correction	Semi-empirical from equivalents
Max. and min. transmission	1.00000 and 0.49763
Refinement method	Full-matrix least-squares on F^2
Data / restraints / parameters	4151 / 0 / 257
Goodness-of-fit on F^2	1.042
Final R indices [I>2σ(I)]	R_1 = 0.0509, wR_2 = 0.1290
R indices (all data)	R_1 = 0.0600, wR_2 = 0.1359
Largest diff. peak and hole	0.499 and -0.381 e·$Å^{-3}$
A significant amount of residual electron density was found between the two fluorine atoms. The same has already been oberved for the IDipp analog Phenofluor®.[1] We assume that this can be attributed to a minor component (18 %) of cocrystallized oxidation product and was refined with the corresponding disorder model.	

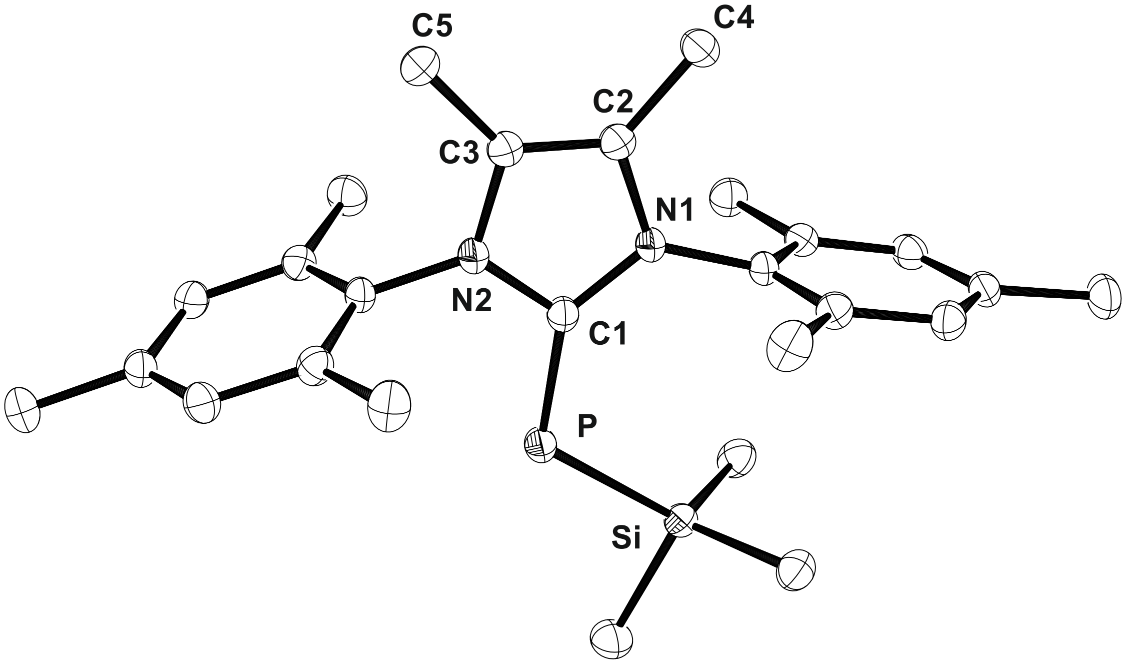

Figure S101. ORTEP diagram of **2c** with thermal displacement parameters drawn at 50% probability levels. Hydrogen atoms and solvent molecules were omitted for clarity.

Table S18. Crystal data and structure refinement for compound **2c**.

CCDC	2054480	
Empirical formula	$C_{26}H_{37}N_2PSi$	
Formula weight	436.63	
Temperature	100(2) K	
Wavelength	1.54184 Å	
Crystal system	Monoclinic	
Space group	$P2_1/c$	
Unit cell dimensions	a = 15.6976(4) Å	α= 90°
	b = 10.60635(16) Å	β= 117.010(3)°
	c = 17.2673(4) Å	γ = 90°
Volume	2561.33(11) Å^3	
Z	4	
Density (calculated)	1.132 Mg/m^3	
Absorption coefficient	1.493 mm^{-1}	
F(000)	944	

Crystal habitus	block (colourless)
Crystal size	0.226 x 0.154 x 0.102 mm^3
Theta range for data collection	5.065 to 76.228°
Index ranges	-17 ≤ h ≤ 19, -10 ≤ k ≤ 13, -21 ≤ l ≤ 20
Reflections collected	50949
Independent reflections	5366 [R_{int} = 0.0556]
Completeness to θ = 67.684°	100.0 %
Absorption correction	Semi-empirical from equivalents
Max. and min. transmission	1.00000 and 0.70819
Refinement method	Full-matrix least-squares on F^2
Data / restraints / parameters	5366 / 0 / 282
Goodness-of-fit on F^2	1.043
Final R indices [I>2σ(I)]	R_1 = 0.0331, wR_2 = 0.0898
R indices (all data)	R_1 = 0.0371, wR_2 = 0.0930
Largest diff. peak and hole	0.284 and -0.317 e·$Å^{-3}$

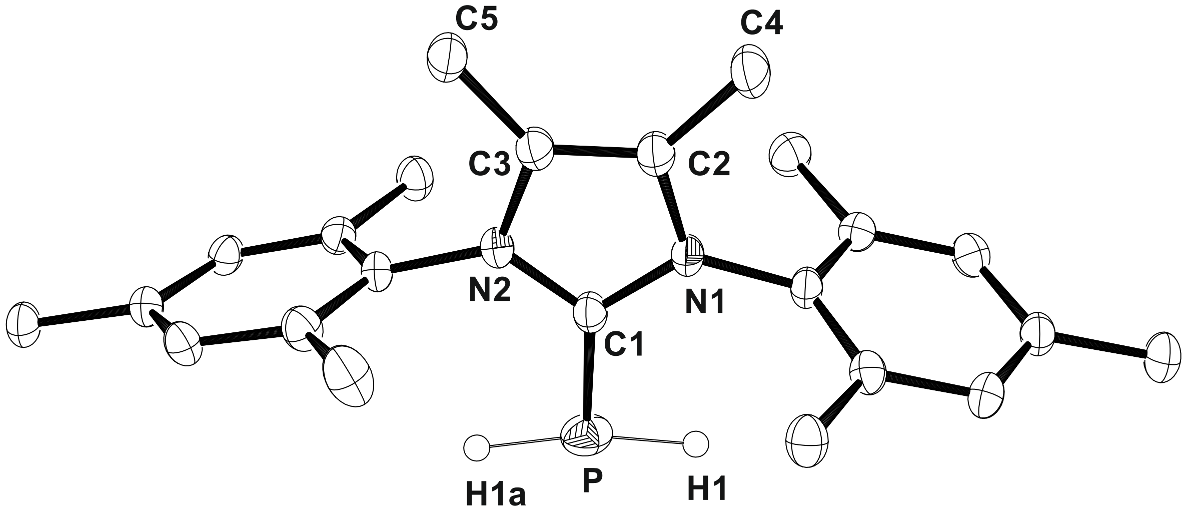

Figure S102. ORTEP diagram of **3c** with thermal displacement parameters drawn at 50% probability levels. Hydrogen atoms (except at phosphorus) and solvent molecules were omitted for clarity.

Table S19. Crystal data and structure refinement for compound **3c**.

CCDC	2054481	
Empirical formula	$C_{23}H_{29}N_2P$	
Formula weight	364.45	
Temperature	100(2) K	
Wavelength	1.54184 Å	
Crystal system	Triclinic	
Space group	$P\bar{1}$	
Unit cell dimensions	a = 8.5758(7) Å	α= 100.970(8)°
	b = 15.1768(16) Å	β= 93.807(7)°
	c = 16.8409(16) Å	γ = 101.884(8)°
Volume	2093.0(4) Å^3	
Z	4	
Density (calculated)	1.157 Mg/m^3	
Absorption coefficient	1.206 mm^{-1}	
F(000)	784	
Crystal habitus	irregular (colourless)	
Crystal size	0.112 x 0.071 x 0.025 mm^3	

Theta range for data collection	3.616 to 76.553°
Index ranges	-10 ≤ h ≤ 9, -19 ≤ k ≤ 18, -21 ≤ l ≤ 20
Reflections collected	39379
Independent reflections	8654 [R_{int} = 0.0631]
Completeness to θ = 67.684°	100.0 %
Absorption correction	Semi-empirical from equivalents
Max. and min. transmission	1.00000 and 0.69906
Refinement method	Full-matrix least-squares on F^2
Data / restraints / parameters	8654 / 6 / 503
Goodness-of-fit on F^2	1.042
Final R indices [I>2σ(I)]	R_1 = 0.0463, wR_2 = 0.1150
R indices (all data)	R_1 = 0.0638, wR_2 = 0.1279
Largest diff. peak and hole	0.292 and -0.332 e·Å^{-3}
The hydrogen atoms on phosphorus were refined as disordered over two positions with equal P-H-bond lengths (SADI).	

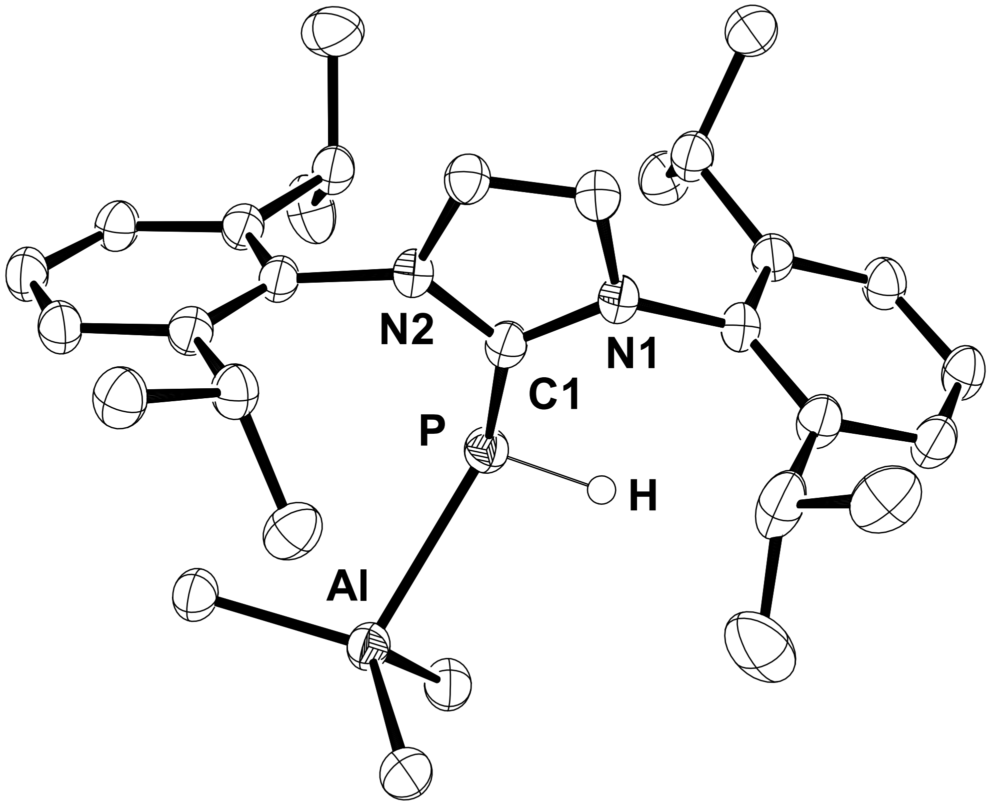

Figure S103. ORTEP diagram of **4a** with thermal displacement parameters drawn at 50% probability levels. Hydrogen atoms (except at phosphorus) and solvent molecules were omitted for clarity.

Table S21. Crystal data and structure refinement for compound **4a**.

CCDC	2054482	
Empirical formula	$C_{30}H_{46}AlN_2P$	
Formula weight	492.64	
Temperature	100(2) K	
Wavelength	1.54184 Å	
Crystal system	Triclinic	
Space group	$P\bar{1}$	
Unit cell dimensions	a = 9.8071(3) Å	α= 93.737(2)°
	b = 10.6661(3) Å	β= 107.109(2)°
	c = 14.9253(4) Å	γ = 91.522(2)°
Volume	1487.26(8) Å^3	
Z	2	
Density (calculated)	1.100 Mg/m^3	

Absorption coefficient	1.234 mm^{-1}
F(000)	536
Crystal habitus	irregular (colourless)
Crystal size	0.193 x 0.166 x 0.119 mm^3
Theta range for data collection	3.107 to 77.571°
Index ranges	-10 ≤ h ≤ 12, -13 ≤ k ≤ 13, -18 ≤ l ≤ 18
Reflections collected	62593
Independent reflections	6244 [R_{int} = 0.0450]
Completeness to θ = 67.684°	99.9 %
Absorption correction	Semi-empirical from equivalents
Max. and min. transmission	1.00000 and 0.56619
Refinement method	Full-matrix least-squares on F^2
Data / restraints / parameters	6244 / 0 / 322
Goodness-of-fit on F^2	1.048
Final R indices [I>2σ(I)]	R_1 = 0.0406, wR_2 = 0.1144
R indices (all data)	R_1 = 0.0429, wR_2 = 0.1167
Largest diff. peak and hole	0.465 and -0.254 e·$Å^{-3}$
The hydrogen atom H1 on P1 was refined freely.	

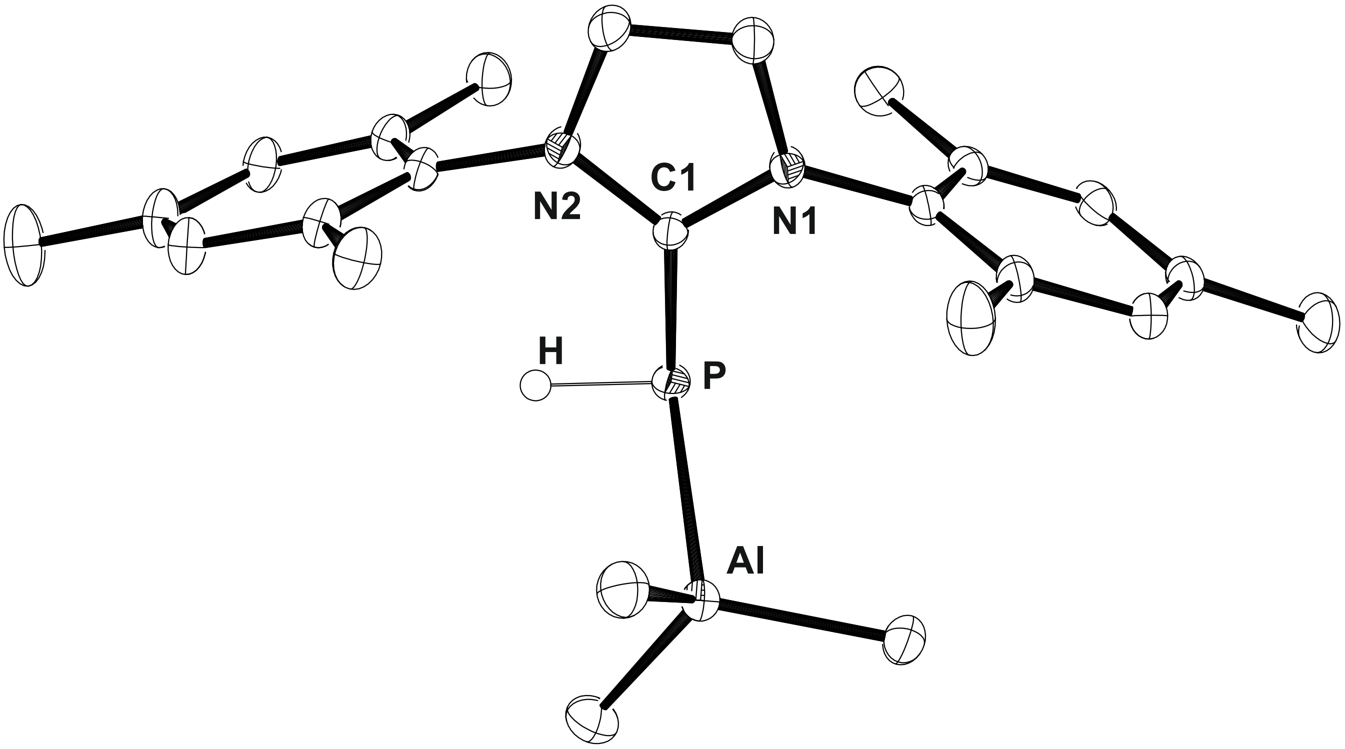

Figure S104. ORTEP diagram of **4b** with thermal displacement parameters drawn at 50% probability levels. Hydrogen atoms (except at phosphorus) and solvent molecules were omitted for clarity.

Table S23. Crystal data and structure refinement for compound **4b**.

CCDC	2054483	
Empirical formula	$C_{24}H_{34}AlN_2$ P	
Formula weight	408.48	
Temperature	100(2) K	
Wavelength	0.71073 Å	
Crystal system	Orthorhombic	
Space group	*Pbca*	
Unit cell dimensions	a = 9.6344(3) Å	α= 90°
	b = 16.7467(4) Å	β= 90°
	c = 29.7415(9) Å	γ = 90°
Volume	4798.6(2) $Å^3$	
Z	8	
Density (calculated)	1.131 Mg/m^3	

Absorption coefficient	0.163 mm^{-1}
F(000)	1760
Crystal habitus	irregular (orange)
Crystal size	0.633 x 0.459 x 0.384 mm^3
Theta range for data collection	2.533 to 44.833°
Index ranges	-19 ≤ h ≤ 19, -33 ≤ k ≤ 33, -58 ≤ l ≤ 56
Reflections collected	173929
Independent reflections	19549 [R_{int} = 0.0533]
Completeness to θ = 25.242°	99.8 %
Absorption correction	Gaussian
Max. and min. transmission	1.000 and 0.260
Refinement method	Full-matrix least-squares on F^2
Data / restraints / parameters	19549 / 0 / 266
Goodness-of-fit on F^2	1.061
Final R indices [I>2σ(I)]	R_1 = 0.0497, wR_2 = 0.1327
R indices (all data)	R_1 = 0.0709, wR_2 = 0.1421
Largest diff. peak and hole	0.681 and -0.302 e·$Å^{-3}$
The hydrogen atom H1 on P1 was refined freely.	

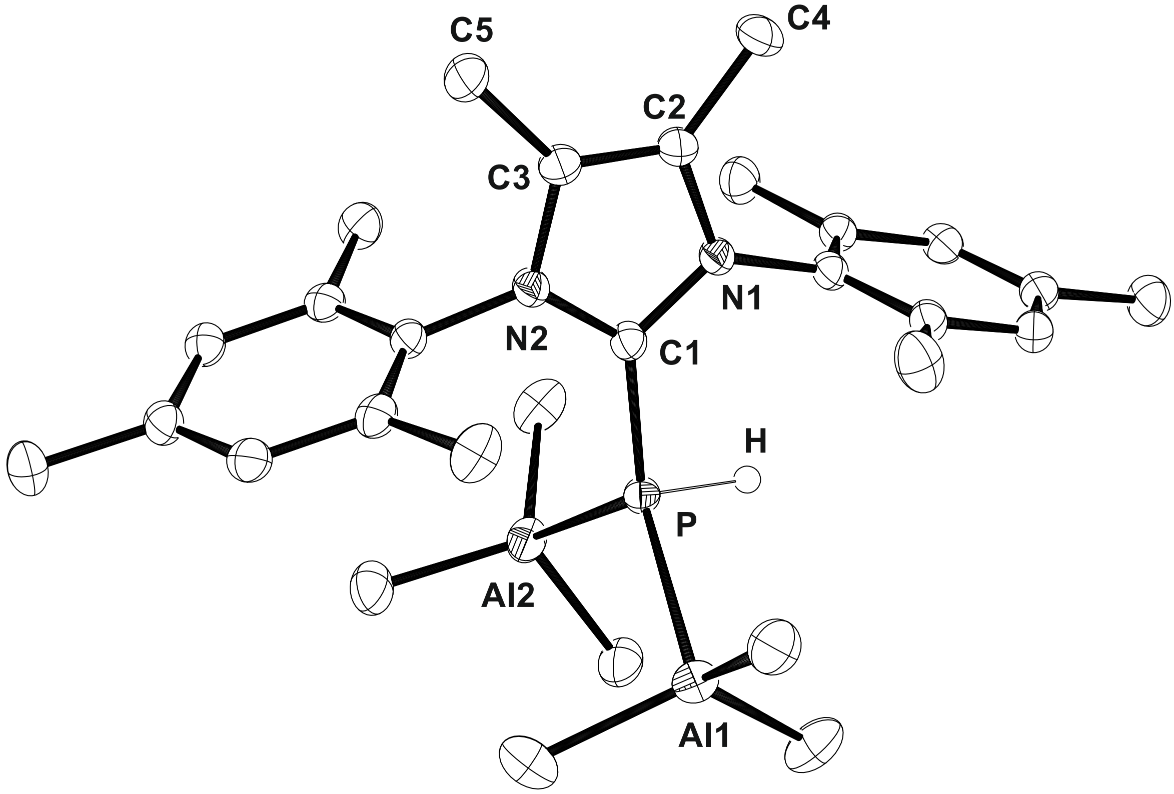

Figure S105. ORTEP diagram of **4c** with thermal displacement parameters drawn at 50% probability levels. Hydrogen atoms (except at phosphorus) and solvent molecules were omitted for clarity.

Table S25. Crystal data and structure refinement for compound **4c**.

Identification code	2054484	
Empirical formula	$C_{29}H_{47}Al_2N_2P$	
Formula weight	508.61	
Temperature	100(2) K	
Wavelength	1.54184 Å	
Crystal system	Trigonal	
Space group	*P*3$_1$	
Unit cell dimensions	a = 10.27240(10) Å	α= 90°
	b = 10.27240(10) Å	β= 90°
	c = 25.9609(4) Å	γ = 120°
Volume	2372.43(6) Å^3	
Z	3	
Density (calculated)	1.068 Mg/m^3	

Absorption coefficient	1.429 mm^{-1}
F(000)	828
Crystal habitus	irregular (yellow)
Crystal size	0.279 x 0.162 x 0.120 mm^3
Theta range for data collection	4.971 to 77.639°
Index ranges	-12 ≤ h ≤ 12, -12 ≤ k ≤ 12, -32 ≤ l ≤ 28
Reflections collected	105904
Independent reflections	6519 [R_{int} = 0.0970]
Completeness to θ = 67.684°	100.0 %
Absorption correction	Gaussian
Max. and min. transmission	1.000 and 0.600
Refinement method	Full-matrix least-squares on F^2
Data / restraints / parameters	6519 / 1 / 325
Goodness-of-fit on F^2	1.067
Final R indices [I>2σ(I)]	R_1 = 0.0569, wR_2 = 0.1455
R indices (all data)	R_1 = 0.0571, wR_2 = 0.1457
Largest diff. peak and hole	0.312 and -0.589 e·Å^{-3}
The hydrogen atom H1 on P1 was refined freely.	

(S10) References

[1] A. Doddi, D. Bockfeld, T. Bannenberg, P. G. Jones, M. Tamm, *Angew. Chem. Int. Ed.* **2014**, *53*, 13568–13572; *Angew. Chem.* **2014**, *126*, 13786.

[2] Marcus L. Cole, Cameron Jones, Peter C. Junk, *New J. Chem.* **2002**, *26*, 1296–1303.

[3] A. Doddi, M. Weinhart, A. Hinz, D. Bockfeld, J. M. Goicoechea, M. Scheer, M. Tamm, *Chem. Commun.* **2017**, *53*, 6069–6072.

[4] K. Hirano, S. Urban, C. Wang, F. Glorius, *Org. Lett.* **2009**, *11*, 1019–1022.

[5] Rigaku Oxford Diffraction, *CrysAlisPRO Software System*, versions 1.171.39.46 (**1c**, **2c**, **3c**), 1.171.40.61a (**2b**, **4b**, **4c**) and 1.171.40.61a (**4a**), Rigaku Corporation, Oxford, UK.

[6] G. M. Sheldrick, *Acta Cryst.* **2015**, *A71*, 3–8.

[7] G. M. Sheldrick, *Acta Cryst.* **2015**, *C71*, 3–8.

[8] O. V. Dolomanov, L. J. Bourhis, R. J. Gildea, J. A. K. Howard, H. Puschmann, *J. Appl. Cryst.* **2009**, *42*, 339–341.

[9] K. A.M. Thakur, R. T. Kean, E. S. Hall, J. J. Kolstad, T. A. Lindgren, M. A. Doscotch, J. I. Siepmann, E. J. Munson, *Macromolecules* **1997**, *30*, 2422–2428.

[10] F. A. Bovey, P. A. Mirau, *NMR of polymers*, Acad. Press, San Diego, Calif., **1996**.

[11] J. Coudane, C. Ustariz-Peyret, G. Schwach, M. Vert, *J. Polym. Sci. A Polym. Chem.* **1997**, *35*, 1651–1658.

[12] Tina M. Ovitt, Geoffrey W. Coates, *J. Polym. Sci. A Polym. Chem.* **2000**, *38*, 4686–4692.

[13] T. M. Ovitt, G. W. Coates, *J. Am. Chem. Soc.* **2002**, *124*, 1316–1326.

[14] A. F. Douglas, B. O. Patrick, P. Mehrkhodavandi, *Angew. Chem. Int. Ed.* **2008**, *47*, 2290.

[15] N. Nomura, J. Hasegawa, R. Ishii, *Macromolecules* **2009**, *42*, 4907–4909.
